# Supplementary material for: Systematic identification of variant-specific RNA structure-small molecule interactions exemplified by RNA G-quadruplexes
Source: Nat Commun. 2026 Mar 19;17:2243. doi: 10.1038/s41467-026-70097-9 (PMC13002888; doi:10.1038/s41467-026-70097-9)
Supplement: Supplementary file 1 — Supplementary Information [file 41467_2026_70097_MOESM1_ESM.pdf]

Supplementary Information

Systematic identification of variant-specific RNA structure-small molecule interactions exemplified by RNA G-quadruplexes

Emi Miyashita[1,2], Kazumitsu Onizuka[3*], Yutong Chen[3], Hiroki Yoshida[2], Hina Hatayama[3], Shunya Ishikawa[3], Peijie Yan[2,4], Takahito Hasegawa[3], Mamiko Ozawa[3], Kaho Maeta[2], Fumi Nagatsugi[3], Hirohide Saito[1,4*], Kaoru R. Komatsu[2*]

[1]Center for iPS Cell Research and Application, Kyoto University, Kyoto, 606-8507, Japan

[2] xFOREST Therapeutics Co., Ltd., Kyoto, 602-0841, Japan

[3] Institute of Multidisciplinary Research for Advanced Materials, Tohoku University, Miyagi, 980-8577, Japan

[4] Institute for Quantitative Bioscience, The University of Tokyo, Tokyo, 113-0032, Japan

*Correspondence: krk@xforestx.com (K.R.K.), saitou.hirohide.8a@kyoto-u.ac.jp (H.S.), and onizuka@tohoku.ac.jp (K.O.)

Contents

Supplementary Figures

- Supplementary Fig. 1 | VQ stacks with the −1 cDNA base, possibly facilitating reverse transcription deletion
- Supplementary Fig. 2 | MALDI-TOF mass spectrum of alkylated RNA
- Supplementary Fig. 3 | Modification-derived RT mutation rate with two RT enzymes
- Supplementary Fig. 4 | Detecting loop-specific binding of SMN-C2 by BIVID-MaP
- Supplementary Fig. 5 | Deletion detected by BIVID-MaP is derived from binding-dependent covalent modifications
- Supplementary Fig. 6 | Deletion length profiles at each nucleotide position
- Supplementary Fig. 7 | Gel shift assay showing modifications to RNA structure motifs by Berberine-VQ-N_3 (NMe_2)
- Supplementary Fig. 8 | BIVID-MaP with an enrichment step for modified RNA for large-scale analysis
- Supplementary Fig. 9 | Adding pull-down step reduced necessary read depth for BIVID-MaP
- Supplementary Fig. 10 | Comparison of BIVID-MaP, SHAPE-MaP and DMS-MaPseq
- Supplementary Fig. 11 | Comparison of BIVID-MaP, SHAPE-MaP, and DMS-MaPseq using VQ-conjugated berberine
- Supplementary Fig. 12 | Reactivities for negative control RNAs in BIVID-MaP, SHAPE-MaP and DMS-MaPseq
- Supplementary Fig. 13 | Comparison of BIVID-MaP, SHAPE-MaP, and DMS-MaPseq to detect multiple RNA structure-small molecule interactions
- Supplementary Fig. 14 | G4 structure-specific interaction with berberine modulated by G to A mutation
- Supplementary Fig. 15 | SNV-specific RNA-small molecule interactions were not detected by either SHAPE-MaP or DMS-MaPseq
- Supplementary Fig. 16 | Heat map of RT-deletion changes in 5′ UTRs caused by somatic mutations
- Supplementary Fig. 17 | Normalized RT-deletion of all analyzed variants in *DAXX* or *ING2*
- Supplementary Fig. 18 | RT-deletion profiles showing variant-specific interactions between 5′ UTRs and berberine
- Supplementary Fig. 19 | RT-deletion profiles of 5′ UTR variants with CMA-VQ-N_3 (NMe_2) or VQ-N_3
- Supplementary Fig. 20 | G4 structure prediction by multiple software tools for the 5′ UTR variants
- Supplementary Fig. 21 | Validation of RNA structures by CD spectrum analysis
- Supplementary Fig. 22 | Fluorescence emission of NMM binding to RNA in the presence of K^+ or Li^+ ions

- Supplementary Fig. 23 | Predicted RNA structure of the *ING2* 5′ UTR
- Supplementary Fig. 24 | SNV-induced RNA structural alteration and its effects on berberine binding
- Supplementary Fig. 25 | RT substitution produces false positives rather than RT deletion
- Supplementary Fig. 26 | RT deletion profiling of sequences with two variants
- Supplementary Fig. 27 | Shortening modification time by pre-incubation of VQ precursor enables cell lysate application

Supplementary Notes

- MALDI-TOF analysis indicates that the multiple gel-shift bands originate from 1:1 and 1:2 Rbs-VQ:RNA labeling stoichiometries
- Enrichment of modified RNA to increase RT-deletion aimed at large-scale analysis, related to Supplementary Fig. 8
- The mechanistic differences between mutational profiling approaches can impact RNA-small molecule interaction detection
- Detecting the berberine-5′UTR interaction with sequences harboring two SNVs, related to Supplementary Fig. 26
- Detailed information on the preparation of the 5′UTR somatic mutation library
- Detailed information on the deletion profiling analysis of the 5′UTR somatic mutation library
- Synthesis of VQ-conjugated compounds
- NMR data

Supplementary References

Supplementary Datasets (.xlsx)

- Supplementary Data 1: 5′ UTR somatic mutation library
- Supplementary Data 2: Single-RNA library for BIVID-MaP
- Supplementary Data 3: G4 and nonG4 containing library for BIVID-MaP
- Supplementary Data 4: RNA sequences for gel shift assay
- Supplementary Data 5: RNA sequences for CD-spectrum measurement
- Supplementary Data 6: RNA sequences for AS-MS
- Supplementary Data 7: RNA sequences for G4 sensing assay

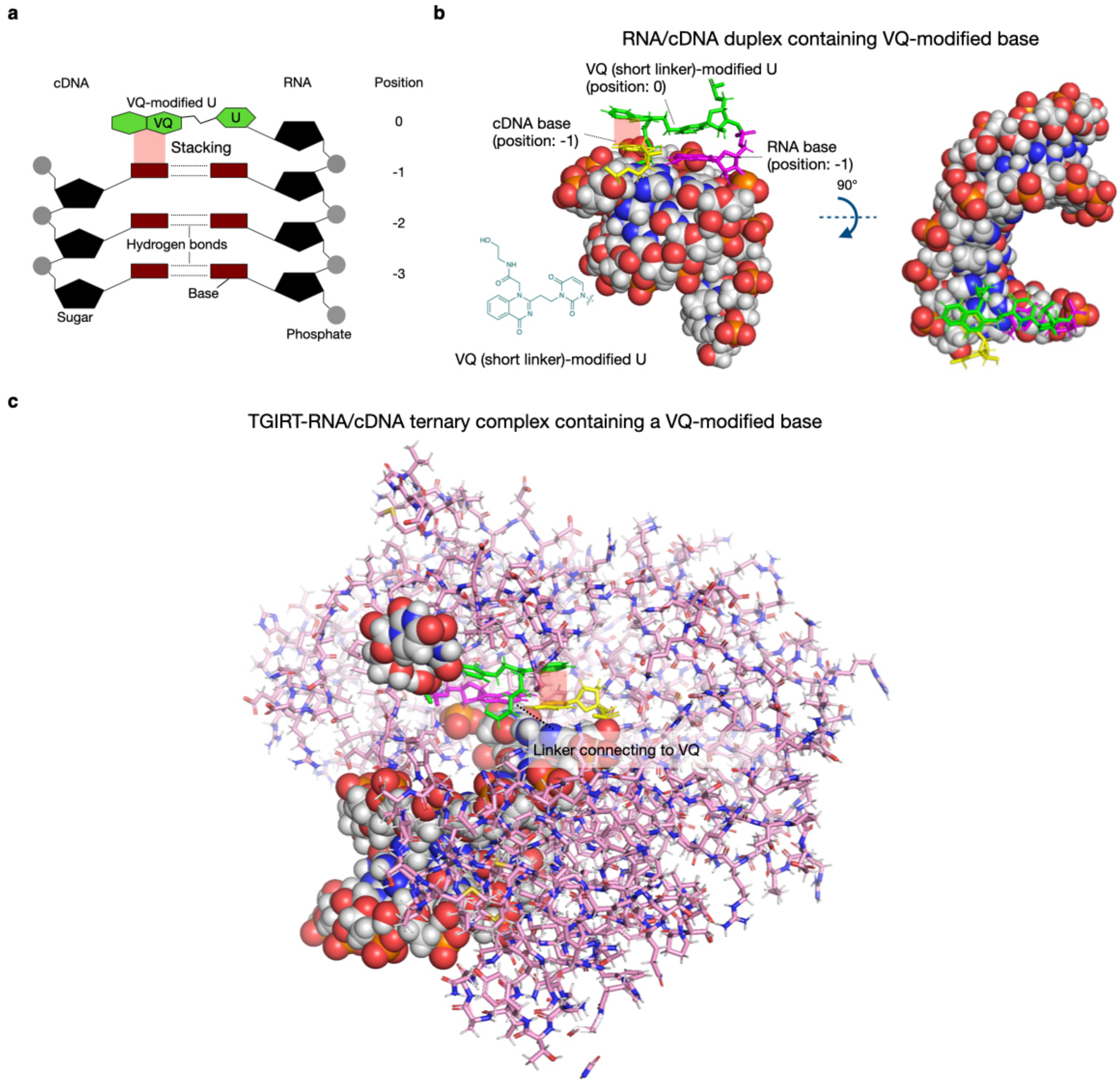

Supplementary Fig. 1 | VQ stacks with the –1 cDNA base, possibly facilitating reverse transcription deletion

(a) Conceptual diagram of VQ stacking with the –1 cDNA base. The planar VQ potentially stabilizes a conformation with a one-nucleotide deletion at position 0. **(b)** Representative conformation of the RNA/cDNA duplex containing a VQ-modified uridine, derived from MD simulations. Light red shading indicates stacking between the VQ-modified uridine (0) and the cDNA base (–1). **(c)** Representative conformation of the TGIRT-RNA/cDNA ternary complex containing a VQ-modified base, derived from MD simulations. The initial structure was modeled by MacroModel based on the reported crystal structure [6AR1][1]. An accessible pocket at the VQ-linker terminus potentially accommodates the target small molecule. Light red shading indicates stacking between the VQ-modified uridine (0) and the cDNA base (–1).

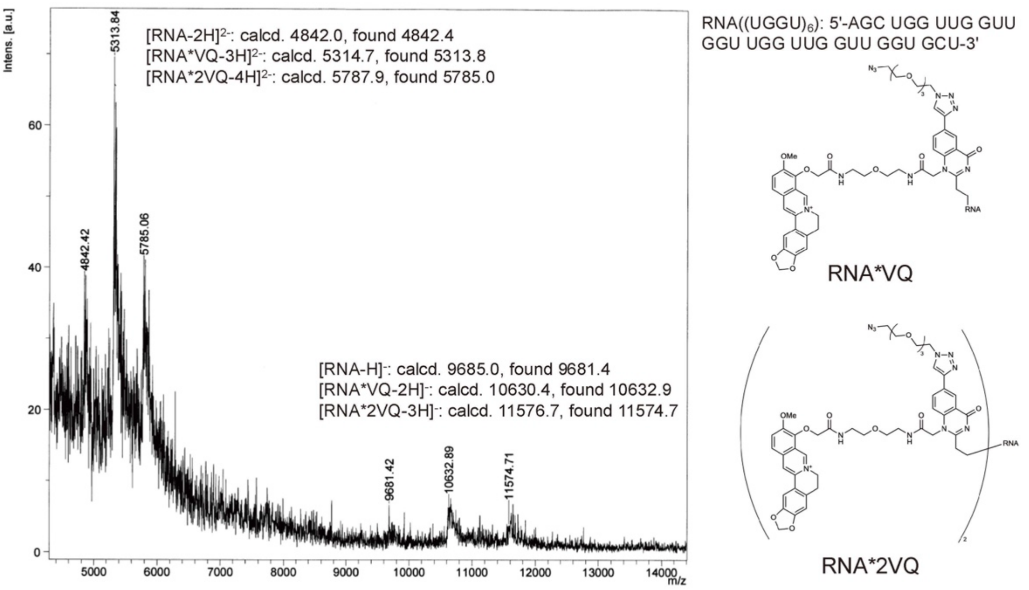

Supplementary Fig. 2 | MALDI-TOF mass spectrum of alkylated RNA

After an 8 h alkylation reaction of G4 (UGGU)6 with Berberine-VQ-N_3 (NMe2), the RNAs were purified using a NAP$_{TM}$-5 column (Cytiva) and desalted with a Sep-Pak C18 Plus short cartridge (Waters$_{TM}$), followed by lyophilization. The resulting dry RNAs were then dissolved in H_2O (10 μL) and passed through a Zip-Tip® pipette tip (Merck Millipore) to prepare the sample for mass spectrometry. MALDI-TOF mass spectrometry revealed the presence of non-alkylated RNA, along with RNAs corresponding to one and two alkylation events.

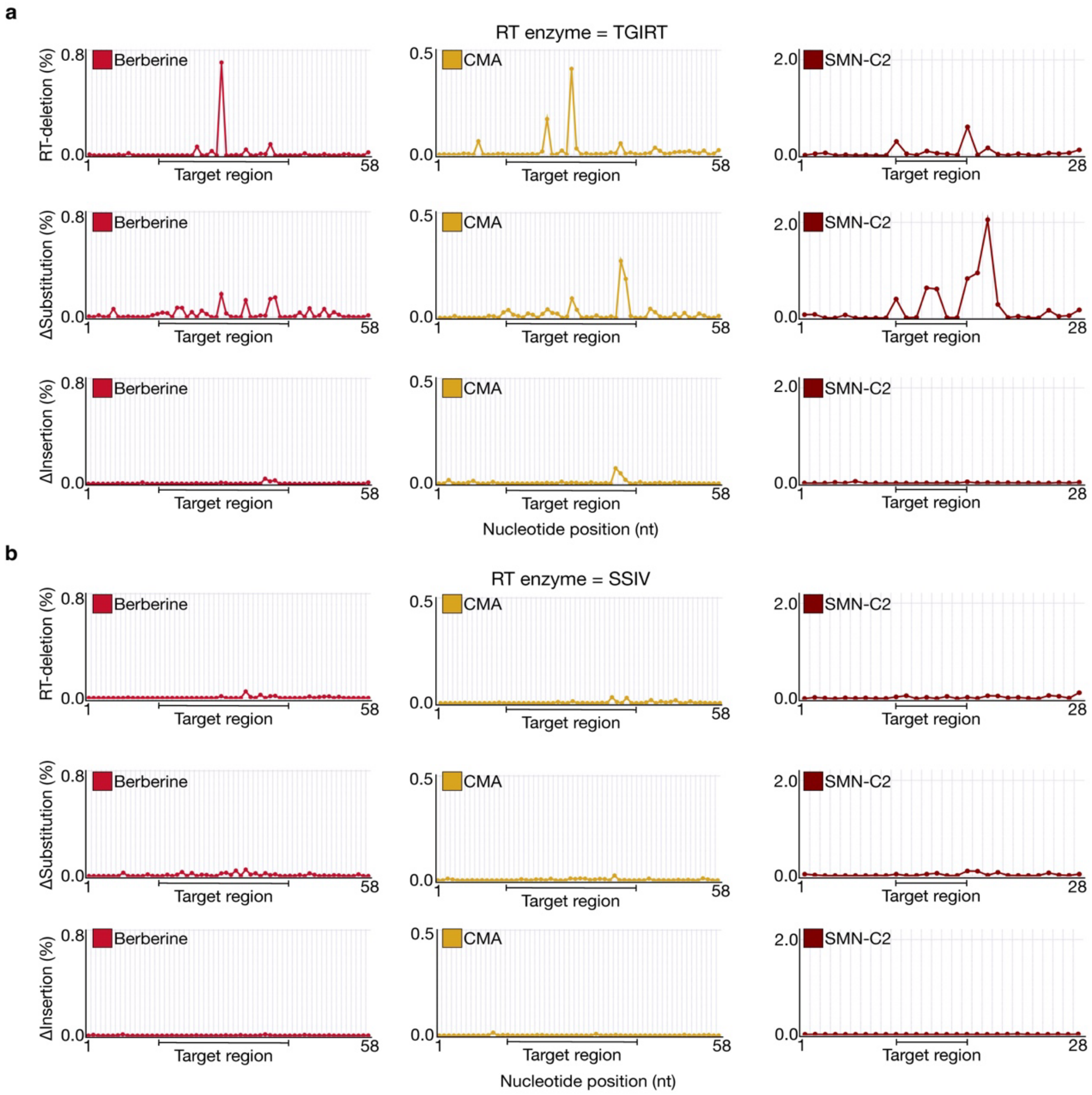

Supplementary Fig. 3 | Modification-derived RT mutation rate with two RT enzymes

The modification-induced mutation percentage (RT-mutation) was determined by subtracting the control RT mutation percentage from the modifier-treated sample. Three types of RT-mutation (substitution, insertion, or deletion) were calculated for each nucleotide. Panel (a) shows results using TGIRT as the reverse transcriptase, while panel (b) shows results using SSIV. G4 HIV-1 LTR was used as the target for berberine and CMA, and Loop GAAGGAAGG loop for SMN-C2. Source data are provided as a Source Data file.

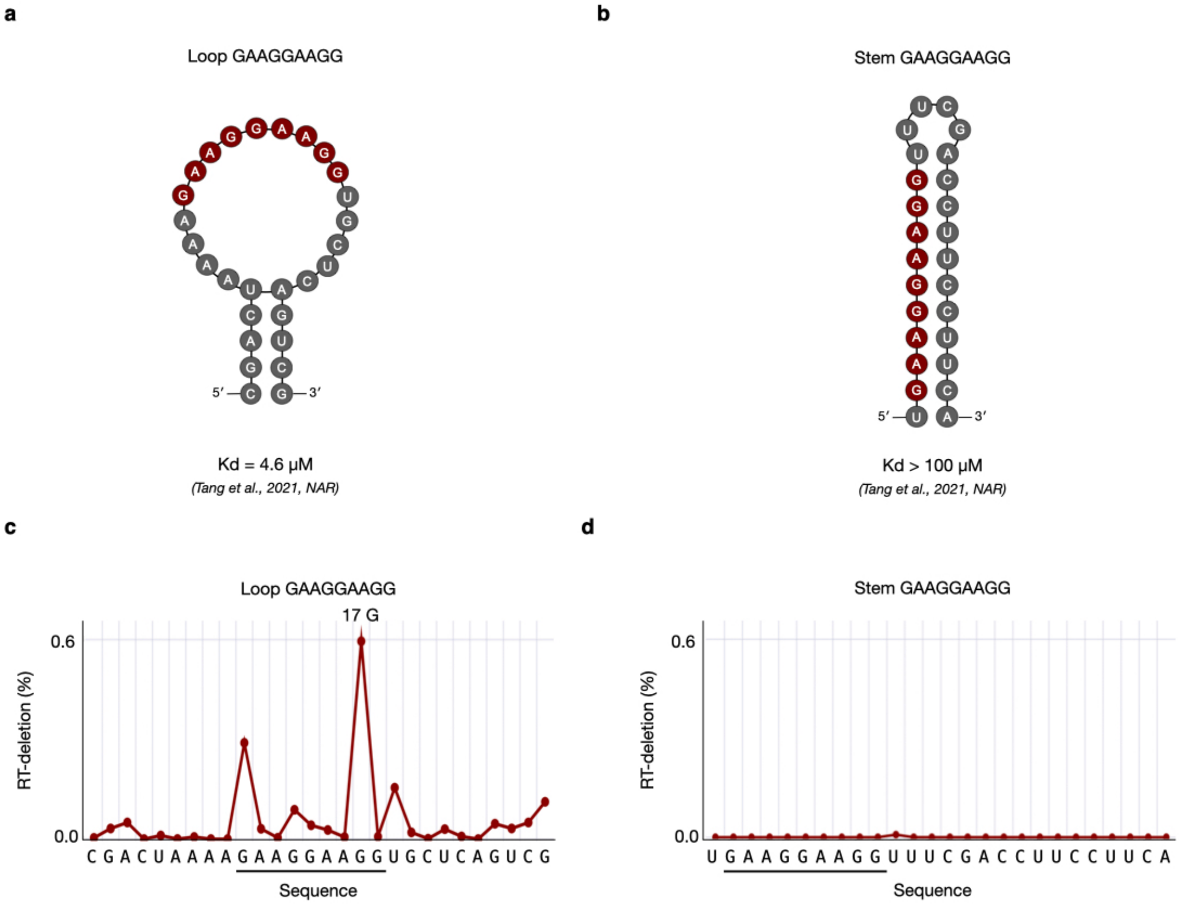

Supplementary Fig. 4 | Detecting loop-specific binding of SMN-C2 by BIVID-MaP

(a–b) SMN-C2 binds to positive control RNA where the target sequence (GAAGGAAGG) is in a loop structure **(a)**. In contrast, the negative control RNA, despite also containing the same target sequence, has it in a stem structure **(b)**. **(c–d)** RT-deletion profile to detect SMN-C2 binding for Loop GAAGGAAGG **(c)** and Stem GAAGGAAGG **(d)**. Target sequence is highlighted by underlining. BIVID-MaP was peformed in two independent experiments (n = 2). Source data are provided as a Source Data file.

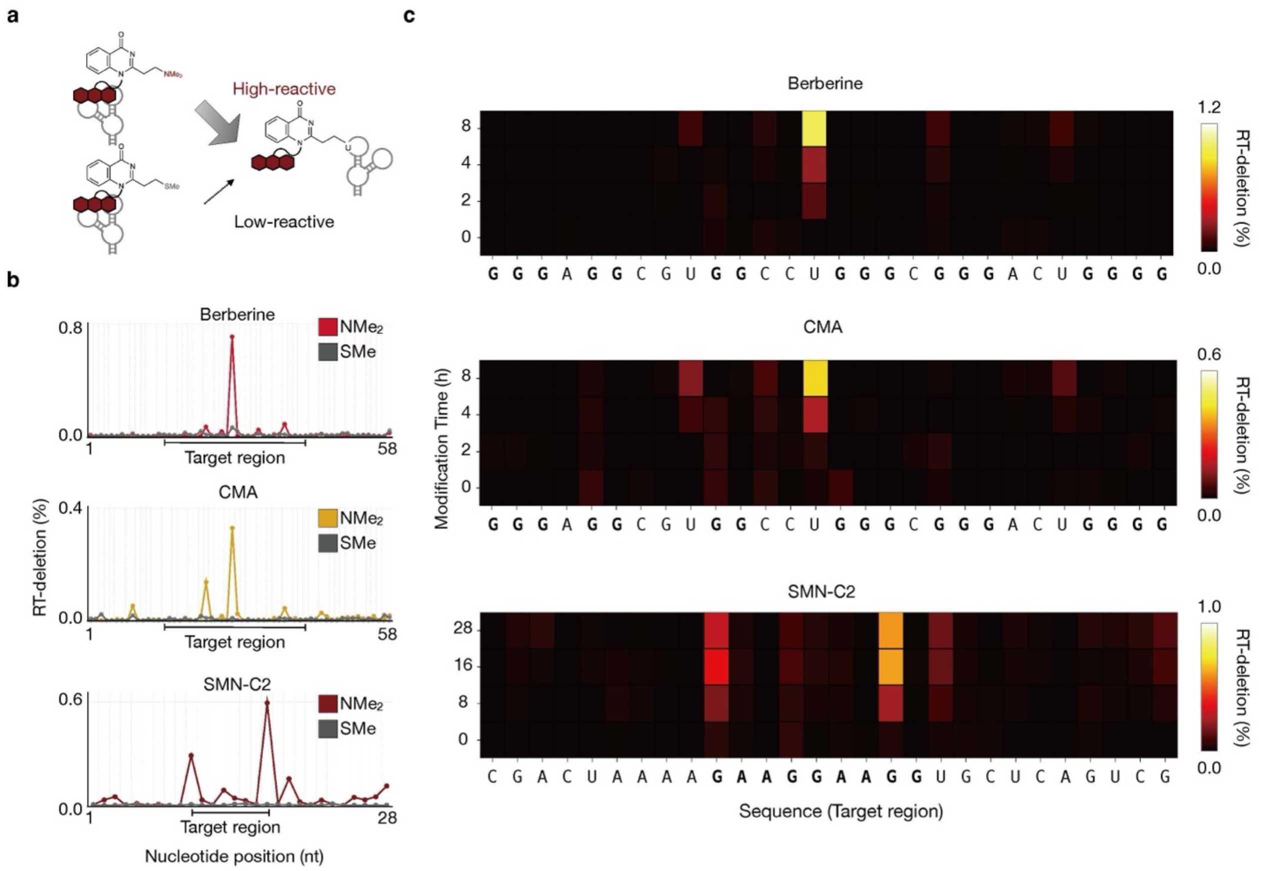

Supplementary Fig. 5 | Deletion detected by BIVID-MaP is derived from binding-dependent covalent modifications.

(a) The schematic diagram of RNA modifications by modifiers with different reactivities. The protective group SMe has a lower modification efficiency than the protective group NMe_2. **(b)** RT-deletion using modifiers with NMe_2 (high reactivity) or SMe (low reactivity). Modification-derived RT deletion frequency (RT-deletion), calculated as the deletion percentage with modifier minus the deletion percentage without modifiers. **(c)** Heat map showing the modification time-dependent increase of RT-deletion. The x-axis shows the sequence of the target RNA structure, and the y-axis shows the modification time, the color scale shows RT-deletion. For berberine and CMA, which target G4 structure, the G-tract-forming guanines are highlighted in bold. For SMN-C2, the target region (GAAGGAAGG) is highlighted in bold. BIVID-MaP was performed in two independent experiments (n = 2). Source data are provided as a Source Data file.

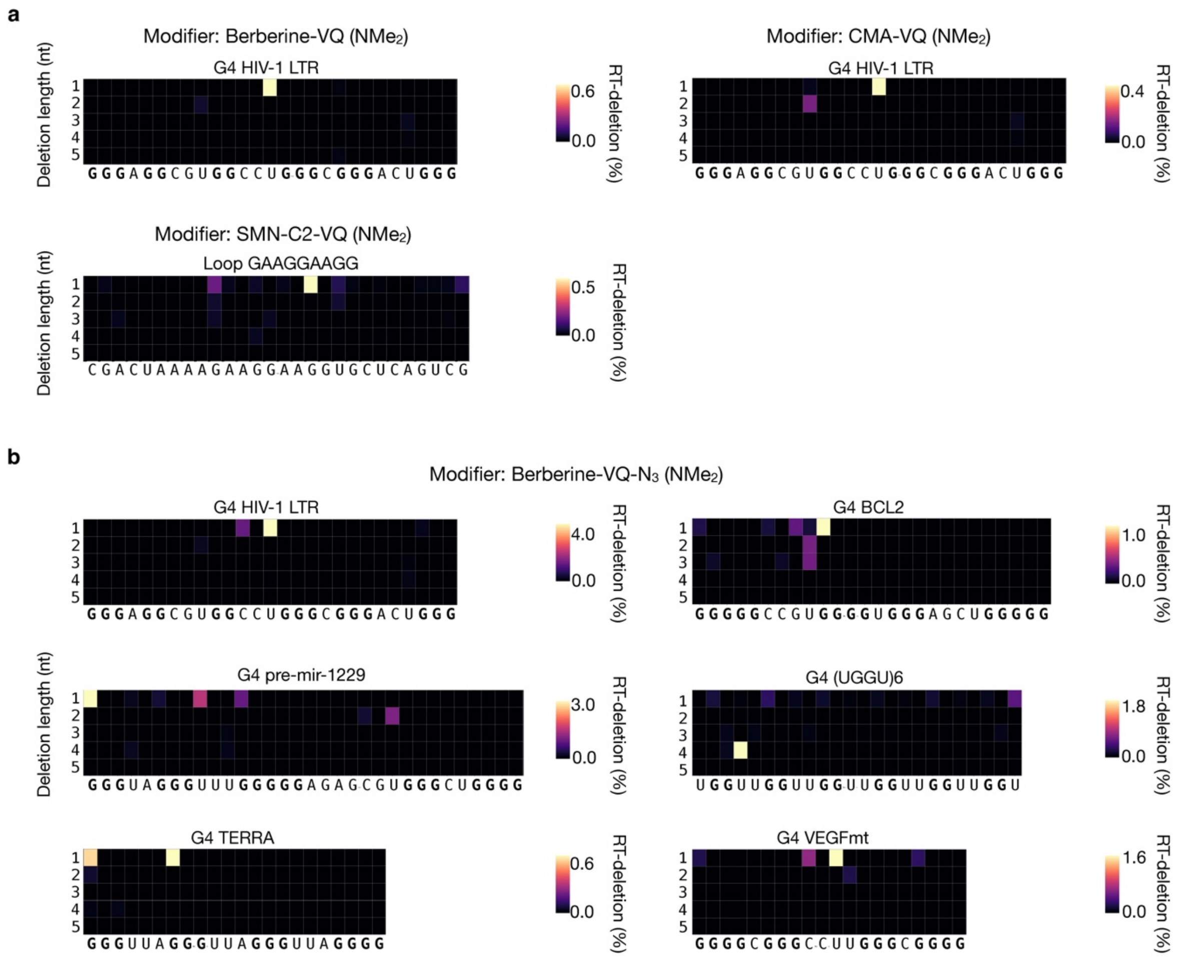

Supplementary Fig. 6 | Deletion length profiles at each nucleotide position

Heat maps of deletion lengths per nucleotide for modifications by **(a)** Berberine-VQ (NMe_2), CMA-VQ (NMe_2), SMN-C2-VQ (NMe_2) or **(b)** Berberine-VQ-N_3 (NMe_2). For modifiers containing an N_3 group, samples reverse transcribed with SSIV were used as controls. For N_3-lacking modifiers, an unmodified sample served as the control. The x-axis shows the sequence of the target RNA structure, and the y-axis shows the length of deleted nucleotides. The color scale shows RT-deletion. For berberine and CMA, which target G4 structure, the G-tract-forming guanines are highlighted in bold. BIVID-MaP was performed in two independent experiments (n = 2). Source data are provided as a Source Data file.

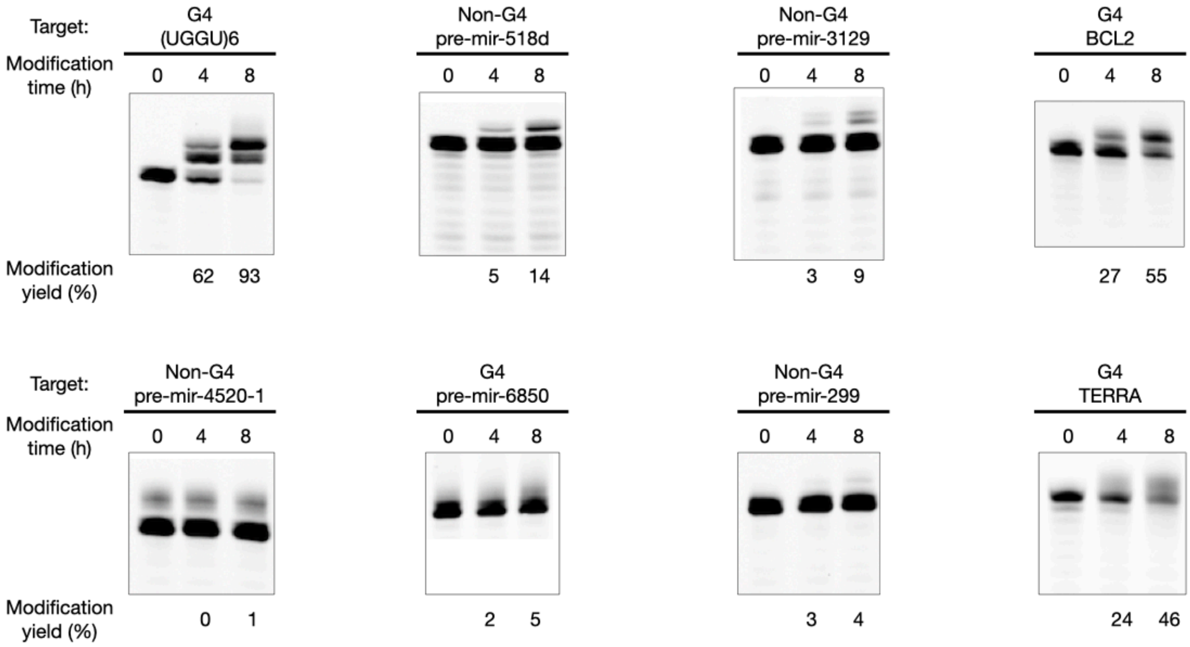

Supplementary Fig. 7 | Gel shift assay showing modifications to RNA structure motifs by Berberine-VQ-N_3 (NMe_2)

Gel images showing the alkylation yield of Berberine-VQ-N_3 (NMe_2) (4 μM) on various folded RNAs (1 μM) after 4 h and 8 h in buffer (20 mM phosphate pH 7.0, 20 mM NaCl, 80 mM KCl). Each experiment was performed in triplicate. From bottom to top, the gel shift bands show unmodified RNA, a 1:1 covalent complex (one modifier per RNA) and a 1:2 covalent complex (two modifiers per RNA) (See Supplementary Note). 5′-FAM labeled RNA was used for gel visualization. For (UGGU)6, a clear band corresponding to the doubly-modified RNA was observed after 8 h. The number of modifications was confirmed by MALDI-TOF MS analysis (**Supplementary Fig. 2**). Unmodified RNA was used as a size marker (See Source Data). Representative data was shown from technical triplicate. Source data are provided as a Source Data file.

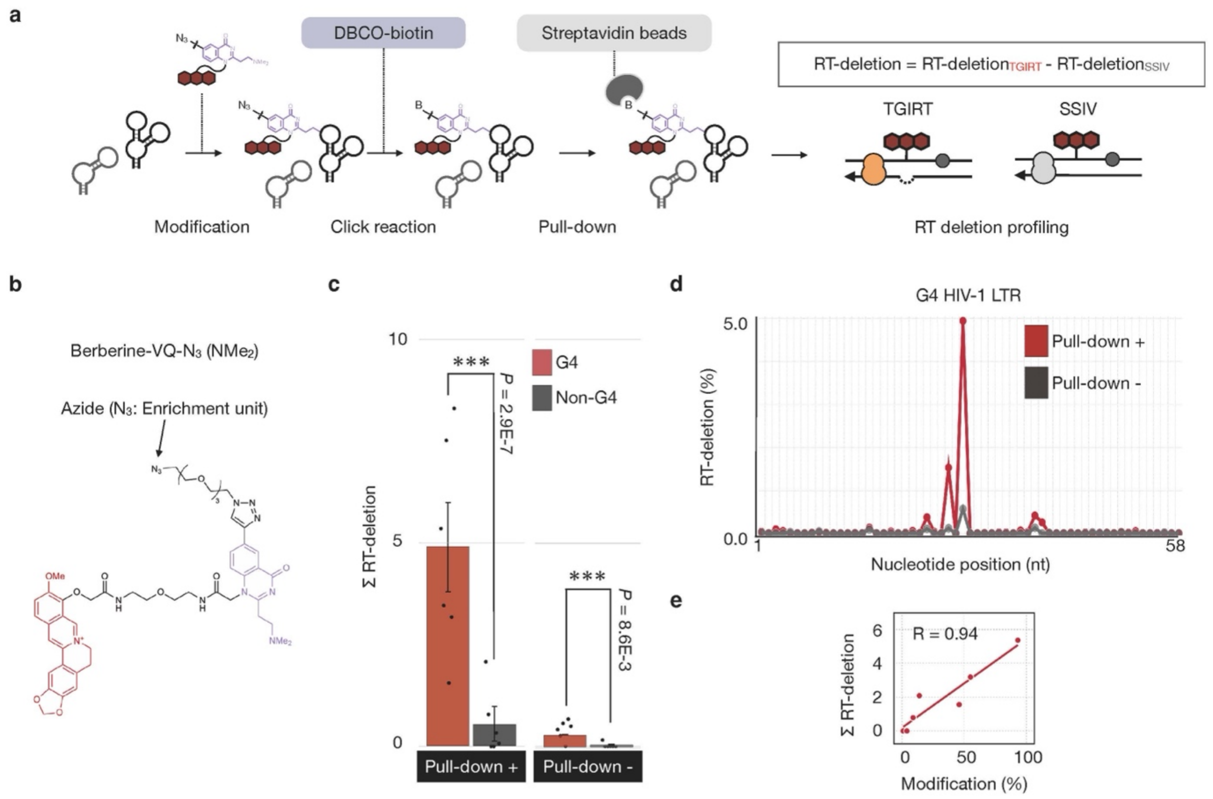

Supplementary Fig. 8 | BIVID-MaP with an enrichment step for modified RNA for large-scale analysis

(a) BIVID-MaP workflow including a pull-down enrichment of modified RNAs. SSIV, which does not induce deletions, served as the control, whereas TGIRT induces deletions at small-molecule binding sites. RT-deletion was defined as the difference in deletion frequency between when TGIRT and SSIV were used, calculated as RT-deletion$_{\text{Berberine-VQ-N3 (NMe2), TGIRT}}$ – RT-deletion$_{\text{Berberine-VQ-N3 (NMe2), SSIV}}$. **(b)** Chemical structures of Berberine-VQ-N_3 (NMe_2), containing an azide for enrichment. **(c)** Σ RT-deletion across target RNAs with enrichment (Pull-down +) and without enrichment (Pull-down -). G4-structured RNAs (red) and non-G4 RNAs (grey) are shown in separate plots. Error bars denote ± SE. The *P*-values were calculated by two-tailed Brunner-Munzel test (***$p < 0.001$; **$p < 0.01$). **(d)** RT-deletion at each nucleotide in the G4 HIV-1 LTR with or without pull-down. **(e)** Correlation between the modification yield and Σ RT-deletion for each target RNA, calculated as the Pearson's correlation coefficient. The modification yield is calculated as the percentage of modified RNA bands out of the total RNA bands in a gel-shift assay at modification time of 8 h. BIVID-MaP was performed in two independent experiments (n = 2). Source data are provided as a Source Data file.

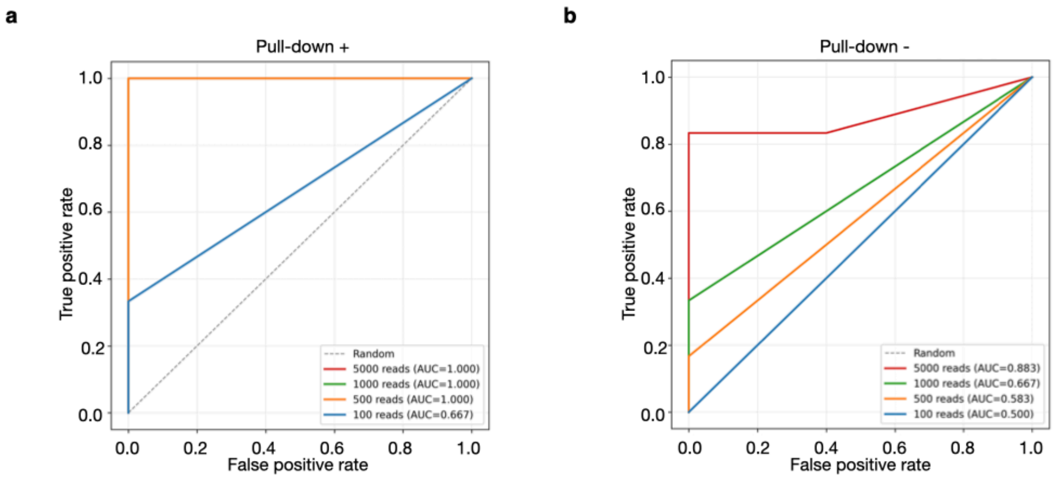

Supplementary Fig. 9 | Adding pull-down step reduced necessary read depth for BIVID-MaP

(a–b) ROC curves of averaged Σ RT-deletion scores distinguishing berberine binding to highly reactive G4 structures from binding to less reactive non-G4 controls. BIVID-MaP performed with **(a)** or without (**b**) pull-down. Curves are overlaid for 100 (blue), 500 (orange), 1000 (green), and 5000 (red) read subsets. Average scores from three independent downsampling replicates are used. Dashed diagonal denotes random classification. Source data are provided as a Source Data file.

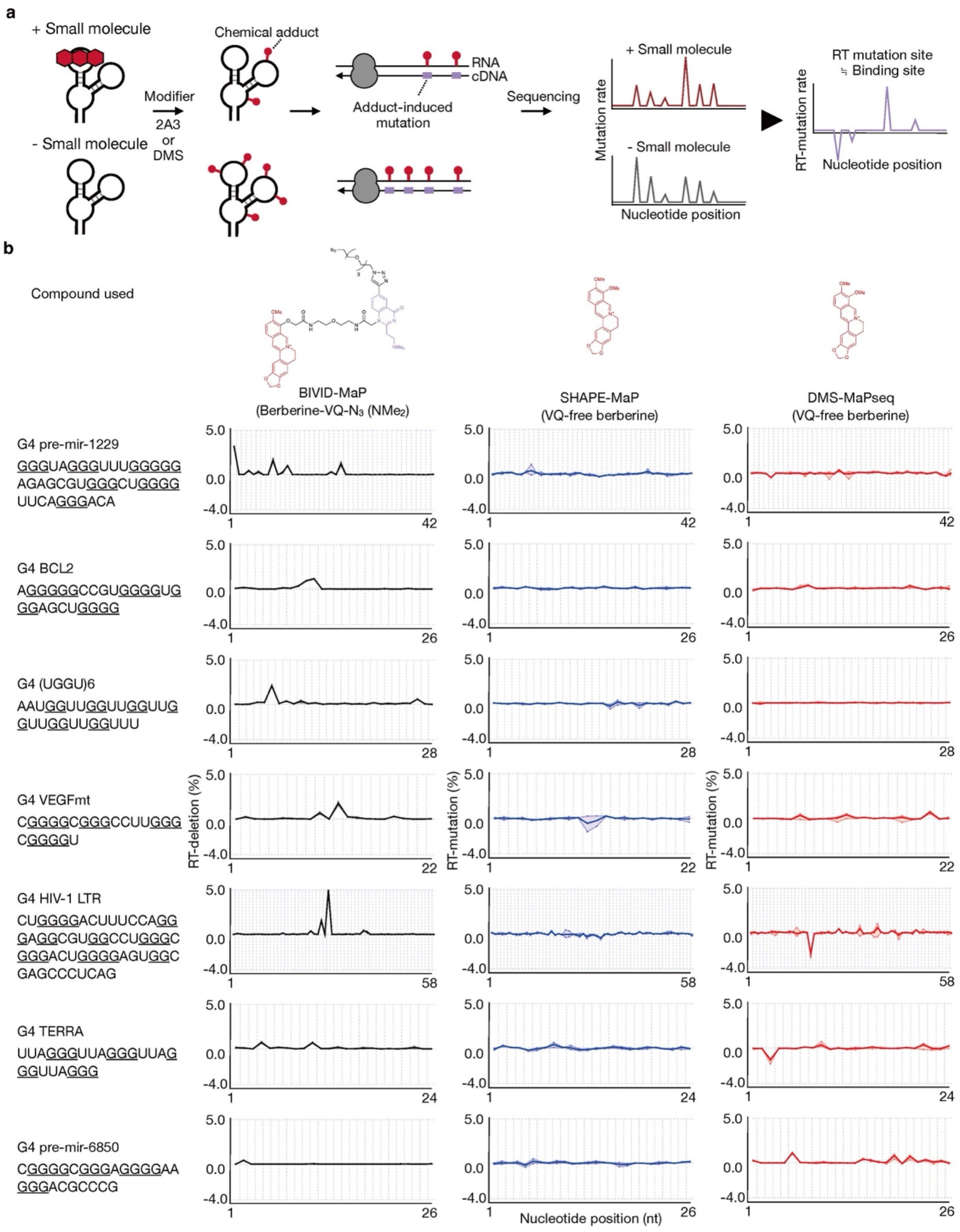
a
+ Small molecule
- Small molecule
Modifier
2A3
or
DMS
Chemical adduct
RNA
cDNA
Adduct-induced
mutation
Sequencing
+ Small molecule
- Small molecule
Mutation rate
Nucleotide position
RT mutation site
≒ Binding site
RT-mutation rate
Nucleotide position
b
Compound used
BIVID-MaP
(Berberine-VQ-N3 (NMe2))
SHAPE-MaP
(VQ-free berberine)
DMS-MaPseq
(VQ-free berberine)
G4 pre-mir-1229
GGGUAGGGUUUGGGGG
AGAGCGUGGGCUGGGG
UUCAGGGACA
G4 BCL2
AGGGGGCCGUGGGGUG
GGAGCUGGGG
G4 (UGGU)6
AAUGGUUGGUUGGUUG
GUUGGUUGGUUU
G4 VEGFmt
CGGGGCGGGCCUUGGG
CGGGGU
G4 HIV-1 LTR
CUGGGGACUUUCCAGG
GAGGCGUGGCCUGGGC
GGGACUGGGGAGUGGC
GAGCCCUCAG
G4 TERRA
UUAGGGUUAGGGUUAG
GGUUAGGG
G4 pre-mir-6850
CGGGGCGGGAGGGGAA
GGGACGCCCG
RT-deletion (%)
RT-mutation (%)
RT-mutation (%)
Nucleotide position (nt)

Supplementary Fig. 10 | Comparison of BIVID-MaP, SHAPE-MaP and DMS-MaPseq

(a) The overview of SHAPE-MaP or DMS-MaPseq to detect RNA-small molecule interactions. Ligand binding to target RNA structures changes the modification of 2A3 or DMS. RNA-small molecule interactions can be detected by RT mutation frequency (RT-mutation), calculated as the mutation percentage with ligand and modifier minus the mutation percentage with modifier alone. **(b)** Comparison of BIVID-MaP, SHAPE-MaP, and DMS-MaPseq to detect the RNA structure-berberine interactions. Target RNA library containing G4 structures was incubated with Berberine-VQ-N_3 (NMe_2) in BIVID-MaP, with VQ-free berberine in SHAPE-MaP and DMS-MaPseq. Y-axis shows the RT-deletion in BIVID-MaP, the RT-mutation in SHAPE-MaP or DMS-MaPseq. Shaded bands indicate the range between the two replicates. The G-tract-forming guanines are highlighted by underlining. All MaP experiments were performed twice independently (n = 2). Source data are provided as a Source Data file.

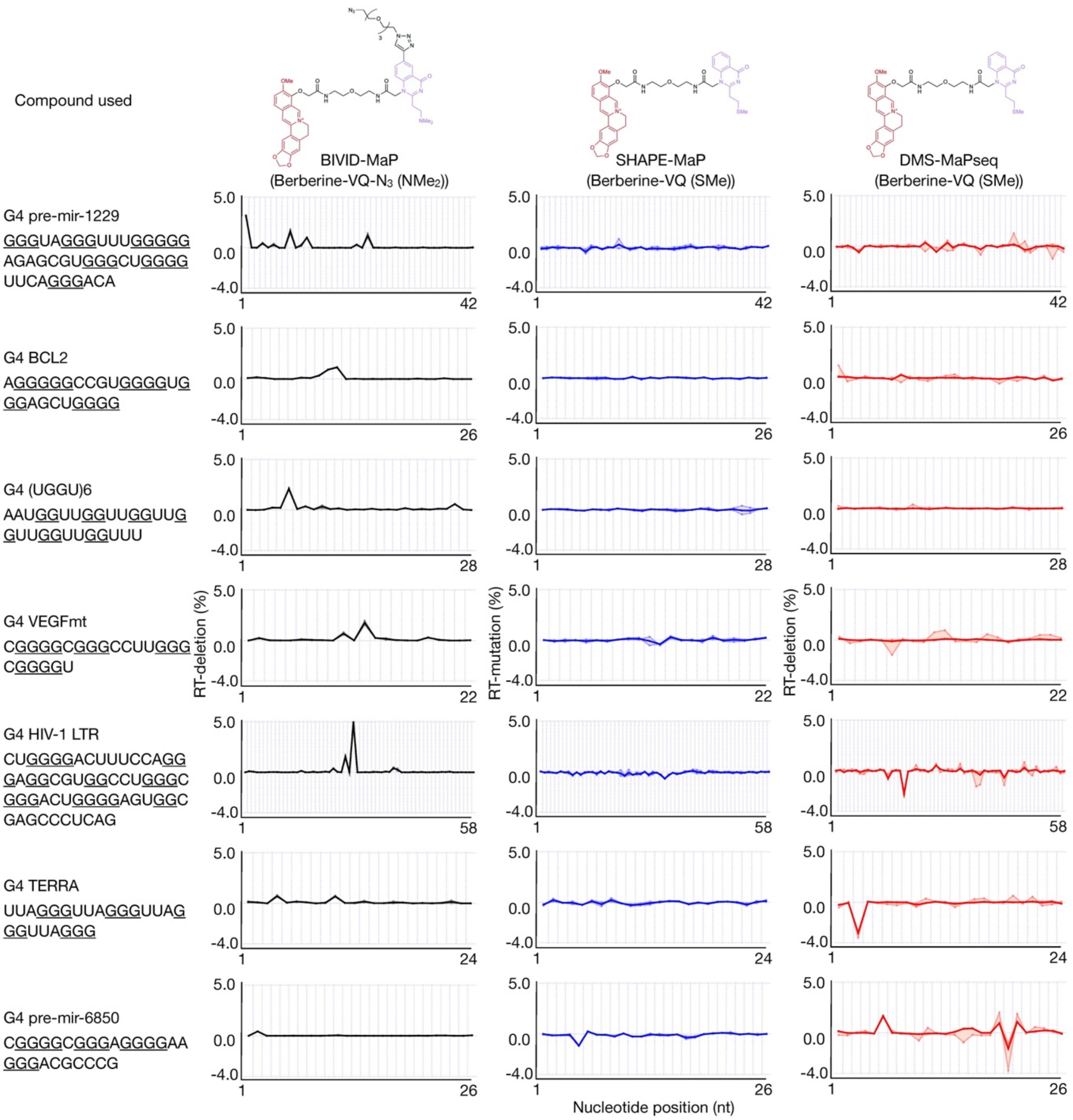

Supplementary Fig. 11 | Comparison of BIVID-MaP, SHAPE-MaP, and DMS-MaPseq using VQ-conjugated berberine

Comparison of BIVID-MaP, SHAPE-MaP, and DMS-MaPseq for detecting interactions between RNA structures and VQ-conjugated berberine. In addition to VQ-free berberine, we also performed SHAPE-MaP and DMS-MaPseq using Berberine-VQ (SMe) which has low modification reactivity, to account for potential effects of the VQ moiety itself. Target RNA library containing G4 structures was incubated with Berberine-VQ-N_3 (NMe_2) in BIVID-MaP, with Berberine-VQ (SMe) in SHAPE-MaP and DMS-MaPseq. Y-axis shows the RT-deletion in BIVID-MaP, RT-mutation in SHAPE-MaP or DMS-MaPseq. Shaded bands indicate the range between the two replicates. The G-tract-forming guanines are highlighted by underlining. All MaP experiments were performed twice independently (n = 2). Source data are provided as a Source Data file.

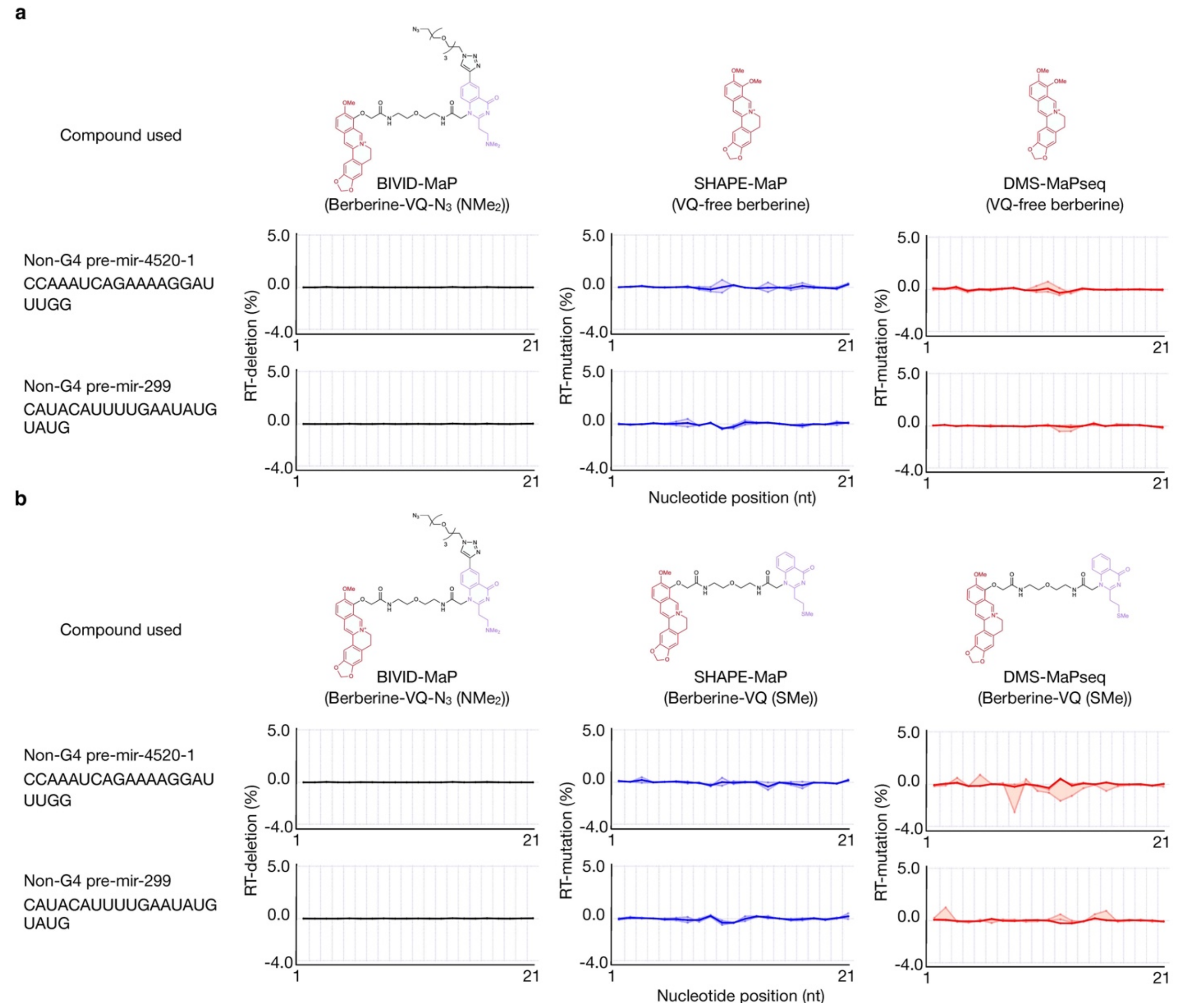

Supplementary Fig. 12 | Reactivities for negative control RNAs in BIVID-MaP, SHAPE-MaP and DMS-MaPseq

Comparison of the BIVID-MaP, SHAPE-MaP and DMS-MaPseq to detect the non-G4 structure-berberine interactions. In addition to VQ-free berberine, we also performed SHAPE-MaP and DMS-MaPseq using Berberine-VQ (SMe) which has low modification reactivity, to account for potential effects of the VQ moiety itself. Non-G4 structure is used as less reactive controls. Berberine-VQ-N_3 (NMe_2) was used in BIVID-MaP. For SHAPE-MaP and DMS-MaPseq, VQ-free berberine was used in panel (a) while Berberine-VQ (SMe) was used in panel (b). Y-axis shows the RT-deletion in BIVID-MaP, the RT-mutation in SHAPE-MaP or DMS-MaPseq. Shaded bands indicate the range between the two replicates. All MaP experiments were performed twice independently (n = 2). Source data are provided as a Source Data file.

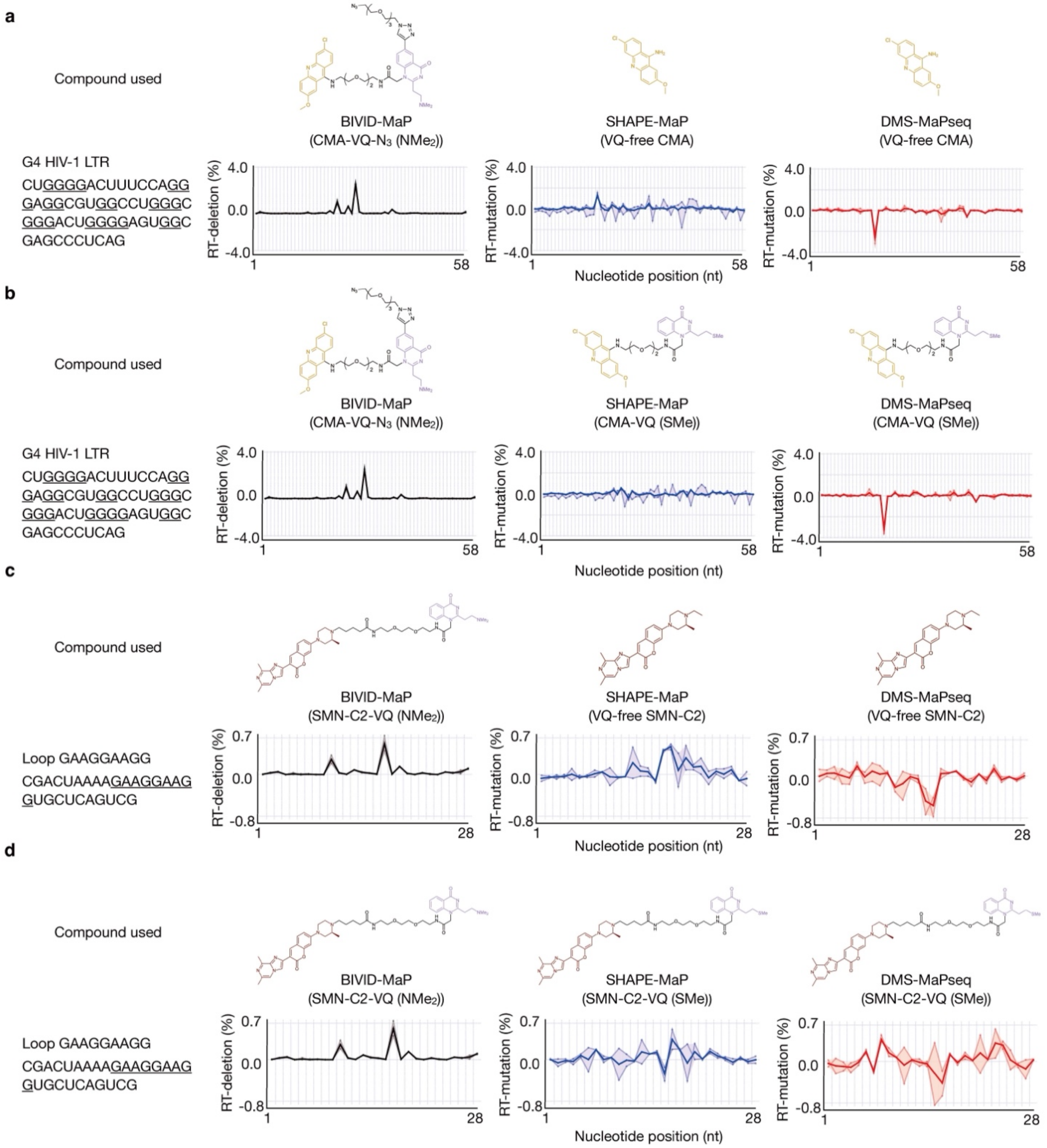
a
Compound used
BIVID-MaP
(CMA-VQ-N3 (NMe2))
SHAPE-MaP
(VQ-free CMA)
DMS-MaPseq
(VQ-free CMA)
G4 HIV-1 LTR
CUGGGGACUUUCCAGG
GAGGCGUGGCCUGGGC
GGGACUGGGGAGUGGC
GAGCCCUCAG
RT-deletion (%)
RT-mutation (%)
4.0
0.0
-4.0
1
58
Nucleotide position (nt)
b
Compound used
BIVID-MaP
(CMA-VQ-N3 (NMe2))
SHAPE-MaP
(CMA-VQ (SMe))
DMS-MaPseq
(CMA-VQ (SMe))
G4 HIV-1 LTR
CUGGGGACUUUCCAGG
GAGGCGUGGCCUGGGC
GGGACUGGGGAGUGGC
GAGCCCUCAG
RT-deletion (%)
RT-mutation (%)
Nucleotide position (nt)
c
Compound used
BIVID-MaP
(SMN-C2-VQ (NMe2))
SHAPE-MaP
(VQ-free SMN-C2)
DMS-MaPseq
(VQ-free SMN-C2)
Loop GAAGGAAGG
CGACUAAAAGAAGGAAG
GUGCUCAGUCG
RT-deletion (%)
RT-mutation (%)
0.7
0.0
-0.8
1
28
Nucleotide position (nt)
d
Compound used
BIVID-MaP
(SMN-C2-VQ (NMe2))
SHAPE-MaP
(SMN-C2-VQ (SMe))
DMS-MaPseq
(SMN-C2-VQ (SMe))
Loop GAAGGAAGG
CGACUAAAAGAAGGAAG
GUGCUCAGUCG
RT-deletion (%)
RT-mutation (%)
Nucleotide position (nt)

Supplementary Fig. 13 | Comparison of BIVID-MaP, SHAPE-MaP, and DMS-MaPseq to detect multiple RNA structure-small molecule interactions

(a-d) Comparison of the BIVID-MaP, SHAPE-MaP, and DMS-MaPseq to detect the G4 structure-CMA interactions. Source data are provided as a Source Data file. **(a–b)**, detect the loop structure-SMN-C2 interactions **(c-d)**. Y-axis shows the RT-deletion in BIVID-MaP, the RT-mutation in SHAPE-MaP or DMS-MaPseq. Shaded bands indicate the range between the two replicates. For SHAPE-MaP and DMS-MaPseq, VQ-free compounds are used in **(a)**, **(c)** while VQ (SMe) conjugates are used in **(b)**, **(d)**. For BIVID-MaP, CMA-VQ-N_3 (NMe_2) or SMN-C2-VQ (NMe_2) was used. The G-tract-forming guanines are highlighted by underlining in G4 HIV-1 LTR. The pull-down step was added when using CMA-VQ-N_3 (NMe_2), but not when using SMN-C2-VQ (NMe_2). All MaP experiments were performed twice independently (n = 2). Source data are provided as a Source Data file.

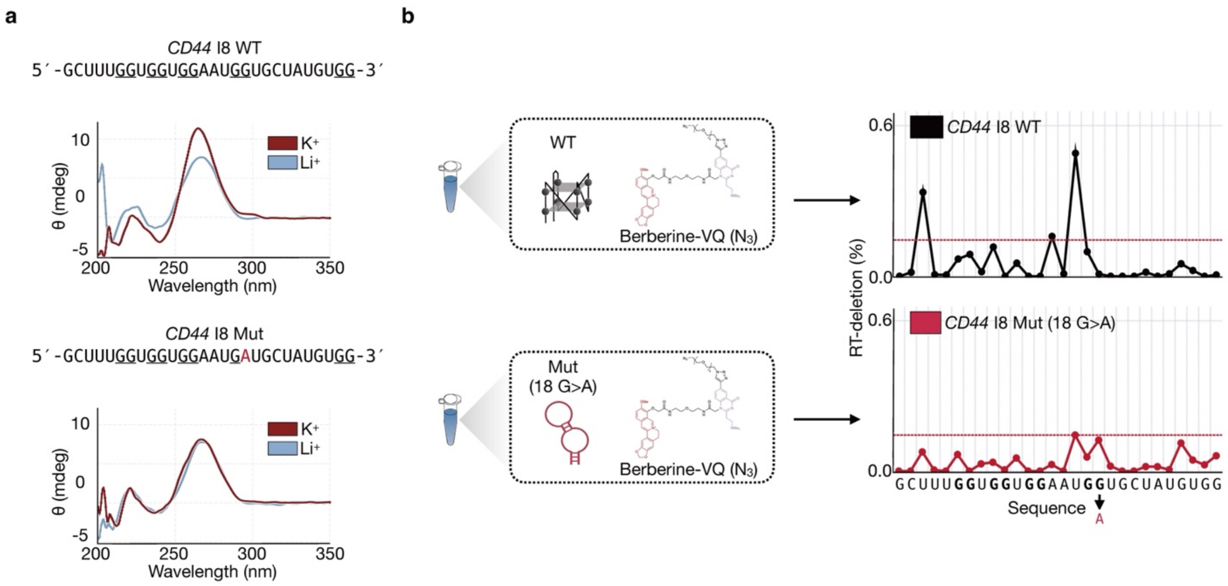

Supplementary Fig. 14 | G4 structure-specific interaction with berberine modulated by G to A mutation

(a) Circular dichroism (CD) spectra of *CD44* I8 WT or *CD44* I8 Mut RNA oligonucleotides in buffers containing either K^+ or Li^+, confirming G4 formation and its disruption by mutation. The G-tract-forming guanines are highlighted by underlining. **(b)** RT-deletion profile when WT and mutant RNAs were modified with Berberine-VQ-N_3 (NMe_2) separately. The grey or red curves show RT-deletion for WT or mutant sequences, respectively. The red dashed line shows the highest RT-deletion in the mutant. The G-tract-forming guanines are highlighted in bold. BIVID-MaP was performed in two independent experiments (n = 2). Source data are provided as a Source Data file.

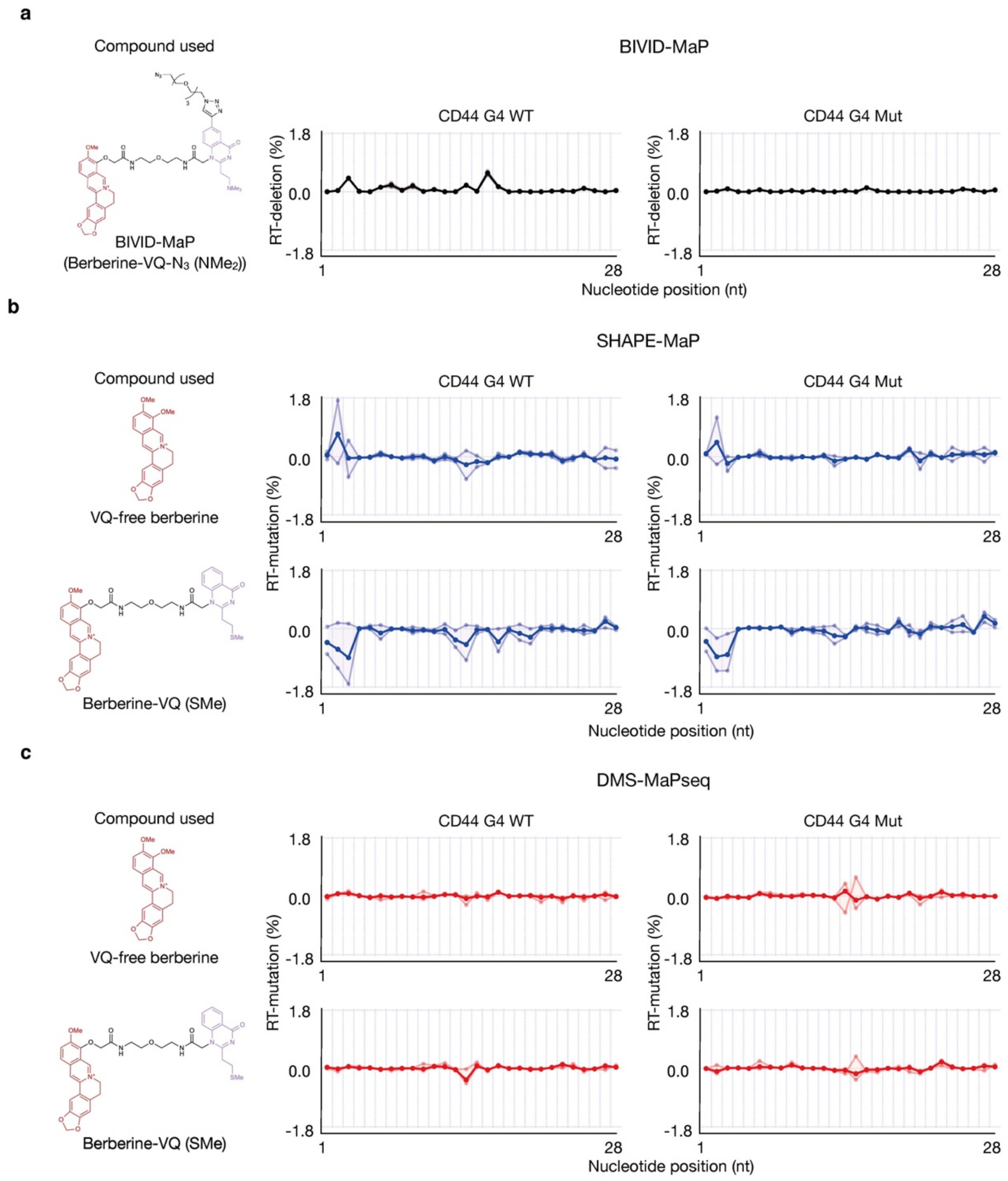

Supplementary Fig. 15 | SNV-specific RNA-small molecule interactions were not detected by either SHAPE-MaP or DMS-MaPseq

(a-c) Detection of G4 structure-specific interactions between berberine and CD44 G4 variants by BIVID-MaP **(a)**, SHAPE-MaP **(b),** and DMS-MaPseq **(c)**. VQ-free berberine or Berberine-VQ (SMe) was incubated with a mixture containing both CD44 G4 WT and CD44 G4 Mut. Sequencing reads were separated for each variant, and RT-deletion or RT-mutation was calculated for each sequence. Berberine-VQ-N_3 (NMe_2) was used in BIVID-MaP. For SHAPE-MaP and DMS-MaPseq, VQ-free berberine or Berberine-VQ (SMe) was used. The y-axis shows the RT-deletion in BIVID-MaP and the RT-mutation in SHAPE-MaP or DMS-MaPseq. Shaded bands indicate the range between the two replicates. All MaP experiments were performed twice independently (n = 2). Source data are provided as a Source Data file.

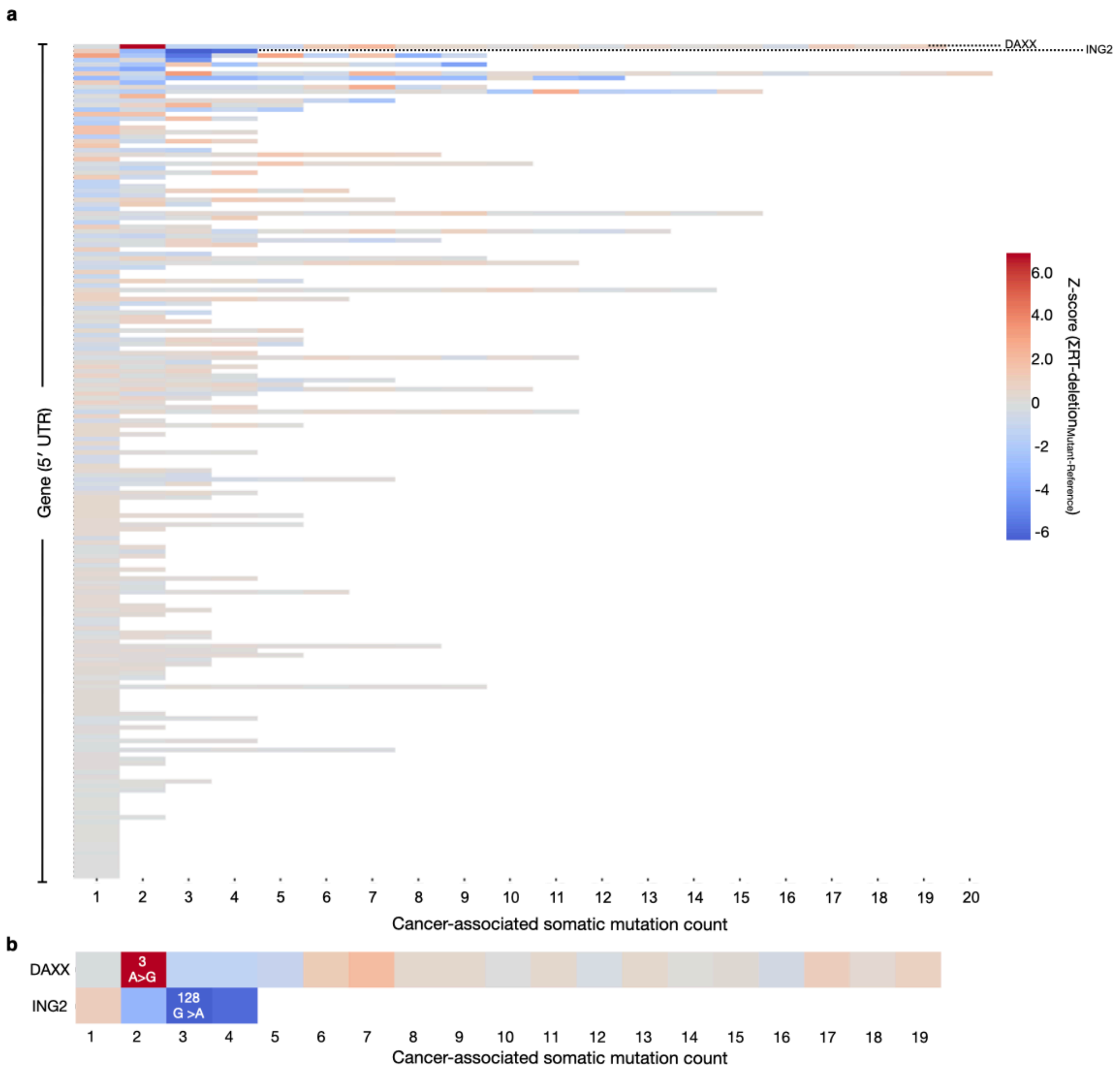

Supplementary Fig. 16 | Heat map of RT-deletion changes in 5′ UTRs caused by somatic mutations

(a) Heat map comparing RT-deletion between reference and mutant sequences for all genes in the 5′ UTR somatic mutation library. Each column is a somatic mutation indexed in increasing order from the 5′ UTR, and each row is a gene ranked by the highest Σ RT-deletion$_{\text{Mutant -Reference}}$. The color scale represents the change in reactivity of Z-score (Σ RT-deletion$_{\text{Mutant -Reference}}$). The *DAXX* and *ING2* rows are highlighted. **(b)** Expanded view of the heat map in (a) for *DAXX* and *ING2*. The mutation with the highest |Σ RT-deletion$_{\text{Mutant -Reference}}$| in each gene is highlighted. BIVID-MaP was performed in two independent experiments (n = 2). Source data are provided as a Source Data file.

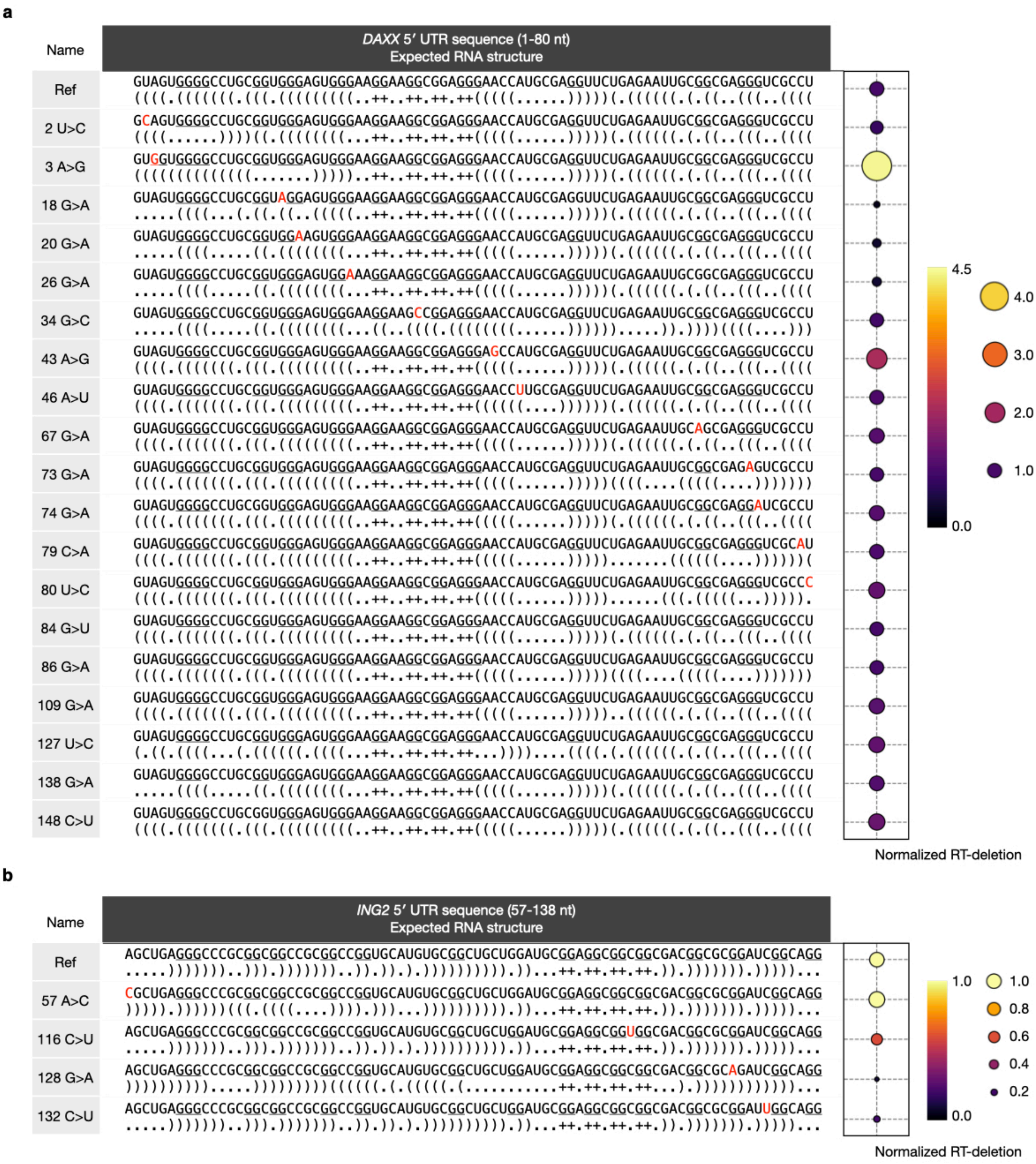

Supplementary Fig. 17 | Normalized RT-deletion of all analyzed variants in *DAXX* or *ING2*

(a–b) The most reactive nucleotide in the reference sequences was used to calculate the RT-deletion. The mutant nucleotide is highlighted in red. (+) indicates guanine residues forming a G-quadruplex in the predicted RNA secondary structure using RNAfold. Circle size and color scale indicate normalized RT-deletion. The guanines forming G-tracts are highlighted by underlining. BIVID-MaP was performed in two independent experiments (n = 2). Source data are provided as a Source Data file.

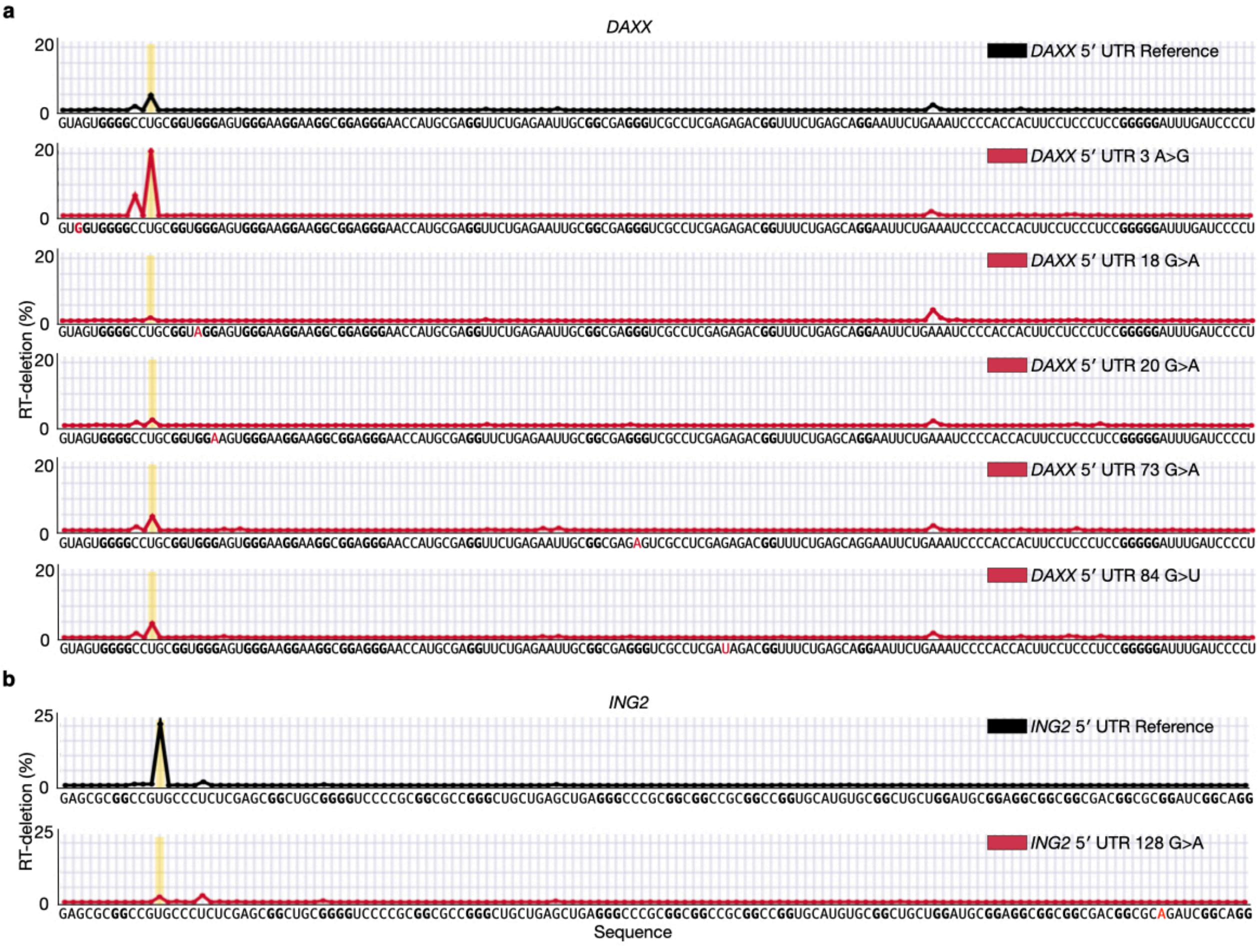

Supplementary Fig. 18 | RT-deletion profiles showing variant-specific interactions between 5′ UTRs and berberine.

(a–b) Reference and mutant sequences are shown in black and red, respectively. The mutant base is in red on the x-axis. The base (12U) with the highest RT-deletion in each reference sequence is highlighted in yellow. The G-tract-forming guanines are highlighted in bold. BIVID-MaP was performed in two independent experiments (n = 2). Source data are provided as a Source Data file.

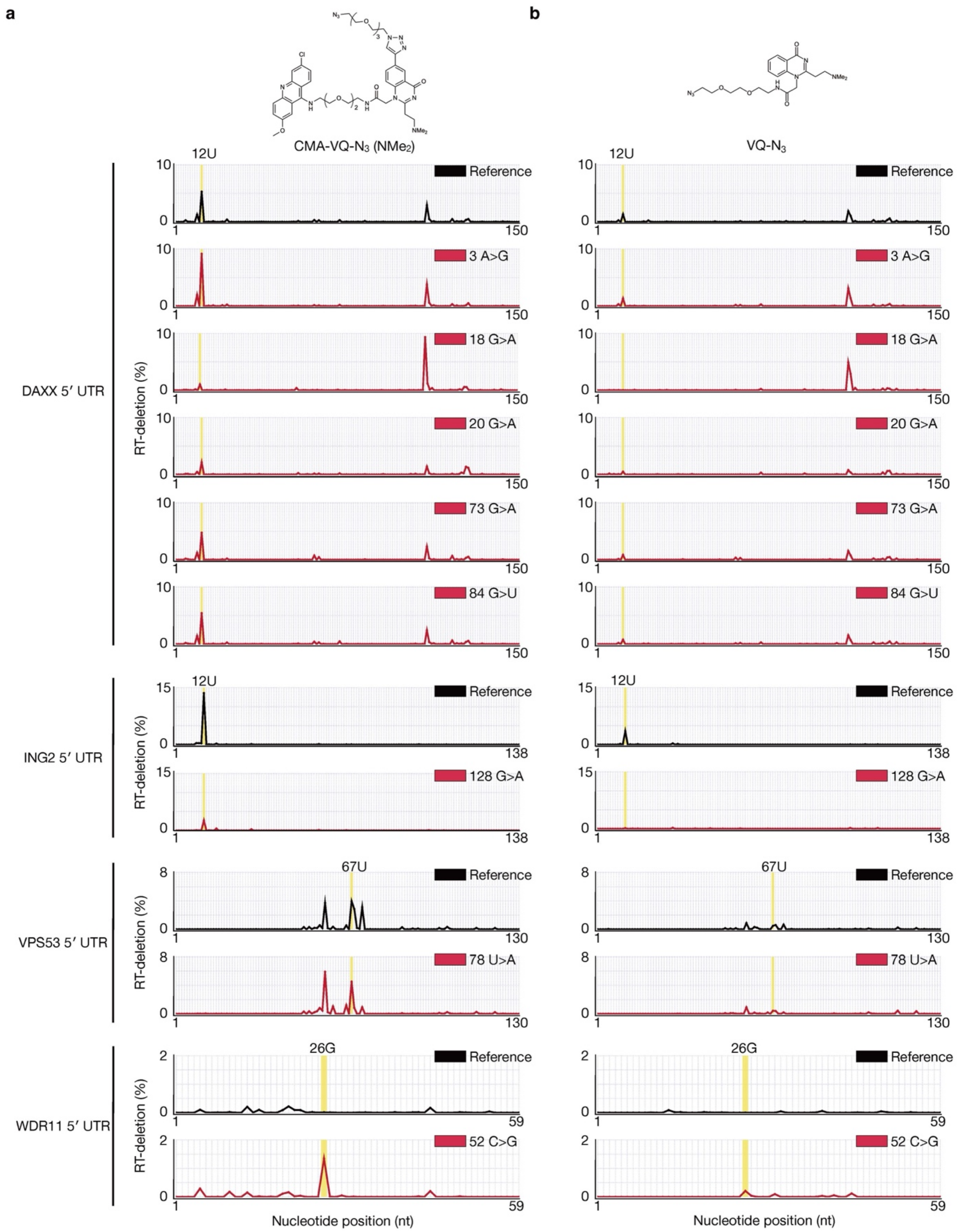

Supplementary Fig. 19 | RT-deletion profiles of 5′ UTR variants with CMA-VQ-N_3 (NMe_2) or VQ-N_3

RT-deletion profiles with CMA-VQ-N_3 (NMe_2) **(a)** or VQ-N_3 **(b)**. Reference and mutant sequences are shown in black and red, respectively. Highlighted in yellow is the base that exhibits the strongest RT-deletion in each reference sequence when observed with Berberine-VQ-N_3 (NMe_2). BIVID-MaP was performed in two independent experiments (n = 2). Source data are provided as a Source Data file.

a

Gene	Mutation	5′UTR length (nt)	Window start (nt)	Window end (nt)	G4NN	G4H	cGcC
DAXX	-	150	1	60	0.82	0.98	6.50
	3 A>G	150	1	60	0.84	1.03	6.75
	18 G>A	150	1	60	0.78	0.90	6.00
	20 G>A	150	1	60	0.79	0.90	6.00
ING2	-	138	81	138	0.56	0.62	3.06
	128 G>A	138	81	138	0.44	0.57	2.88
VPS53	-	130	61	120	0.93	1.35	7.35
	78 U>A	130	61	120	0.93	1.35	7.35
WDR11	-	59	1	59	0.06	0.14	1.25
	52 C>G	59	1	59	0.10	0.27	1.51

b

DAXX 5′UTR (1 … 60 … 150)

GUAGUGGGGCCUGCGGUGGGAGUGGGAAGGAAGGCGGAGGGAACCAUGCGAGGUUCUGAGAAUUGCGGCGAGGGUCGCCUCG
AGAGACGGUUUCUGAGCAGGAAUUCUGAAAUCCCCACCACUUCCUCCCUCCGGGGGAUUUGAUCCCCU

ING2 5′UTR (1 … 81 … 138)

GAGCGCGGCCGUGCCCUCUCGAGCGGCUGCGGGGUCCCCGCGGCGCCGGGCUGCUGAGCUGAGGGCCCGCGGCGGCCGCGGC
CGGUGCAUGUGCGGCUGCUGGAUGCGGAGGCGGCGGCGACGGCGCGGAUCGGCAGG

VPS53 5′UTR (1 … 61 … 120 … 130)

GACAGAGCGGCUGACGGAGCCGGGCUCACCAGGUCGCUGCCGCGAGGGAGUUGCUGUGCUGGGGCCUGGGUGGCGGCUGGAG
GCCUGAGUUGGGCUCGCGGCGGGGGUCGGCAGGGGGCCGGGUGGCGGA

*WDR11*5′UTR (1 … 59)

ACAGUGUCCGCCGCUUCCUGGUUGCGGGUCAGCGCCCAGGUCCUGGGCUGGCCGCCGGG

Supplementary Fig. 20 | G4 structure prediction by multiple software tools for the 5′ UTR variants

(a) Each 5′ UTR sequence (reference and single-nucleotide mutants) was scored with the G4RNA Screener pipeline using 60-nt sliding windows (step size 10 nt). For each 5′ UTR variant, the neural-network score (G4NN), G4Hunter score (G4H), and cGcC index were calculated. Reported values are the maximum across all windows, with the window boundaries defined by the G4NN peak. Scores exceeding the common threshold (G4NN: 0.5, G4H: 0.9, cGcC: 4.5) are highlighted in orange. **(b)** The sequences of target 5′ UTRs. The highest G4 structure-forming probability region defined by G4NN is highlighted in red. Within this region, G-tract-forming guanines are shown in bold.

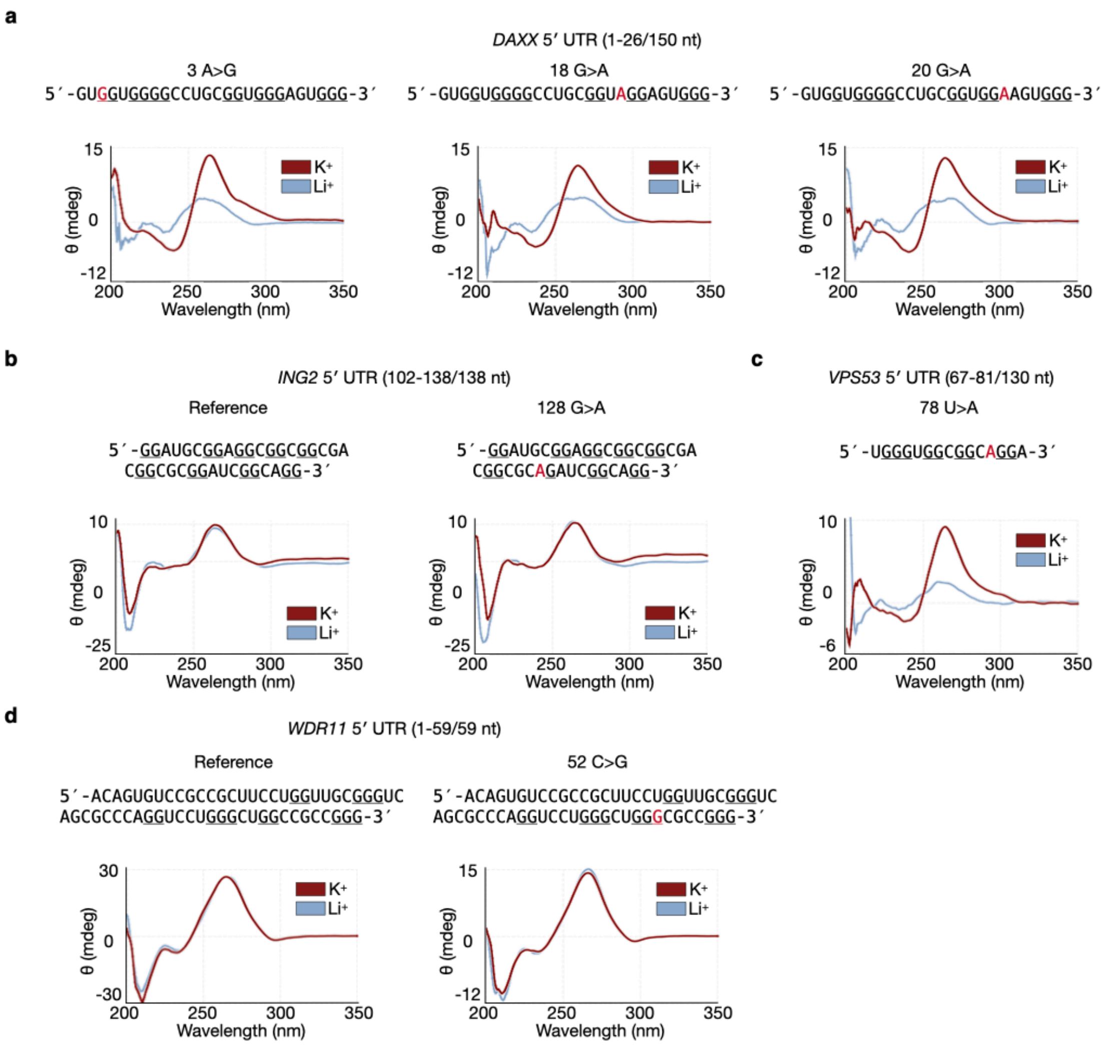

Supplementary Fig. 21 | Validation of RNA structures by CD spectrum analysis

(a-d) CD spectrum analysis in the presence of 100 mM KCl or LiCl. The spectra of RNA G4 structure show a negative peak at 241 nm and a positive peak at 265 nm. These G4-specific signatures were diminished in the presence of lithium ion because the probability of G4 structure is higher in the presence of potassium ions than of smaller cationic ions. **(a)** The 1-26 nt region of *DAXX* 5′ UTR, **(b)** The 102-138 nt region of *ING2* 5′ UTR, **(c)** The 67-81 nt region of *VPS53* 5′ UTR, **(d)** The full length of *WDR11* 5′ UTR. Source data are provided as a Source Data file.

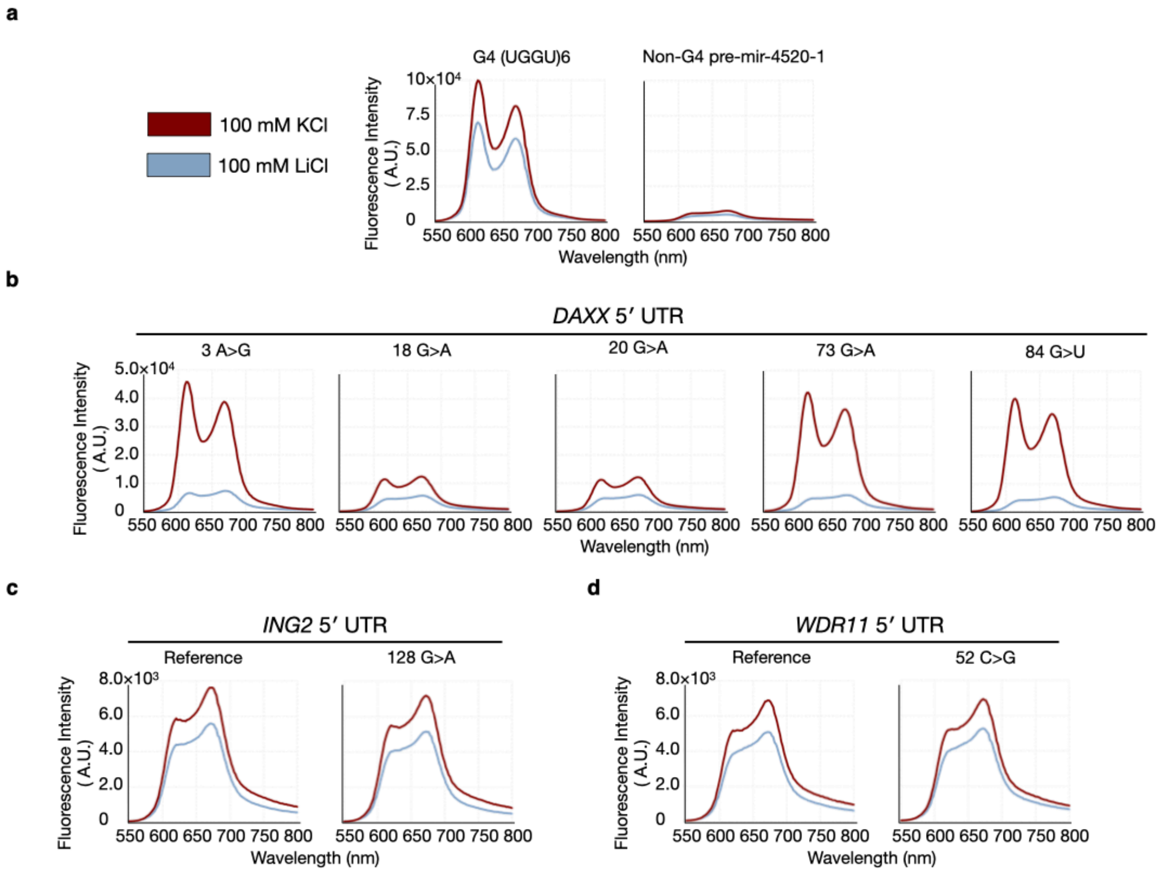

Supplementary Fig. 22 | Fluorescence emission of NMM binding to RNA in the presence of K^+ or Li^+ ions

(a–d) G4 structural formation was analyzed with full-length 5′ UTR by using N-methyl mesoporphyrin IX (NMM), which senses G4 by enhanced fluorescence (λex=399 and λem=550-800nm). Weak fluorescence was observed under Li^+, which was substantially enhanced when substituted with K^+, supporting the formation of RNA G4 that allows recognition of NMM and enhances its fluorescence. **(a)** G4 control (UGGU)6 and Non-G4 control pre-mir-4520-1, **(b)** *DAXX* 5′ UTR, **(c)** *ING2* 5′ UTR, **(d)** *WDR11* 5′ UTR. NMM fluorescence assays were peformed in three independent experiments (n = 3). Source data are provided as a Source Data file.

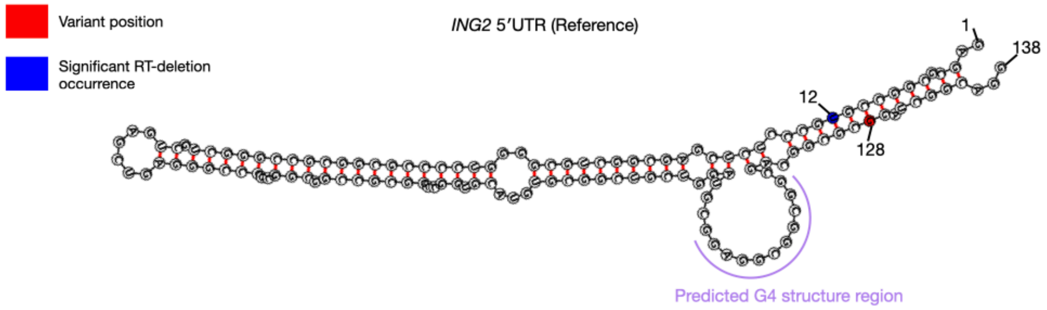

Supplementary Fig. 23 | Predicted RNA structure of the *ING2* 5′ UTR

Predicted RNA structure of the *ING2* 5′ UTR reference. Loop constraints were imposed on guanine-rich regions predicted to form G4 structures in RNAfold (**Supplementary Fig. 17b**). 12U is sequentially distant from the region containing the variant, but structurally close, suggesting that VQ modifies the base structurally close to the binding site.

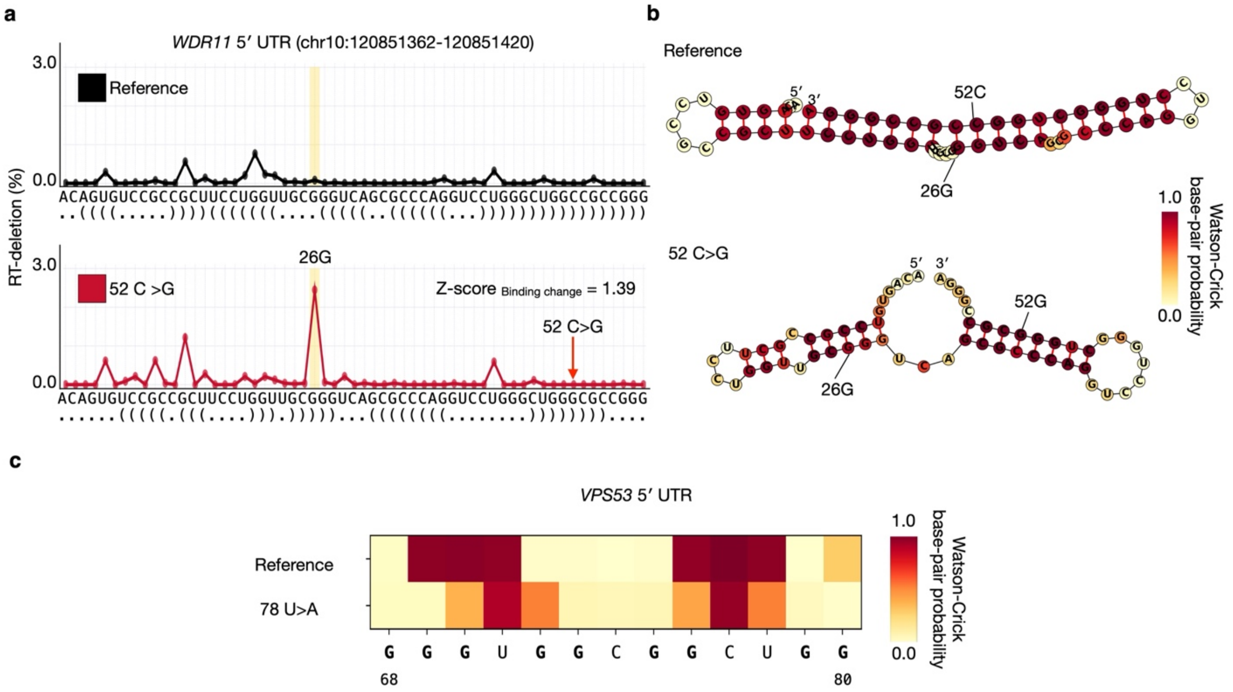

Supplementary Fig. 24 | SNV-induced RNA structural alteration and its effects on berberine binding

(a) RT-deletion profiles for the reference (black) and mutant (red) in *WDR11* 5′ UTR. The predicted RNA structure is shown under the sequence. The 52 C>G variant results in a significant increase in RT-deletion at 26 G, highlighted in yellow in all graphs. **(b)** *WDR11* 5′ UTR structure annotated with the Watson-Crick base-pair probability. **(c)** Watson-Crick base-pair probability of the G4 structure region in *VPS53* 5′ UTR. The G-tract-forming guanines are highlighted in bold. BIVID-MaP was performed in two independent experiments (n = 2). Source data are provided as a Source Data file.

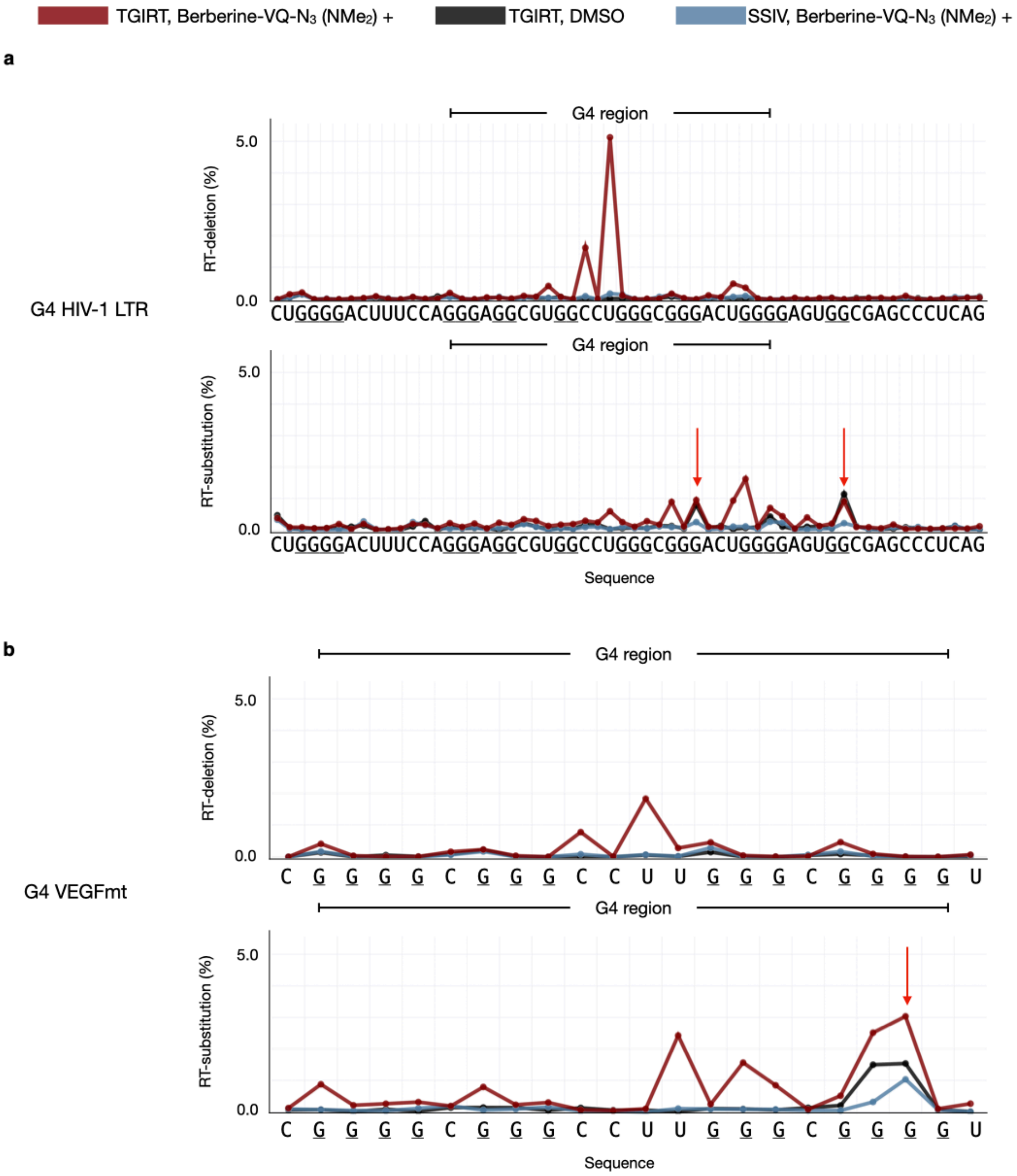

Supplementary Fig. 25 | RT substitution produces false positives rather than RT deletion

(a–b) RT-mutation profile for detecting berberine-G4 HIV-1 LTR interaction **(a)** or berberine-G4 VEGFmt interaction **(b).** RT was performed with TGIRT in the presence (red) or absence (black) of the modifier, and with SSIV in the presence of the modifier (blue). While deletions exhibit binding-dependent increases, substitutions often exhibit repetitive sequence-derived mutations, which are background mutations arising from TGIRT. The nucleotide positions showing the false positive signal are highlighted with red arrows. Repetitive guanines are highlighted by underlining. BIVID-MaP was performed in two independent experiments (n = 2). Source data are provided as a Source Data file.

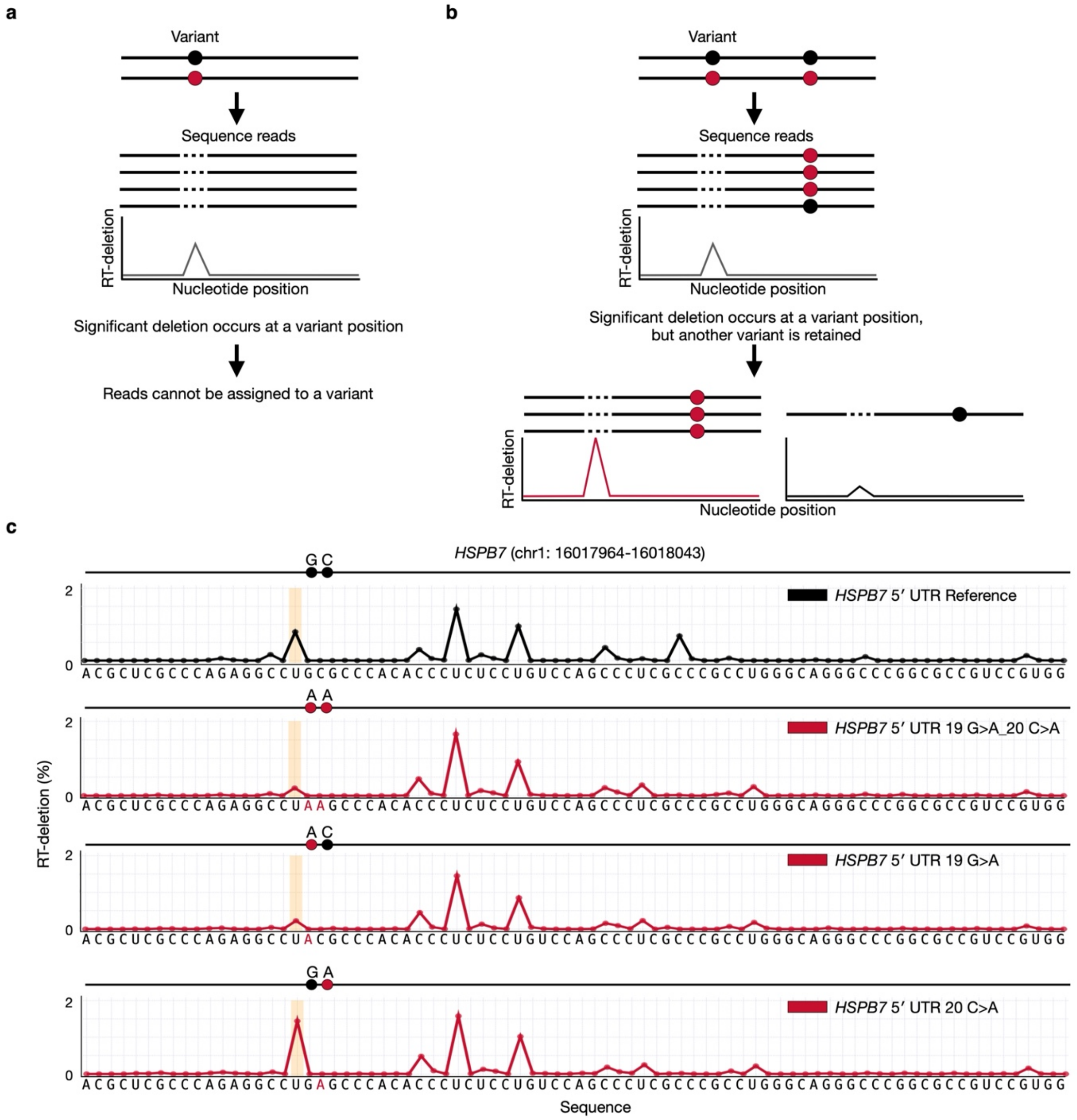

Supplementary Fig. 26 | RT deletion profiling of sequences with two variants

(a) Read identification of sequences with one nucleotide variant. If significant deletion occurs in variant position, the reads cannot be assigned to a specific variant. **(b)** Read identification of sequences with two nucleotide variants. Even if significant deletion occurs at a variant position, another variant can be used for read assignment. **(c)** An example of RT deletion profiling for sequences harboring two SNVs in the detection of the berberine-*HSPB7* 5′ UTR interaction. Reads corresponding to the reference sequence, the sequence with both C>A and G>A mutations, and the sequence with only one of the two mutations were recognized, with RT deletions detected for each variant. BIVID-MaP was performed in two independent experiments (n = 2). Source data are provided as a Source Data file.

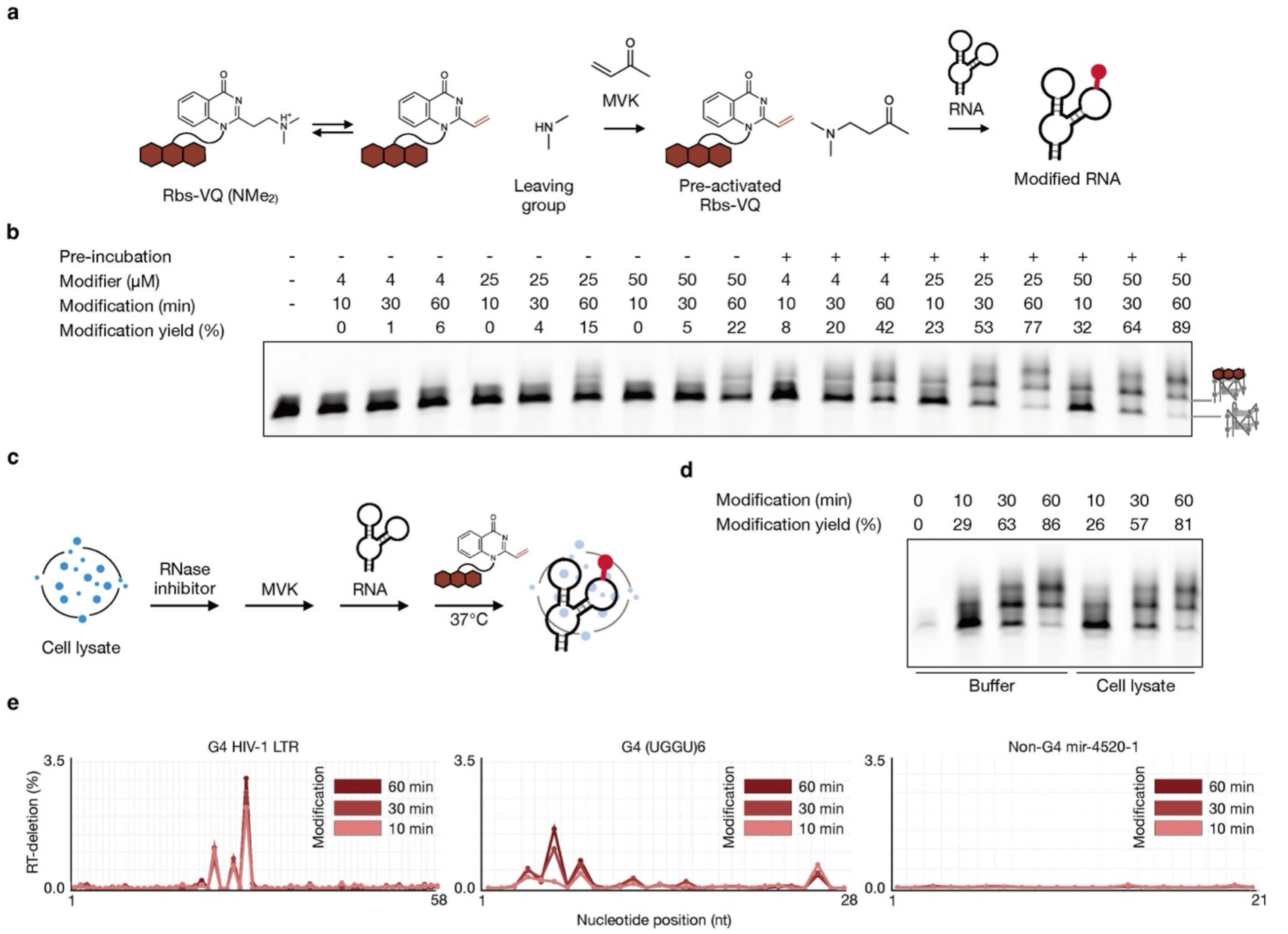

Supplementary Fig. 27 | Shortening modification time by pre-incubation of VQ precursor enables cell lysate application

(a) Workflow of the VQ pre-activation strategy. VQ is pre-activated by incubating the VQ precursor with methyl vinyl ketone (MVK). This strategy shortens the subsequent RNA modification time. **(b)** Gel shift assay showing modification of the G4 (UGGU)6 RNA by Berberine-VQ-N_3 (NMe_2) with or without pre-incubation in buffer (20 mM phosphate pH 7.0, 20 mM NaCl, 80 mM KCl). From bottom to top, the gel shift bands show unmodified RNA, a 1:1 covalent complex (one modifier per RNA) and a 1:2 covalent complex (two modifiers per RNA) (See Supplementary Note). Unmodified RNA was used as a size marker (See Source Data). **(c)** Workflow of the RNA modification experiment in HeLa cell lysate using the pre-activation strategy. **(d)** Gel shift assay showing modification of the G4 (UGGU)6 RNA by Berberine-VQ-N_3 (NMe_2) with pre-incubation in either cell lysate or buffer. **(e)** RT-deletion profile detecting RNA–berberine interactions in cell lysate using 2 μM RNA and 50 μM Berberine-VQ-N_3 (NMe_2) with modification times of 10, 30, and 60 minutes. G4 HIV-1 LTR and G4 (UGGU)6 are used as positive control RNA structures, while the non-G4 mir-4520-1 is used as a negative control. BIVID-MaP was performed in two independent experiments (n = 2). Representative gel images from three independent experiments are shown for (b) and (d). Source data are provided as a Source Data file.

Supplementary Notes

MALDI-TOF analysis indicates that the multiple gel-shift bands originate from 1:1 and 1:2 Rbs-VQ:RNA labeling stoichiometries

As multiple band shifts were observed in the gel shift assay (**Fig. 1d, Fig. 2e, Supplementary Fig. 7**), we verified their stoichiometric origin. MALDI-TOF analysis of model G4 (UGGU)6 revealed that both single and double alkylation events per RNA molecule occurred (**Supplementary Fig. 2**). This result suggests that the formation of multiple adducts on a single RNA molecule increases the probability of at least one RT deletion per molecule, thereby potentially amplifying the RT-deletion effectively.

Enrichment of modified RNA to increase RT deletion aimed at large-scale analysis, related to Supplementary Fig. 8

We synthesized a Berberine-VQ-N_3 (NMe_2) for click chemistry-based enrichment of modified RNAs (**Supplementary Fig. 8a, b**). As a proof of concept, we applied this approach to the RNA library containing six G4-containing sequences and six non-G4 sequences and compared RT deletion rates with and without enrichment. Enrichment significantly increased RT-deletion rates for the target G4 sequences, thus reducing the required sequencing depth (**Supplementary Fig. 8c, d**). It also improved specificity for the targeted RNA structures, as we observed a strong positive correlation between the chemical modification yield and the RT deletion rate (**Supplementary Fig. 7, Supplementary Fig. 8e**). These results indicate that the enrichment step enables BIVID-MaP to quantitatively analyze RNA-small molecule interactions. Additionally, we confirmed that almost all RT deletions were only one nucleotide long, with the exception of the highly repetitive sequence (UGGU) 6**.** This means sequencing reads remained nearly full-length except for the deleted nucleotide (**Supplementary Fig. 6b**). Based on this observation, we hypothesized that reads differing by just one nucleotide (e.g., an SNV) could be distinguished, allowing deletions to be distinguished for each variant individually.

The mechanistic differences between mutational profiling approaches can impact RNA-small molecule interaction detection

The limited detection capability of other mutational profiling approaches can be partially explained by their modification and detection preferences. DMS-MaPseq primarily detects mutation signals arising from DMS modifications at A and C bases. SHAPE-MaP, in contrast, modifies the ribose 2′-OH[2, 3]. However, direct ligand binding does not always occur at these specific sites or produce detectable changes in their reactivity. This may explain why these approaches do not consistently detect RNA–small molecule interactions, and why interactions that do not appreciably change DMS or SHAPE reactivity profiles are missed. Second, baseline background and reverse transcription performance can further limit detectability for highly structured RNAs. In our SHAPE-MaP measurements, G4 structure forming sequences exhibited elevated baseline mutation rates even under no-modifier/no-ligand control conditions. This higher background reduces the signal-to-noise ratio and can mask modifier- or ligand-

dependent changes, making interaction signals more difficult to detect in SHAPE-MaP compared with other methods in our validation system. Finally, nucleotide selectivity varies between methods, which can restrict the generation of signals for specific target sequences. BIVID-MaP uses U-dependent labelling, whereas DMS-MaPseq detects mutation signals at A and C bases. Consequently, U-free RNA (G4 pre-mir-6850 in **Supplementary Fig. 10**) is not detected by BIVID-MaP, and A/C-free RNA (G4 (UGGU)6 in **Supplementary Fig. 10**) is not detected by DMS-MaPseq. This reduces the overlap between the two methods. Together, these differences in modification preferences, reverse transcription performance, and nucleotide selectivity help explain the limited overlap among methods.

Detecting the berberine-5′UTR interaction with sequences harboring two SNVs, related to Supplementary Fig. 26

To investigate the effects of the two types of SNVs on the RNA-small molecule interactions, the 5′UTR somatic mutation library contains sequences with two-nucleotide mutants. In the *HSPB7* 5′UTR, 19 G>A and 20 C>A were mutations from the same patient (COSS2196308), so the library included a sequence with both mutations *(HSPB7* 5′UTR 19 G>A_20 C>A). The binding of berberine to the four sequences was simultaneously detected in BIVID-MaP. The binding calculated by BIVID signal was similar between the sequences. However, distinct deletion patterns at 18U were observed for each mutation. Interestingly, although the single-nucleotide mutant 20 C>A exhibited an increase in BIVID signal at 18U compared to the reference, both the two-nucleotide (19 G>A_20 C>A) and the single-nucleotide 19 G>A mutants demonstrated a decrease in BIVID signal at 18U (**Supplementary Fig. 26c**). Thus, 20 C>A causes the increase in BIVID signal at 18U, which is offset by the coexistence of 19 G>A, thus reducing the BIVID signal. This finding suggests that adjacent mutations may play an important role in modulating RNA structural formation and its interactions with small molecules in the 5′UTR.

Detailed information on the preparation of the 5′UTR somatic mutation library

Dial-out PCR of the 5′UTR somatic mutation library, related to Fig. 3

A Dial Primer was appended to the 3′ end of the DNA template. Distinct dial primer sequences were attached to the reference sequences and the single-nucleotide mutant sequences, the two-nucleotide mutant sequences, respectively (see **Supplementary Data 1**). The DNA template was then subjected to a dial-out PCR in a 25 μL reaction containing 1× Platinum SuperFi PCR Master Mix (Thermo Fisher Scientific), 1× SuperFi GC Enhancer (Thermo Fisher Scientific), 500 nM forward primer (complementary to the dial primer), 500 nM reverse primer, and the single-stranded DNA template (see Supplementary Data for primer details). The reaction mixture was initially heated to 98 °C for 30 s, followed by 16 or 20 cycles of 98 °C for 10 s, the optimized annealing temperature for 10 s, and 72 °C for 20 s (see **Supplementary Data 1**). After cycling, the reaction was held at 72 °C for 5 min and then cooled to 4 °C. After amplification, the resulting double-stranded DNA product was purified using a Monarch PCR & DNA Cleanup Kit (New England Biolabs). The dsDNA amplified by dial-out PCR was subsequently further amplified and appended with a T7 promoter sequence according to the protocol described

under the "DNA Template Amplification" in the Methods section. For *in vitro* transcription (IVT), reference sequences and single- and two-nucleotide mutant sequences, and the control sequences were transcribed separately. The resulting RNAs from these four categories were then mixed in equimolar amounts to generate the RNA library used in the subsequent modification reactions.

The design of two-nucleotide mutant sequences in the 5′UTR somatic mutation library

Variants selected from the COSMIC database that originate from the same patient (i.e., variants sharing the same ID number starting with 'COSS') and sequences incorporating both mutations were included in the library. For sequences with two nucleotide variants relative to these reference sequences, each variant was identified separately by classifying reads.

Detailed information on the deletion profiling analysis of the 5′UTR somatic mutation library

Threshold setting

To analyze only significant deletions, the read depth threshold was set to 1000. In addition, sequences with a relatively high number of deletion reads at variant positions were excluded. Specifically, the number of deletion reads detected at variant positions was defined as D-depth$_{\text{variant}}$, and the number of deletion reads detected at positions other than variant positions was defined as D-depth$_{\text{all}}$. Sequences satisfying the following Criterion (1) were removed.

$$(1)\ \frac{D - depth_{\text{variant}}}{D - depth_{\text{variant}} + D - depth_{\text{all}}} < 0.30$$

Filtering criteria for large-scale deletion profiling analysis

Since the sequence difference between the reference sequence and the variant sequences is only one base, read identification becomes less accurate under mixed conditions.

Therefore, as a control, we prepared conditions which the reference, single-, and two-nucleotide variant sequence groups were modified separately (divided condition). These were used to remove false-positive deletion peaks.

We calculated Z-scores for the deletion rate of each base in each sequence, and denoted the Z-score under mixed conditions for the base at position n as Zn (mixed) and the Z-score under divided conditions as Zn (divided). Sequences containing a nucleotide that met all of the following Criteria (2)–(4) were removed from the analysis.

$$(2)\ \left|Z_{n(\text{mixed})} - Z_{n(\text{divided})}\right| > 2.0$$

$$(3)\ \left(Z_{n(\text{mixed})} > 1.0 \land Z_{n(\text{divided})} < 1.0\right) \lor \left(Z_{n(\text{mixed})} < 1.0 \land Z_{n(\text{divided})} > 1.0\right)$$

$$(4)\ \left(DeletionRate_{n(\text{mixed})} > 2.0\right) \lor \left(DeletionRate_{n(\text{divided})} > 2.0\right)$$

In addition, although primer sequences are designed to independently form robust stem-loop structures, they can sometimes hybridize with the target sequence. To eliminate such structural interference, we calculated the Levenshtein distance between the predicted secondary structure of the primer regions within the full construct and their intended independent structures. We filtered the library to include only sequences where the distance was ≤4 for both forward and reverse primers. This criterion was applied to both reference and mutant sequences.

Synthesis of VQ-conjugated compounds

General

General chemicals were purchased from Wako Pure Chemical, Aldrich or the Tokyo Chemical Institute unless otherwise stated. ^{1}H-NMR spectra (400 MHz) were recorded on a Bruker 400 spectrometer. ^{1}H-NMR spectra (600 MHz) and ^{13}C-NMR spectra (150 MHz) were recorded on a Bruker AVANCE III 600 spectrometer or JEOL ECZ-600R. Gel visualization and quantification were performed using the ChemiDoc Touch MP imaging system and the Image Lab Software (Bio-Rad Laboratories, Inc.).

Synthesis of Berberine-VQ-N_3 (NMe_2)

The synthesis of **Berberine-VQ-N_3 (NMe_2)** starts from the amidation of starting material 2-amino-5-iodobenzoic acid to afford compound **2** followed by a substitution reaction to generate compound **3**. Subsequently, compound **3** undergoes a ring-closing reaction to afford compound **5** and by-product **4**. By-product **4** can be converted to compound **5** through a dehydration reaction. Trimethylsilyl (TMS) acetylene was coupled with compound **5** to generate compound **6** undergoing Sonogashira coupling, and then a deprotection reaction was performed to afford compound **7**. The azide handle was tethered after CuAAC (Copper-catalyzed azide-alkyne cycloadditions) reaction to obtain compound **8**, VQ(SMe)-azide. The carboxylic acid moiety compound **9**, was conjugated with berberine part (compound **14**) through amide coupling to obtain Berberine-VQ(SMe)-N_3. VQ-NMe_2 precursor was converted and purified using a previously reported procedure [8].

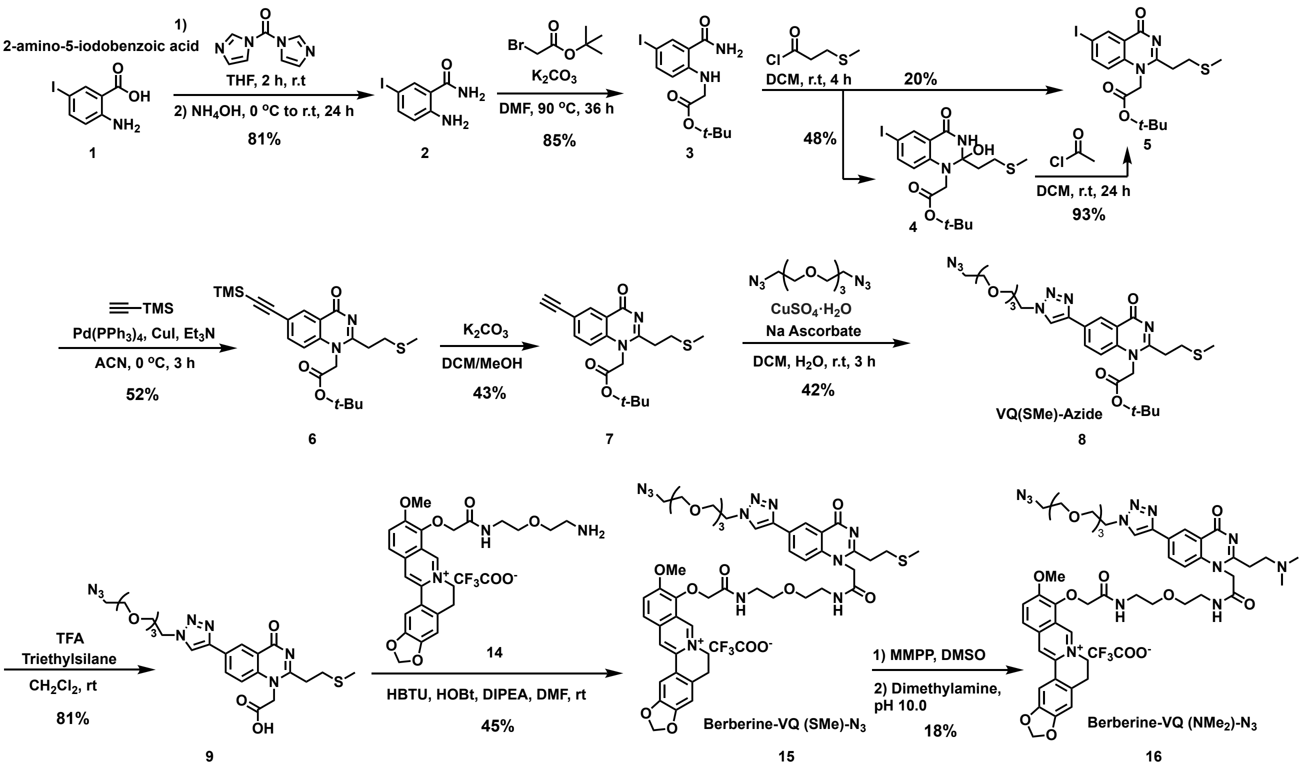

Scheme 1. Synthesis scheme of Berberine-VQ-N_3 (NMe_2).

Compound **14**, berberine-PEG-amine, was synthesized following Scheme 2. After obtaining the carboxylic moiety, compound **12**, amide coupling was performed to obtain compound **13** followed by Boc deprotection with TFA to produce berberine-PEG-amine.

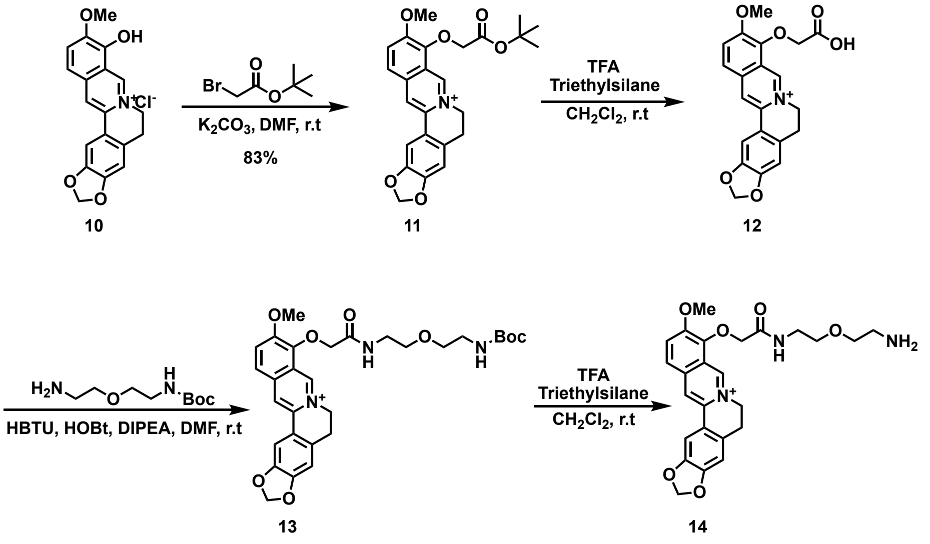

Scheme 2. Synthesis scheme for berberine-PEG-amine.

Synthesis of 2-amino-5-iodobenzamide (2)

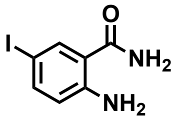

To a 1 L round-bottomed flask, 5-iodoanthranilic acid (20 g, 76 mmol) was added and dissolved in 200 mL of THF, followed by the addition of CDI (13.6 g, 83.6 mmol). After stirring for 2 h, NH_4OH (5.5 mol) was added to the mixture and stirred for 2 h. The THF was removed by evaporation. The crude was washed with water. The target material (**2**) was obtained in 81% yield (13.8 g) as a white solid.

^1H NMR (600 MHz, acetone-d_6) δ (ppm) 2.85 (s, 2H), 6.54 (s, 2H), 6.62 (d, J = 8.4, 1H), 7.41(dd, J = 1.8, 8.4 Hz, 1H), 7.85 (d, J = 1.8 Hz, 1H).

^{13}C NMR (150 MHz, acetone-d_6) δ (ppm) 74.5, 117.2, 120.0, 137.6, 141.2, 151.0, 171.0. HRMS (ESI-TOF) (*m/z*) calcd. for $C_7H_8IN_2O^+$ $[M+H]^+$ 262.9676, found 262.9667.

Synthesis of *tert*-butyl (2-carbamoyl-4-iodophenyl)glycinate (3)

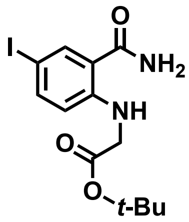

To a 10 mL round-bottomed flask, compound (**2**) (50 mg, 0.19 mmol) was added and dissolved in 500 μL of DMF, followed by the addition of K_2CO_3 (78 mg , 0.57 mmol) and *t*-butylbromoacetate (125 μL, 0.85 mmol). After stirring at 90 °C for 46 h, the reaction mixture was diluted with EtOAc and extracted with EtOAc /water three times. The organic layer was washed with brine, dried over Na_2SO_4 and evaporated in vacuo. The crude material was purified by silica gel chromatography ($CHCl_3$/MeOH = 9/1). The target material was obtained in 85% yield (61 mg) as a white solid.

^1H NMR (400 MHz, $CDCl_3$) δ (ppm) 1.48 (s, 9H), 3.84 (d, J = 5.2 Hz, 2H), 5.64 (br-s, 2H), 6.33 (d, J = 8.8 Hz, 1H), 7.52 (dd, J = 2.4, 8.8 Hz, 1H), 7.65 (d, J = 2.4 Hz, 1H), 8.17 (t, J = 5.2 Hz, 1H).

^{13}C NMR (150 MHz, DMSO-d_6) δ (ppm) 27.7, 44.9, 75.2, 81.0, 114.2, 117.0, 136.7, 140.4, 148.4, 169.46, 170.0. HRMS (ESI-TOF) (*m/z*) calcd. for $C_{13}H_{18}IN_2O^+$ $[M+H]^+$ 377.0357, found 377.0369.

Synthesis of *tert*-butyl 2-(2-hydroxy-6-iodo-2-(2-(methylthio)ethyl)-4-oxo-3,4-dihydroquinazolin-1(2H)-yl)acetate (4) and *tert*-butyl 2-(6-iodo-2-(2-(methylthio)ethyl)-4-oxoquinazolin-1(4H)-yl)acetate (5)

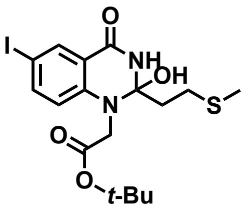

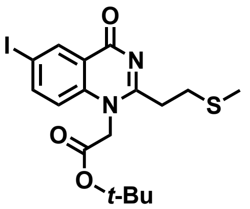

To a 200 mL round-bottomed flask, compound (**3**) (1.4 g, 3.7 mmol) was added and dissolved in 35 mL of DCM, followed by the addition of 3-methylthiopropionyl chloride (1.0 mL, 11 mmol). After stirring for 4 h, the reaction mixture was diluted with DCM and extracted with DCM/$NaHCO_3$aq three times. The organic layer was washed with brine, dried over Na_2SO_4, and evaporated in vacuo. The crude material was purified by silica gel chromatography (EtOAc/Hex = 1/1).

The target material (**5**) was obtained with a 20% yield (340 mg) as a white solid. The by-product (**4**) was obtained with a 48% yield (810 mg) as a white solid:

Compound (**4**) ^{1}H NMR (400 MHz, $CDCl_3$) δ (ppm) 1.48 (s, 9H), 2.13-2.42 (m, 1H), 2.49-2.56 (m, 1H), 2.63-2.79 (m, 3H), 4.21 (d, *J* = 17.1 Hz, 1H), 4.50 (d, *J* = 17.1 Hz, 1H), 6.25 (s, 1H), 7.03 (d, *J* = 8.4 Hz, 1H), 7.83 (dd, *J* = 2.0, 8.4 Hz, 1H), 8.27 (d, *J* = 2.0 Hz, 1H), 8.34 (s, 1H).

^{13}C NMR (150 MHz, $CDCl_3$) δ (ppm) 15.7, 28.1, 29.8, 33.5, 52.9, 83.3, 93.7, 129.7, 134.7, 134.6, 139.5, 140.4, 141.5, 166.9, 169.5, 172.6.

HRMS (ESI-TOF) (*m/z*) calcd. for $C_{17}H_{23}IN_2NaO_4S^+$ 501.0315; found 501.0325.

Compound (**5**) ^{1}H NMR (400 MHz, $CDCl_3$) δ (ppm) 1.48 (s, 9H), 2.17(s, 3H), 2.98-3.02(m, 2H), 3.06-3.10(m, 2H), 4.75 (s, 2H), 6.92 (d, *J* = 8.8 Hz, 1H), 7.96 (d, *J* = 2.4, 8.8 Hz, 1H), 8.69 (d, *J* = 2.4 Hz, 1H).

^{13}C NMR (150 MHz, $CDCl_3$) δ (ppm) 16.1, 28.0, 30.9, 35.3, 48.8, 84.8, 90.0, 115.8, 121.4, 137.8, 140.4, 142.5, 162.4, 165.4, 167.0.

HRMS (ESI-TOF) (*m/z*) calcd. for $C_{17}H_{22}IN_2O_3S^+$ [M+H]$^+$ 461.0390; found 461.0385.

Synthesis of *tert*-butyl 2-(6-iodo-2-(2-(methylthio)ethyl)-4-oxoquinazolin-1(4H)-yl)acetate (5)

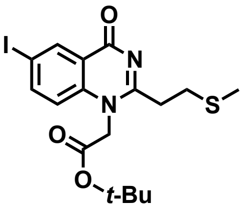

To a 200 mL round-bottomed flask, compound (**4**) (710 mg, 1.5 mmol) was added and dissolved in 21 mL DCM, followed by the addition of acetyl chloride (516 μL, 7.5 mmol). After stirring for 24 h, the reaction mixture was diluted with DCM and extracted with DCM/water three times. The organic layer was washed with brine, dried over Na_2SO_4, and evaporated in vacuo. The crude material was purified by silica gel chromatography ($CHCl_3$/MeOH = 10/1). The target material (**5**) was obtained with a 93% yield (642 mg) as a white solid.

Synthesis of *tert*-butyl 2-(2-(2-(methylthio)ethyl)-4-oxo-6-((trimethylsilyl)ethynyl)quinazolin-1(4H)-yl)acetate (6)

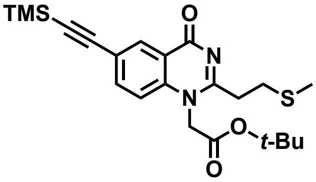

To a solution of (**5**) (149 mg, 324 μmol) in MeCN (1.5 mL), (trimethylsilyl)acetylene (225 μL, 1.63 mmol) and $Pd(PPh_3)_4$ (75.7 mg, 65.5 μmol) were added under ice bath conditions. After degassing 3 times, Et_3N (226 μL, 1.63 mmol) and CuI (13.1 mg, 68.8 μmol) were added. The reaction solution was warmed to room temperature and stirred for 3 h, followed by extraction with $CHCl_3$ (15 mL) and washed with water (15 mL) and brine (15 mL). The organic layer was then dried over Na_2SO_4 and concentrated. The crude compound was purified by silica gel column chromatography (DCM: Ethyl Acetate = DCM only →50:1→30:1→20:1→10:1) to afford a light yellow solid. (170 μmol, 52 %)

^{1}H NMR (400 MHz, $CDCl_3$) δ (ppm) 8.47 (1H, d, *J* = 1.6 Hz), 7.73 (1H, dd, *J* = 8.8, 2.0 Hz), 7.10 (1H, d, *J* = 8.8 Hz), 4.77 (2H, s), 3.11-3.07 (2H, m), 3.03-2.99 (2H, m), 2.18 (3H, s), 1.47 (9H, s), 0.25 (9H, s).

^{13}C NMR (150 MHz, $CDCl_3$) δ (ppm) -0.18, 16.1, 27.9, 30.9, 35.3, 48.9, 84.7, 96.6, 102.9, 113.9, 119.7, 121.3, 132.8, 136.7, 140.4, 162.3, 165.5, 167.7.

HRMS (ESI-TOF) (*m/z*) calcd. for $C_{22}H_{31}N_2O_3SSi^+$ $[M+H]^+$: 431.1819, found: 431.1836.

Synthesis of *tert*-butyl 2-(6-ethynyl-2-(2-(methylthio)ethyl)-4-oxoquinazolin-1(4H)-yl)acetate (7)

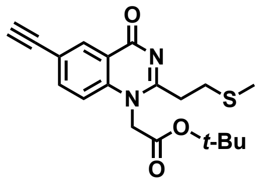

To a solution of (**6**) (70.0 mg, 163 µmol) in DCM (13 mL) and MeOH (27 mL), K_2CO_3 (113 mg, 816 µmol) was added under ice bath conditions. The reaction solution was stirred in an ice bath for 1 h, followed by cotton filtration. The mixture solution was extracted with DCM (150 mL) and washed with water (150 mL), NH_4Cl (100 mL), and brine (150 mL). The organic layer was then dried over Na_2SO_4 and concentrated. The crude compound was purified by silica gel column chromatography (DCM: Ethyl Acetate = DCM only →50:1→20:1→15:1→10:1) to afford a yellow solid. (44.1 µmol, 43 %)

^{1}H NMR (400 MHz, $CDCl_3$) δ (ppm) 8.48 (1H, d, *J* = 2.0 Hz), 7.77 (1H, dd, *J* = 8.8, 2.0 Hz), 7.13 (1H, d, *J* = 8.8 Hz), 4.78 (2H, s), 3.16 (1H, s), 3.11-3.07 (2H, m), 3.04-3.00 (2H, m), 2.18 (3H, s), 1.48 (9H, s).

^{13}C NMR (100 MHz, $CDCl_3$) δ (ppm) 167.8, 165.6, 162.6, 140.8, 137.2, 133.1, 120.4, 119.9, 114.2, 84.8, 81.8, 79.2, 49.0, 35.4, 31.1, 28.1, 16.2.

HRMS (ESI-TOF) (*m/z*) calcd. for $C_{19}H_{23}N_2O_3S^+$ $[M+H]^+$: 359.1424, found: 359.1423.

Synthesis of *tert*-butyl 2-(6-(1-(2-(2-(2-(2-azidoethoxy)ethoxy)ethoxy)ethyl)-1H-1,2,3-triazol-4-yl)-2-(2-(methylthio)ethyl)-4-oxoquinazolin-1(4H)-yl)acetate (8)

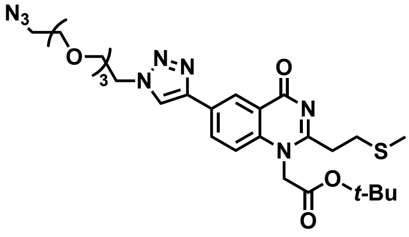

To a solution of **7** (39.0 mg, 109 µmol) in DCM (2 mL) and H_2O (1.6 mL), 1-azido-2-(2-(2-(2-azidoethoxy)ethoxy)ethoxy)ethane (254 mg, 1.05 mmol), sodium ascorbate (21.5 mg, 108 µmol), and copper(II) sulfate pentahydrate (5.2 mg, 22 µmol) were added at room temperature. The reaction solution was stirred for 3 h, followed by cotton filtration with DCM (60 mL). The filtrate was washed with water (60 mL) and brine (60 mL). The organic layer was then dried over Na_2SO_4 and concentrated. The crude compound was purified by silica gel column chromatography (Ethyl Acetate: MeOH = EA only →30:1) to afford a transparent oil. (45.9 µmol, 42 %)

^{1}H NMR (400 MHz, $CDCl_3$) δ (ppm) 8.53 (1H, d, J = 2.0 Hz), 8.48 (1H, dd, J = 8.8, 2.0 Hz), 7.29 (1H, s), 7.27 (1H, d, J = 8.8 Hz), 4.84 (2H, s), 4.62 (2H, t, J = 4.8 Hz), 3.93 (2H, t, J = 4.8 Hz), 3.67-3.63 (10H, m), 3.35 (2H, t, J = 4.8 Hz), 3.12-3.08 (2H, m), 3.06-3.02 (2H, m), 2.19 (3H, s), 1.49 (9H, s).
^{13}C NMR (100 MHz, $CDCl_3$) δ (ppm) 168.5, 165.8, 162.2, 146.0, 140.4, 131.4, 129.2, 125.1, 121.9, 120.1, 114.8, 84.7, 70.8, 70.7, 70.0, 69.4, 50.8, 50.6, 49.0, 35.3, 31.0, 28.0, 16.1.
HRMS (ESI-TOF) (*m/z*) calcd. for $C_{27}H_{39}N_8O_6S^+$ $[M+H]^+$: 603.2708, found: 603.2693.

Synthesis of 2-(6-(1-(2-(2-(2-(2-azidoethoxy)ethoxy)ethoxy)ethyl)-1H-1,2,3-triazol-4-yl)-2-(2-(methylthio)ethyl)-4-oxoquinazolin-1(4H)-yl)acetic acid (9)

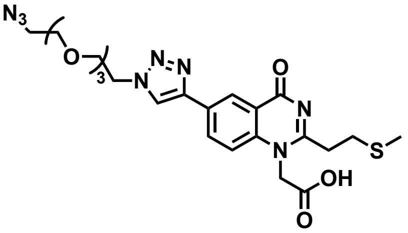

To a solution of (**8**) (47.1 mg, 78.2 μmol) in DCM (314 μL) triethyl silane (19 μL) and TFA (1.6 mL) were added at room temperature. The reaction was stirred for 1.5 h and concentrated using an evaporator. An excess amount of TFA was removed through co-evaporation with acetonitrile. The crude was roughly purified by silica gel column chromatography ($CHCl_3$: MeOH = 30:1→10:1) to afford a transparent oil. (63.5 μmol, 81 %)
HRMS (ESI-TOF) (*m/z*) calcd. for $C_{23}H_{31}N_8O_6S^+$ $[M+H]^+$: 547.2082, found: 547.2078.

Synthesis of 9-(2-(*tert*-butoxy)-2-oxoethoxy)-10-methoxy-5,6-dihydro-[1,3]dioxolo[4,5-*g*]isoquinolino[3,2-*a*]isoquinolin-7-ium (11)

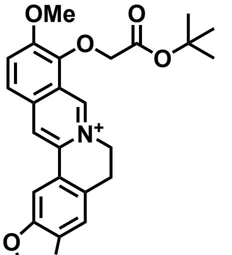

To a solution of (**10**) (15 mg, 42 μmol) in DMF (1.5 mL), K_2CO_3 (11.3 mg, 81.8 μmol) and *t*-butyl-2-bromoacetate (12.5 μL, 85.2 μmol) were added, with the reaction mixture changing to brown from yellow. After stirring at room temperature for 21 h, the reaction mixture was filtrated and a yellow solid precipitated on the cotton. The precipitate was dissolved in MeOH, with the solution evaporated to afford a yellow solid (5.5 mg, 13 μmol). The residue filtration liquor was recrystallized using EA: MeOH: hexane = 1.7 mL: 1 mL: 6 mL to afford a yellow fine powder. (7.0 mg, 16 μmol, total yield is 68%)
^{1}H NMR (400 MHz, DMSO-d_6) δ (ppm) 9.93 (1H, s), 8.95 (1H, s), 8.19 (1H, d, J = 6.0 Hz), 7.98 (1H, d, J = 6.0 Hz), 7.81(1H, s), 7.09 (1H, s), 6.18 (1H, s), 4.97 (2H, s), 4.94 (2H, t, J = 4.0 Hz), 4.03 (3H, s), 3.21 (2H, t, J = 4.0 Hz), 1.41 (9H, s).
^{13}C NMR (150 MHz, DMSO-d_6) δ (ppm) 167.9, 149.9, 148.9, 147.7, 145.7, 141.7, 137.6, 133.0, 130.7, 126.9, 123.1, 121.0, 120.4, 120.1, 108.5, 105.5, 102.1, 81.7, 69.6, 57.3, 55.4, 27.7, 26.4.
HRMS (ESI-TOF) (*m/z*) calcd. for $C_{25}H_{26}NO_6^+[M]^+$: 436.1755, found: 436.1766.

Synthesis of 9-(carboxymethoxy)-10-methoxy-5,6-dihydro-[1,3]dioxolo[4,5-*g*]isoquinolino[3,2-*a*]isoquinolin-7-ium (12)

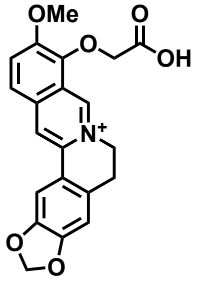

To a solution of (**11**) (7.0 mg, 16 µmol) in DCM (105 µL), triethyl silane (3.9 µL, 24 µmol) and TFA (420 µL) were added at room temperature, and the reaction mixture was stirred for 1 hr. After evaporation and co-evaporation with MeCN three times, the crude compound was purified by silica gel column chromatography (Ethyl Acetate: MeOH = 10:1→8:1→5:1→1:1→1:10) to afford a yellow solid. (3.5 mg, 9.2 µmol, 57%)

^{1}H NMR (400 MHz, DMSO-d_6) δ (ppm) 10.61 (1H, s), 8.82 (1H, s), 8.10 (1H, d, *J* = 9.2 Hz), 7.88 (1H, d, *J* = 8.8 Hz), 7.78 (1H, s), 7.09 (1H, s), 6.16 (2H, s), 4.87 (2H, t, *J* = 6 Hz), 4.43 (2H, s), 4.01 (3H, s), 3.20 (2H, t, *J* = 6 Hz).

^{13}C NMR (150 MHz, DMSO-d_6) δ (ppm) 170.4, 149.9, 149.4, 147.7, 146.3, 142.2, 137.4, 132.9, 130.6, 126.8, 123.1, 121.5, 120.5, 120.0, 108.5, 105.5, 102.1, 69.7, 57.2, 55.4, 26.4.

HRMS (ESI-TOF) (*m/z*) calcd. for $C_{21}H_{18}NO_6^+$[M]$^+$: 380.1129, found: 380.1139.

9-((2,2-dimethyl-4,12-dioxo-3,8-dioxa-5,11-diazatridecan-13-yl)oxy)-10-methoxy-5,6-dihydro-[1,3]dioxolo[4,5-*g*]isoquinolino[3,2-*a*]isoquinolin-7-ium (13)

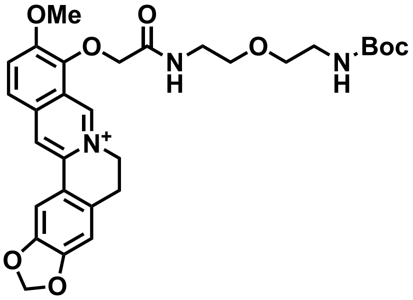

To a solution of (**12**) (11.4 mg, 30.0 µmol) in DMF (640 µL), DIPEA (15.7 µL, 90.0 µmol), HOBt (6.1 mg, 157.2 µmol), and HBTU (16.9 mg, 44.9 µmol) were added. After 30 min, *N*-(*tert*-Butoxycarbonyl)-2-(2-aminoethoxy)ethylamine (6.0 µL, 30 µmol) was added and reacted for 30 min. The reaction solution was concentrated using an oil pump to remove DMF and purified by silica gel column chromatography ($CHCl_3$: MeOH = 100:1→80:1→50:1) and precipitation (EA/MeOH/hexane =1 mL/0.6 mL/5 mL) to afford a yellow solid crude (16.6 mg).

HRMS (ESI-TOF) (*m/z*) calcd. for $C_{30}H_{36}N_3O_8^+$[M]$^+$: 556.2497, found: 556.2497.

9-(2-((2-(2-aminoethoxy)ethyl)amino)-2-oxoethoxy)-10-methoxy-5,6-dihydro-[1,3]dioxolo[4,5-*g*]isoquinolino[3,2-*a*]isoquinolin-7-ium (14)

To a solution of (**13**) (16.6 mg, 29.3 µmol) in DCM (200 µL), triethyl silane (7.0 µL, 44 µmol) and TFA (800 µL) were added at room temperature. The reaction was stirred for 50 min and concentrated by an

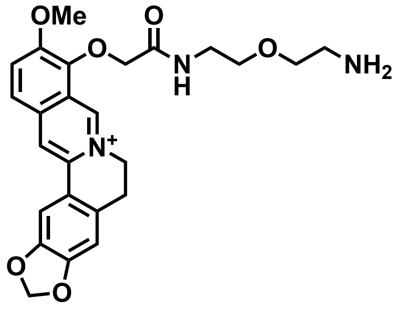

evaporator. Excess TFA was removed through co-evaporation with acetonitrile. The crude was purified by amino silica gel column chromatography ($CHCl_3$: MeOH = 5:1) to afford a yellow solid crude (9.4 mg).

HRMS (ESI-TOF) (*m/z*) calcd. for $C_{25}H_{28}N_3O_6^+$[M]$^+$: 466.1973, found: 466.1979.

Synthesis of 9-(2-((2-(2-(2-(6-(1-(2-(2-(2-(2-azidoethoxy)ethoxy)ethoxy)ethyl)-1*H*-1,2,3-triazol-4-yl)-2-(2-(methylthio)ethyl)-4-oxoquinazolin-1(4*H*)-yl)acetamido)ethoxy)ethyl)amino)-2-oxoethoxy)-10-methoxy-5,6-dihydro-[1,3]dioxolo[4,5-*g*]isoquinolino[3,2-*a*]isoquinolin-7-ium (15)

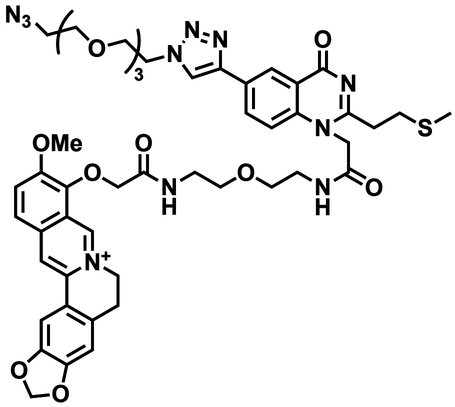

To a solution of (**9**) (9.7 mg, 18 μmol) in DMF (1.0 mL), DIPEA (9.3 μL, 53 μmol), and HOBt (3.7 mg, 27 μmol) were added. Compound (**14**) (8.3 mg, 18 μmol) dissolved in 1.0 mL DMF was added, followed by HBTU (39.8 mg, 52.7 μmol). At room temperature, the reaction mixture was stirred for 20 min then evaporated. The crude was dissolved in DMSO and membrane-filtrated (advantec 13 HPO45AN 0.45 μm). The filtration liquor was purified by HPLC to afford a yellow solid. (7.9 μmol, 45%)

^{1}H NMR (600 MHz, DMSO-*d*$_6$) δ (ppm) 9.87 (1H, s), 8.74 (1H, s), 8.63 (1H, s), 8.62 (1H, t, *J* = 6 Hz), 8.39 (1H, d, *J* = 2.4 Hz), 8.23 (1H, t, *J* = 6 Hz), 8.17 (1H, dd, *J* = 9, 1.8 Hz), 8.09 (1H, d, *J* = 9 Hz), 7.93 (1H, d, *J* = 9 Hz), 7.67 (1H, s), 7.53 (1H, d, *J* = 9 Hz), 7.02 (1H, s), 6.13 (2H, s), 4.99 (2H, s), 4.83 (2H, t, *J* = 6 Hz), 4.77 (2H, s), 4.54 (2H, t, *J* = 4.8 Hz), 3.99 (3H, s), 3.86 (2H, t, *J* = 4.8 Hz), 3.79 (2H, s), 3.54-3.52 (4H, m), 3.49-3.47(4H, m), 3.36 (2H, q, *J* = 11.4, 5.4 Hz), 3.32 (2H, q, *J* = 11.4, 5.4 Hz), 3.45 (4H, s), 3.28 (2H, t, *J* = 5.4 Hz), 3.15 (2H, t, *J* = 5.4 Hz), 3.05 (2H, s), 2.84 (2H, t, *J* = 7.8 Hz).

^{13}C NMR (150 MHz, DMSO-*d*$_6$) δ (ppm) 168.5, 166.5, 166.4, 163.3, 150.1, 150.0, 148.0, 145.9, 144.9, 142.0, 140.6, 137.6, 133.1, 131.0, 130.8, 128.8, 126.7, 124.1, 123.3, 122.7, 121.4, 120.5, 120.2, 119.9, 116.9, 108.7, 105.7, 102.4, 71.8, 70.0, 69.9, 69.8, 69.4, 68.9, 68.8, 68.7, 57.3, 55.7, 50.2, 50.0, 49.3, 38.7, 34.4, 30.1, 26.6, 15.1.

HRMS (ESI-TOF) (*m/z*) calcd. for $C_{48}H_{56}N_{11}O_{11}S^+$[M]$^+$: 994.3876, found: 994.3859.

Synthesis of 9-(2-((2-(2-(2-(6-(1-(2-(2-(2-(2-azidoethoxy)ethoxy)ethoxy)ethyl)-1H-1,2,3-triazol-4-yl)-2-(2-(dimethylamino)ethyl)-4-oxoquinazolin-1(4H)-yl)acetamido)ethoxy)ethyl)amino)-2-oxoethoxy)-10-methoxy-5,6-dihydro-[1,3]dioxolo[4,5-g]isoquinolino[3,2-a]isoquinolin-7-ium (16)

To a solution of compound (**15**) (4.0 μmol) in DMSO (203 μL), MMPP (1.2 mL, 24 μmol, 20 mM in water) and DMSO (600 μL) were added, the mixture was stirred at room temperature for 3 min. Then, carbonate buffer (pH = 10, 800 μL) and dimethylamine (400 μL, 200 μmol, 500 mM in H_2O) were added, and the mixture was incubated at 37 °C for 30 min. The solution was purified by HPLC to afford compound (**16**) (0.70 μmol, 18%).

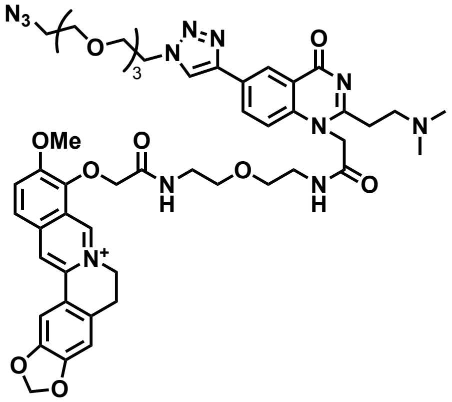

^{1}H NMR (600 MHz, DMSO-d_6) δ (ppm) 9.93 (1H, s), 8.86 (1H, s), 8.70 (2H, s), 8.47 (1H, d, *J* = 1.8 Hz), 8.28 (1H, s), 8.24 (1H, dd, *J* = 9, 1.8 Hz), 8.15 (1H, d, *J* = 9.6 Hz), 7.97 (1H, d, *J* = 9 Hz), 7.75 (1H, s), 7.55 (1H, d, *J* = 8.4 Hz), 7.07 (1H, s), 6.17 (2H, s), 5.02 (2H, s), 4.88 (2H, t, *J* = 6 Hz), 4.79 (2H, s), 4.55 (2H, t, *J* = 4.8 Hz), 4.02 (3H, s), 3.87 (2H, t, *J* = 4.8 Hz), 3.61 (2H, s), 3.55-3.54 (4H, m), 3.50 (6H, s), 3.47 (6H, s), 3.31-3.30 (6H, m), 3.19 (2H, t, *J* = 5.4 Hz), 2.88 (6H, s).

^{13}C NMR (150 MHz, DMSO-d_6) δ (ppm) 168.2, 167.0, 166.2, 161.1, 150.0, 149.9, 147.8, 145.8, 144.8, 141.9, 140.5, 137.6, 133.0, 130.8, 130.7, 128.6, 126.6, 123.9, 123.2, 122.5, 121.3, 120.4, 120.2, 119.8, 116.2, 108.5, 105.5, 102.2, 71.7, 69.9, 69.8, 69.7, 69.7, 69.3, 68.8, 68.7, 68.6, 57.2, 55.5, 53.5, 50.0, 49.8, 48.8, 42.7, 38.4, 34.4, 28.7, 26.4.

HRMS (ESI-TOF) (*m/z*) calcd. for $C_{49}H_{59}N_{12}O_{11}^+$[M]$^+$: 991.4421, found: 991.4401.

Synthesis of SMN-C2-VQ(NMe$_2$)

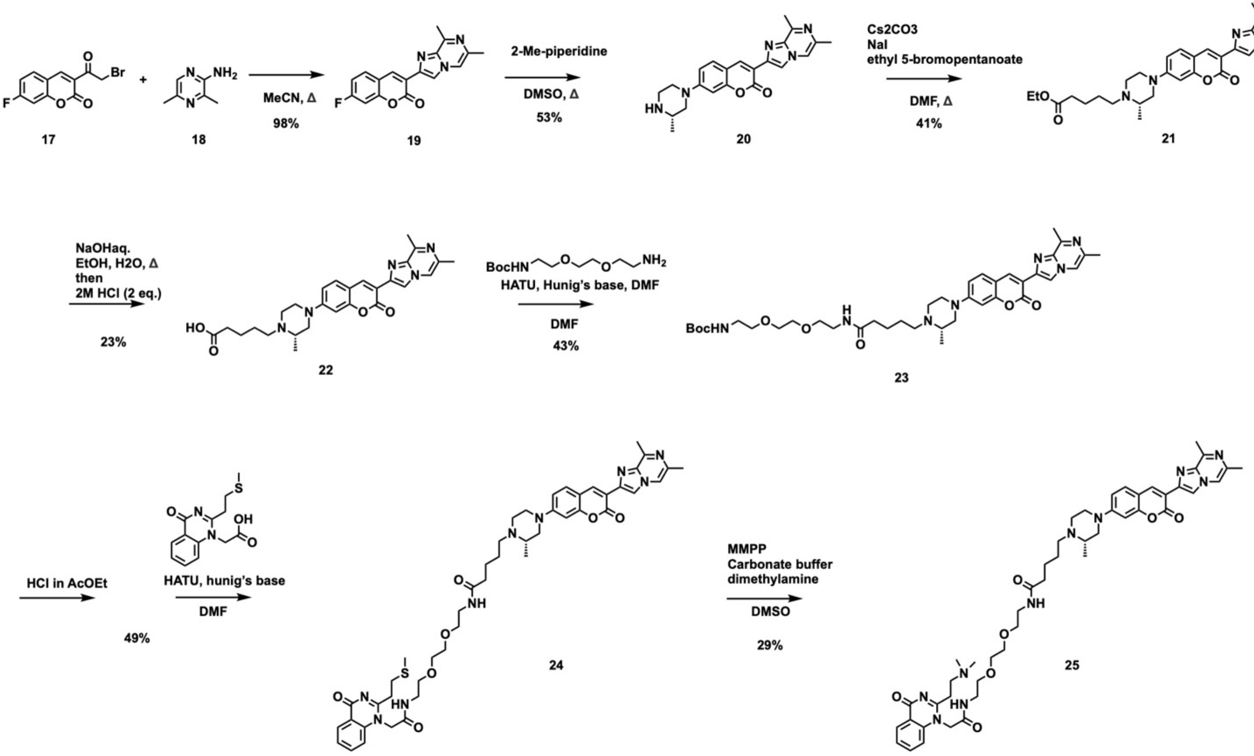

Scheme 3. Synthesis scheme of SMN-C2-VQ(NMe$_2$).

Synthesis of 3-(6,8-dimethylimidazo[1,2-a]pyrazin-2-yl)-7-fluoro-2H-chromen-2-one (19)

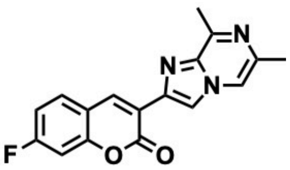

To a solution of (**17**) (600 mg, 2.1mmol) in MeCN (6 ml), (**18**) (233 mg, 1.89mmol) was added. After stirring at 110 °C for 5 h, the reaction mixture was cooled to room temperature, diluted with diisopropyl ether, filtered, and washed with diisopropyl ether several times. The filtered cake was dried in vacuo, leading to pale orange solid (638 mg, 98%).

^{1}H-NMR (600 MHz, DMSO-D6) δ 8.88 (s, 1H), 8.83 (s, 1H), 8.58 (s, 1H), 8.05 (dd, J = 9.0, 6.2 Hz, 1H), 7.45-7.43 (m, 1H), 7.30 (td, J = 8.8, 2.5 Hz, 1H), 2.91 (s, 3H), 2.45 (s, 3H)

^{13}C-NMR (151 MHz, DMSO-D6) δ 164.6 (d, $^1J_{C\text{-}F}$ = 252.9 Hz), 159.1, 154.6 (d, $^3J_{C\text{-}F}$ = 14.5 Hz), 150.1, 141.9, 139.9, 138.0, 132.5, 132.0(d, $^3J_{C\text{-}F}$ = 10.1 Hz), 118.4, 118.2, 117.5, 116.6, 113.5 (d, $^2J_{C\text{-}F}$ = 21.7 Hz), 104.3 (d, $^2J_{C\text{-}F}$ = 26.0 Hz), 18.5, 18.1

HRMS (ESI-TOF) (*m/z*) calcd. for $C_{17}H_{13}FN_3O_2$ [M+H]$^+$: 310.0992, found: 310.0986.

Synthesis of (S)-3-(6,8-dimethylimidazo[1,2-a] pyrazin-2-yl)-7-(3-methylpiperazin-1-yl)-2H-chromen-2-one (20)

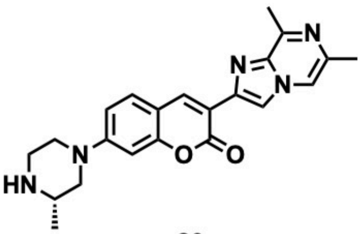

To a solution of (**19**) (1.2 g, 3.9 mmol) in DMSO (8ml), (S)-2-methylpiperazine (0.92ml, 7.8 mmol) and potassium carbonate (2.14 g, 15.5 mmol) were added. After stirring at 120 °C for 2 h, the reaction mixture was cooled to room temperature, diluted with water, and stirred for 30 min. The precipitate was filtered, and the filtered cake was washed with diisopropyl ether and dried in vacuo. The light brown solid was further purified by silica gel column chromatography to give this titled compound (**4**) (1.51 g, 53%) as a light brown solid.

^{1}H-NMR (600 MHz, DMSO-D6) δ 8.67 (s, 1H), 8.47 (s, 1H), 8.28 (s, 1H), 7.70 (d, J = 9.0 Hz, 1H), 6.99 (dd, J = 9.0, 2.8 Hz, 1H), 6.84 (d, J = 2.8 Hz, 1H), 3.81 (t, J = 11.4 Hz, 2H), 2.99-2.97 (m, 1H), 2.77-2.73 (m, 6H), 2.41 (dd, J = 12.1, 10.7 Hz, 1H), 2.35 (s, 3H), 1.05 (d, J = 6.2 Hz, 3H)

^{13}C-NMR (151 MHz, DMSO-D6) δ 159.6, 155.1, 153.4, 149.5, 139.1, 138.9, 138.0, 136.1, 129.7, 115.0, 113.7, 113.2, 111.5, 109.7, 98.9, 53.8, 49.9, 46.8, 44.8, 20.2, 20.1, 19.0

HRMS (ESI-TOF) (*m/z*) calcd. for $C_{22}H_{24}N_5O_2$ [M+H]$^+$: 390.1930, found: 390.1933.

Synthesis of ethyl (S)-5-(4-(3-(6,8-dimethylimidazo[1,2-a] pyrazin-2-yl)-2-oxo-2H-chromen-7-yl)-2-methylpiperazin-1-yl) pentanoate (21)

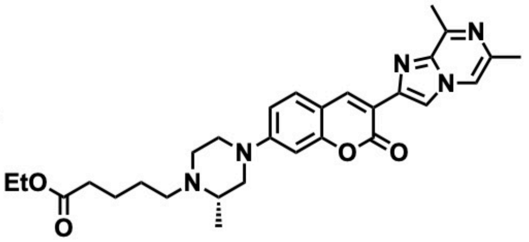

To a solution of (**20**) (60 mg, 0.15 mmol) in DMF (1 ml), cesium carbonate (100 mg, 0.31 mmol), sodium iodide (69.3 mg, 0.46 mmol), and ethyl 5-bromopentanoate (49.2 μL, 0.31 mmol) were added. After stirring at 80 °C for 2 h, the reaction mixture was cooled to room temperature and diluted with AcOEt and water. The organic layer was washed with brine, dried by sodium sulfate, and concentrated. The crude mixture was further purified by silica gel column chromatography eluting CHCl3 and MeOH to give this titled compound (**5**) (32.4 mg, 41%) as a yellow solid.

^{1}H-NMR (600 MHz, DMSO-D6) δ 8.71 (s, 1H), 8.51 (s, 1H), 8.31 (s, 1H), 7.74 (d, J = 9.0 Hz, 1H), 7.02 (dd, J = 9.0, 2.1 Hz, 1H), 6.88 (d, J = 2.8 Hz, 1H), 4.05 (q, J = 7.1 Hz, 2H), 3.73-3.70 (m, 2H), 3.07-3.03 (m, 1H), 2.88-2.86 (m, 1H), 2.75-2.75 (m, 4H), 2.69 (dt, J = 14.5, 6.5 Hz, 1H), 2.45-2.42 (m, 1H), 2.36 (s, 3H), 2.32 (t, J = 7.6 Hz, 2H), 2.27-2.17 (m, 2H), 1.61-1.41 (m, 4H), 1.18 (t, J = 7.2 Hz, 3H), 1.05 (d, J = 6.2 Hz, 3H)

13C-NMR (151 MHz, DMSO-D6) δ 172.8, 159.6, 155.1, 153.2, 149.6, 139.1, 139.0, 138.0, 136.1, 129.7, 115.1, 113.7, 113.2, 111.5, 109.7, 98.9, 59.6, 54.1, 53.3, 52.0, 49.6, 46.8, 33.2, 24.7, 22.4, 20.2, 20.1, 15.4, 14.0

HRMS (ESI-TOF) (*m/z*) calcd. for $C_{29}H_{36}N_5O_4$ [M+H]$^+$: 518.2767, found: 518.2768.

Synthesis of (S)-5-(4-(3-(6,8-dimethylimidazo[1,2-a] pyrazin-2-yl)-2-oxo-2H-chromen-7-yl)-2-methylpiperazin-1-yl) pentanoic acid (22)

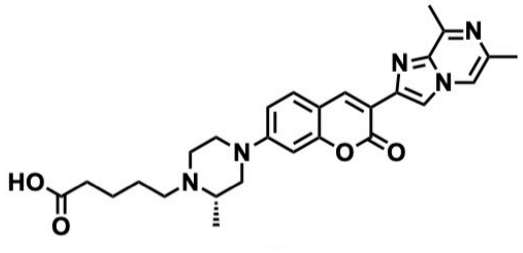

To a solution of (**21**) (32 mg, 0.061 mmol), water (0.33 ml) and 4M aqueous NaOH solution were added. After stirring at 60 °C for 10min, the suspended solution was dissolved. The reaction was completed after 1 h stirring and quenched with 2M aqueous HCl solution in an iced bath to pH 7. The mixture was concentrated and purified by C18 column chromatography eluting H2O and MeCN to give this titled compound (**6**) (7mg, 23%) as a yellow solid.

^{1}H-NMR (600 MHz, DMSO-D6) δ 8.63 (d, J = 13.1 Hz, 1H), 8.45 (s, 1H), 8.27 (s, 1H), 7.69 (d, J = 9.0 Hz, 1H), 6.98 (dd, J = 9.0, 2.1 Hz, 1H), 6.83 (d, J = 2.1 Hz, 1H), 3.69-3.67 (m, 2H), 3.03-2.99 (m, 1H), 2.87-2.83 (m, 1H), 2.76-2.62 (m, 5H), 2.43-2.37 (m, 1H), 2.33 (s, 3H), 2.26-2.14 (m, 2H), 2.07 (t, J = 6.5 Hz, 2H), 1.53-1.40 (m, 4H), 1.05 (d, J = 6.2 Hz, 3H)

^{13}C-NMR (151 MHz, DMSO-D6) δ 175.4, 159.5, 155.0, 153.1, 149.5, 139.1, 138.9, 138.0, 136.0, 129.7, 115.0, 113.6, 113.2, 111.4, 109.7, 98.8, 54.1, 53.3, 52.4, 49.7, 46.7, 35.6, 25.2, 23.3, 20.2, 20.1, 15.5

HRMS (ESI-TOF) (*m/z*) calcd. for $C_{27}H_{32}N_5O_4$ $[M+H]^+$: 490.2454, found: 490.2456.

Synthesis of tert-butyl (S)-(2-(2-(2-(5-(4-(3-(6,8-dimethylimidazo[1,2-a]pyrazin-2-yl)-2-oxo-2H-chromen-7-yl)-2-methylpiperazin-1-yl)pentanamido)ethoxy)ethoxy)ethyl)carbamate (23)

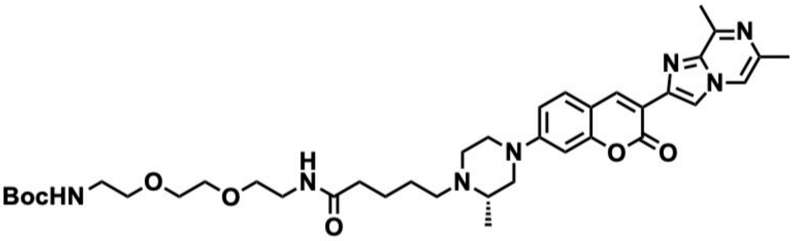

To a solution of (**22**) (7 mg, 0.014 mmol) in DMF (200 μL) were added tert-butyl (2-(2-(2-aminoethoxy)ethoxy)ethyl)carbamate (5.33 mg, 0.021mmol), HATU (8.15 mg, 0.029 mmol) and N-ethyl-N-isopropylpropan-2-amine (5.1 μL 0.029 mmol). After stirring at room temperature for 3 h, the reaction was quenched with water and diluted with AcOEt. The organic layer was washed with brine, dried by sodium sulfate, and concentrated. The crude mixture was purified by ODS column chromatography eluting H2O and MeCN, concentrated, and dried in vacuo to give this titled compound (**7**) (4.4 mg, 43%) as a yellow solid.

^{1}H-NMR (600 MHz, DMSO-D6) δ 8.73 (s, 1H), 8.52 (s, 1H), 8.32 (s, 1H), 7.85 (t, J = 5.5 Hz, 1H), 7.75 (d, J = 9.0 Hz, 1H), 7.03 (dd, J = 9.0, 2.8 Hz, 1H), 6.89 (d, J = 2.1 Hz, 1H), 6.78 (t, J = 5.5 Hz, 1H), 3.72 (d, J = 9.0 Hz, 2H), 3.49 (s, 4H), 3.38 (dt, J = 15.4, 6.0 Hz, 4H), 3.19 (q, J = 5.7 Hz, 2H), 3.05 (q, J = 6.2 Hz, 3H), 2.87 (d, J = 11.7 Hz, 1H), 2.78-2.74 (m, 4H), 2.70-2.65 (m, 1H), 2.61 (t, J = 1.7 Hz, 0H), 2.45-2.42 (m, 1H),

2.39-2.37 (m, 3H), 2.25 (t, J = 9.3 Hz, 1H), 2.21-2.17 (m, 1H), 2.10-2.05 (m, 2H), 1.56-1.37 (m, 13H), 1.06 (d, J = 6.2 Hz, 3H)

^{13}C-NMR (151 MHz, DMSO-D6) δ 172.0, 159.6, 155.5, 155.1, 153.2, 149.6, 139.1, 139.1, 138.1, 136.1, 129.8, 115.1, 113.7, 113.2, 111.5, 109.7, 98.9, 77.5, 69.4, 69.3, 69.1, 69.1, 54.1, 53.3, 52.2, 49.6, 46.8, 40.3, 38.3, 35.0, 28.1, 28.1, 28.1, 24.9, 23.2, 20.2, 20.1, 15.4

HRMS (ESI-TOF) (*m/z*) calcd. for $C_{38}H_{54}N_7O_7$ $[M+H]^+$: 720.4085, found: 720.4080.

Synthesis of (*S*)-5-(4-(3-(6,8-dimethylimidazo[1,2-*a*]pyrazin-2-yl)-2-oxo-2*H*-chromen-7-yl)-2-methylpiperazin-1-yl)-*N*-(2-(2-(2-(2-(2-(2-(methylthio)ethyl)-4-oxoquinazolin-1(4*H*)-yl)acetamido)ethoxy)ethoxy)ethyl)pentanamide (24)

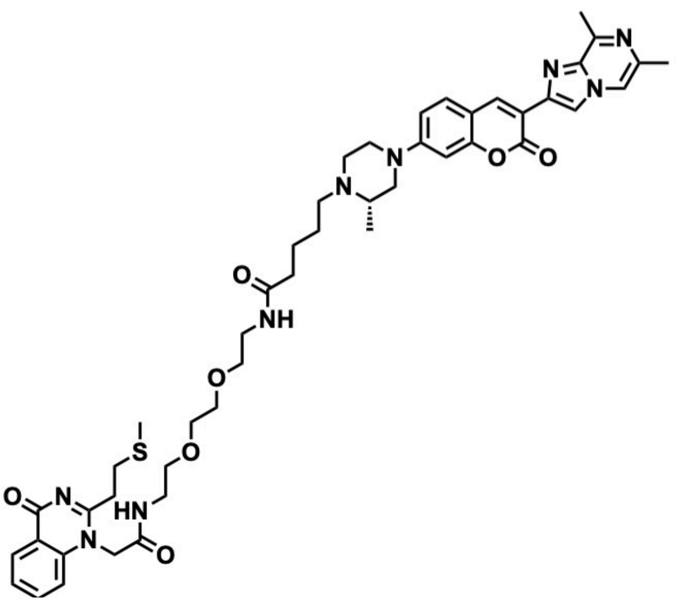

To a solution of (**23**) (26 mg, 0.038 mmol), 4M of HCl-AcOEt (300 µL) solution was added, and the reaction mixture was stirred for 10 minutes. A red precipitate appeared in the solution, and the reaction was monitored by TLC. The precipitate was filtered and washed with AcOEt, leading to a dark brown solid (20 mg). The crude product was used without further purification. To a solution of the crude mixture in DMF (600 µL), 2-(2-(2-(methylthio)ethyl)-4-oxoquinazolin-1(4*H*)-yl)acetic acid[9] (9.06 mg, 0.033mmol), HATU (24.8 mg, 0.065 mmol), and N-ethyl-N-isopropylpropan-2-amine (17.4 µL, 0.098 mmol) were added. After stirring at room temperature for 3 h, the reaction was quenched with water and diluted with AcOEt. The organic layer was washed with brine, dried by sodium sulfate, and concentrated. The crude mixture was purified by ODS column chromatography eluting H2O and MeCN, concentrated, and dried in vacuo, to give this titled compound (**8**) as a yellow solid. The obtained compound was further purified by HPLC (1.29 mg, 22.2%).

^{1}H NMR (500 MHz, DMSO-d_6) δ (ppm) 8.76 (1H, s), 8.63 (1H, t, *J* = 5.5 Hz), 8.58 (1H, s), 8.37 (1H, s), 8.12 (1H, d, *J* = 7.5 Hz), 7.93 (1H, t, *J* = 5.0 Hz), 7.83 (1H, t, *J* = 7.5 Hz), 7,81 (1H, d, *J* = 8.5 Hz), 7.54 (1H, t, *J* = 8.0 Hz), 7.53 (1H, d, *J* = 9.0 Hz), 7.10 (1H, d, *J* = 8.0 Hz), 7.03 (1H, s), 5.04 (2H, s), 4.15 (2H, t), 3.51 (14H, s), 3.45 (2H, t, *J* = 5.5 Hz), 3.40 (2H, t, *J* = 5.5 Hz), 3.29 (2H, q, *J* = 5.5 Hz), 3.20 (2H, q, *J* = 5.5 Hz),

3.12 (2H, t), 3.05 (2H, br), 2.88 (2H, t, *J* = 5.5 Hz). 2.79 (3H, s), 2.38 (3H, s), 2.16-2.12 (1H, m), 2.12 (3H, s), 2.06 (3H, s)

^{13}C NMR (125 MHz, DMSO-*d*$_6$) δ (ppm) 172.1, 166.1, 163.4, 159.6, 155.1, 149.7, 141.1, 140.0, 139.5, 138.1, 135.4, 134.6, 130.2, 127.5, 126.5, 119.5, 118.3, 116.0, 116.0, 114.4, 112.3, 111.1, 100.4, 69.7, 69.2, 68.9, 57.5, 56.2, 51.9, 50.7, 49.3, 44.4, 38.6, 34.5, 34.3, 30.1, 22.6, 22.4, 19.7, 15.0, 14.2.

HRMS (ESI-TOF) calcd. for $C_{46}H_{58}N_9O_7S^+$ [M+H]$^+$: 880.4175, found: 880.4163.

Synthesis of (*S*)-*N*-(2-(2-(2-(2-(2-(2-(dimethylamino)ethyl)-4-oxoquinazolin-1(4*H*)-yl)acetamido)ethoxy)ethoxy)ethyl)-5-(4-(3-(6,8-dimethylimidazo[1,2-*a*]pyrazin-2-yl)-2-oxo-2*H*-chromen-7-yl)-2-methylpiperazin-1-yl)pentanamide (25)

r

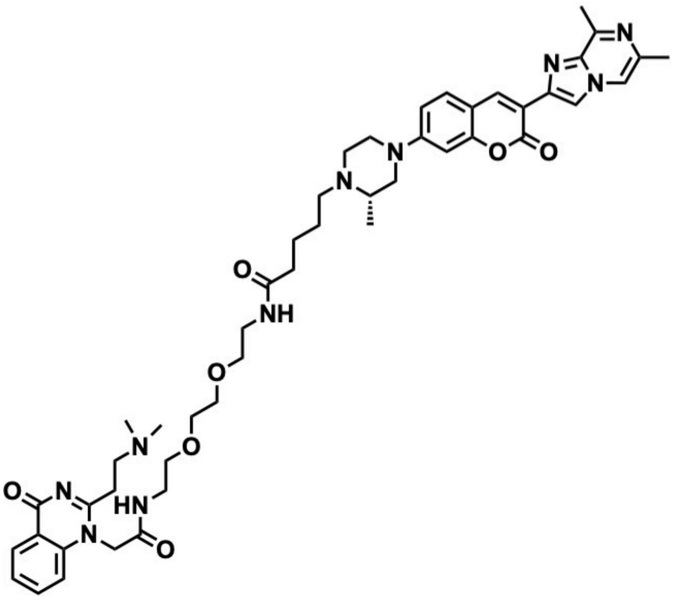

To a solution of (**24**) (4 μmol) in DMSO (400 μL), MMPP (1.2 mL, 24 μmol, 20 mM in water) and DMSO (600 μL) were added, and the mixture was stirred at room temperature for 3 min. Then, carbonate buffer (pH = 10, 800 μL) and dimethylamine (400 μL, 200 μmol, 500 mM in H2O) were added, and the mixture was incubated at 37 °C for 30 min. The solution was purified by HPLC to afford the desired product (1.16 μmol, 29%).

^{1}H NMR (600 MHz, DMSO-*d*$_6$) δ (ppm) 8.78 (1H, s), 8.68 (1H, t, *J* = 4.8 Hz), 8.55 (1H, d, *J* = 4.8 Hz), 8.34 (1H, s), 8.07 (1H, dd, *J* = 7.2, 1.2 Hz), 7.93 (1H, t, *J* = 4.2 Hz), 7.83-7.81 (2H, m), 7.53-7.7.51 (1H, m), 7.49-7.47 (1H, m), 7.11 (1H, s), 7.06 (1H, s), 6.87 (1H, s), 5.00 (2H, s), 4.17 (2H, t, *J* = 16.2 Hz), 3.67 (2H, s), 3.59 (2H, t, *J* = 10.8 Hz), 3.54 (2H,s), 3.51 (8H,s), 3.31-3.30 (8H, s), 3.21 (4H, t, *J* = 5.4 Hz), 3.05 (2H, d, *J* = 9.0 Hz), 2.87 (6H, s), 2.76 (3H, s), 2.55-2.53 (1H, m), 2.37 (3H, s), 2.15 (3H, s).

^{13}C NMR (150 MHz, DMSO-*d*$_6$) δ (ppm) 171.9, 167.0, 166.2, 161.2, 159.6, 155.0, 151.9, 149.8, 141.1, 139.2, 139.1, 138.2, 136.1, 134.3, 130.1, 127.5, 126.0, 119.4, 115.4, 115.2, 113.8, 112.2, 111.0, 100.4, 69.6, 69.6, 69.2, 68.9, 57.3, 53.4, 51.8, 50.5, 49.3, 48.7, 44.2, 42.7, 38.5, 34.4, 28.8, 22.4, 22.3, 20.2, 20.1, 14.1.

HRMS (ESI-TOF) calcd. for $C_{47}H_{61}N_{10}O_7$ [M+H]$^+$: 877.4720, found: 877.4701.

Synthesis of VQ-N_3

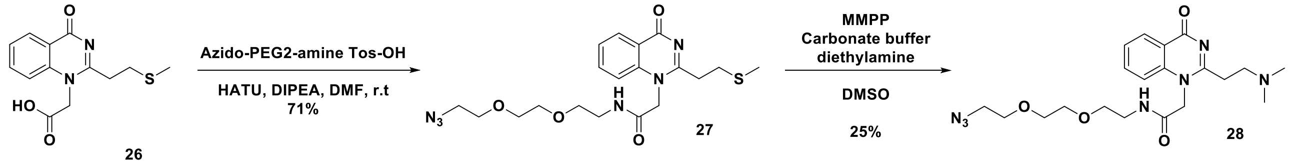

Scheme 4. Synthesis scheme of VQ-N_3.

Synthesis of *N*-(2-(2-(2-azidoethoxy)ethoxy)ethyl)-2-(2-(2-(methylthio)ethyl)-4-oxoquinazolin-1(4H)-yl)acetamide (27)

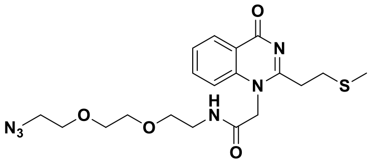

To a solution of **26** (20 mg, 0.07 mmol) in DMF (1.0 mL) were added DIPEA (37.5 µL, 0.21 mmol), azido-PEG2-amine Tos-OH (25 mg, 0.07 mmol), and HATU (81 mg, 0.21 mmol) then the reaction mixture was stirred at room temperature for 2.5 h. The reaction mixture was quenched with 0.1% TFA in water (0.2 mL).The reaction mixture was then concentrated and co-evaporated with acetonitrile three times. The crude was dissolved in DMSO (0.8 mL) and filtered with membrane (advantec, 13HP045AN, 0.45 µmol), washed with DMSO (0.6 mL), and purified by HPLC to afford compound **27** (51 µmol, 71%). HPLC conditions: A: 0.1% TFA in distilled water, B: 0.1% TFA in MeCN, B: 5%→40% (0-20 min) → 100% (-32 min). Flow rate = 4 mL/min; Temp. =35.0 °C; UV = 254 nm, C-18 column (Nacalai tesque : COSMOSIL 5C18-AR-II, 10×250 mm).

^{1}H NMR (600 MHz, DMSO-d_6) δ (ppm) 8.67 (1H, t, *J* = 5.4 Hz), 8.14 (1H, d, *J* = 7.8 Hz), 7.86 (1H, t, *J* = 7.8 Hz), 7.58 (1H, d, *J* = 7.8 Hz), 7.56 (1H, t, *J* = 7.8 Hz), 5.09 (2H, s), 3.60 (2H, t, *J* = 4.8 Hz), 3.58-3.52 (4H, m), 3.47 (2H, t, *J* = 5.4 Hz), 3.39 (2H, t, *J* = 5.4 Hz), 3.32-3.29 (2H, m), 3.16 (2H, t, *J* = 7.2 Hz), 2.89 (2H, t, *J* = 7.2 Hz), 2.13 (3H, s).

^{13}C NMR (151 MHz, DMSO-d_6) δ (ppm) 165.8, 165.0, 163.4, 140.8, 134.8, 127.4, 126.7, 119.5, 116.2, 69.7, 69.6, 69.3, 68.9, 50.0, 49.4, 39.0, 34.0, 30.0,14.8.

HRMS (ESI-TOF) (*m/z*) calcd. for $C_{19}H_{27}N_6O_4S^+$ [M+H]$^+$: 435.1814, found: 435.1818.

Synthesis of *N*-(2-(2-(2-azidoethoxy)ethoxy)ethyl)-2-(2-(2-(dimethylamino)ethyl)-4-oxoquinazolin-1(4H)-yl)acetamide (28)

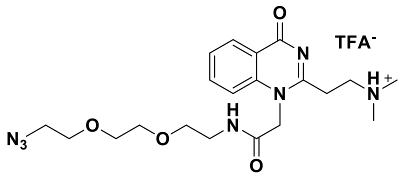

To a solution of **27** (4.6 µmol) in DMSO (23 µL) was added a solution of MMPP (46 µmol) in water (200 µL). The mixture was stirred at room temperature for 3 min. Carbonate buffer (pH 10, 50 mM, 370 µL) and dimethylamine (460 µmol) in water (50 µL) were then added and the mixture was incubated at 37 °C for 30 min. DMSO (2 mL) was added and the solution was filtered with membrane (advantec, 13HP045AN, 0.45 µmol), washed with DMSO (1 mL) and purified by HPLC to afford compound **28** (1.2 µmol, 25%). HPLC conditions: A: 0.1% TFA in distilled water, B:

0.1% TFA in MeCN; B: 5%→40% (0-25 min)→100%(-28 min). Flow rate = 4 mL/min; Temp. = 35.0 °C; UV = 254 nm, C-18 column (Nacalai tesque: COSMOSIL 5C18-AR-II, 10×250 mm).

^{1}H NMR (600 MHz, DMSO-d_6) δ (ppm) 9.18 (1H, brs), 8.66 (1H, s), 8.11 (1H, d, J = 7.8 Hz), 7.83 (1H, t, J = 7.8 Hz), 7.52 (1H, t, J = 7.8 Hz), 7.48 (1H, d, J = 7.8 Hz) 5.00 (2H, s), 3.60 (2H, t, J = 4.8 Hz), 3.58-3.53 (8H, m), 3.50-3.46 (2H, m), 3.41-3.38 (2H, m), 2.87 (6H, s), 2.55-2.53 (2H, m).

^{13}C NMR (151 MHz, DMSO-d_6) δ (ppm) 166.9, 166.1, 161.1, 141.1, 134.2, 127.5, 125.9, 119.4, 115.2, 69.7, 69.6, 69.3, 68.9, 53.4, 50.0, 48.6, 42.6, 34.4, 28.8.

HRMS (ESI-TOF) (m/z) calcd. for $C_{20}H_{30}N_7O_4^+$ $[M+H]^+$: 432.2359, found: 432.2355.

Synthesis of CMA-VQ-N_3 (NMe_2)

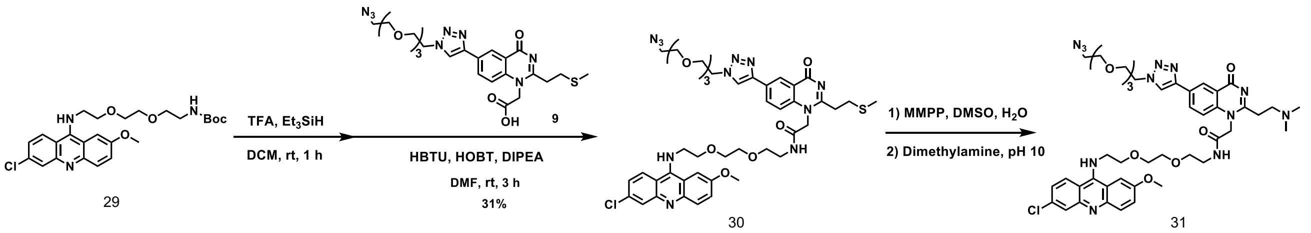

9-((2-(2-(2-(2-(6-(1-(2-(2-(2-(2-azidoethoxy)ethoxy)ethoxy)ethyl)-1H-1,2,3-triazol-4-yl)-2-(2-(methylthio)ethyl)-4-oxoquinazolin-1(4H)-yl)acetamido)ethoxy)ethoxy)ethyl)amino)-6-chloro-2-methoxyacridin-10-ium (30)

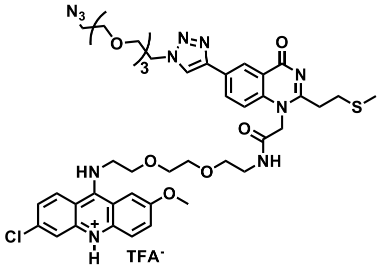

To a 10 mL round-bottomed flask was added compound (**29**) (5.0 mg, 10 μmol) dissolved 200 μL of DCM, followed by addition of triethylsilane (8.0 μL, 51 μmol) and TFA (400 μL). After stirring 1 h, the reaction solution was evaporated with MeCN three times. The residue was added to a mixture of compound (**9**) (5.1 mg, 9.3 μmol), DIPEA (4.8 μL), HBTU (5.3 mg, 13 μmol) and HOBt (1.8 mg, 13 μmol) in DMF (500 μL). The reaction solution was stirred at r.t. for 2 h, and diluted with EtOAc. The solution was extracted with EtOAc/$NaHCO_3$aq three times and washed with brine. The organic layer was dried over with Na_2SO_4 and evaporated under reduced pressure. The crude material was through amino silica pad and purified by HPLC. The target compound (**30**) was obtained as a yellow solid in 31% yield. HPLC conditions: A: 0.1% TFA in distilled water, B: 0.1% TFA in MeCN, B: 10%→50% (20 min); Flow rate = 1 mL/min; Temp. = 40 °C; UV = 254 nm, C-18 column (Nacalai tesque: COSMOSIL 5C18-AR-II, 4.5×250 mm).

^{1}H NMR (600 MHz, DMSO-d_6) δ (ppm) 13.50 (s, 1H), 9.47 (s, 1H), 8.68 (s, 1H), 8.58-8.60 (m, 2H), 8.44 (J = 2.4 Hz, 1H), 8.19 (dd, J = 1.8, 8.4 Hz, 1H), 7.93 (s, 1H), 7.77 (d, J = 9.6 Hz, 2H), 7.70 (dd, J = 2.4, 9.6 Hz, 1H), 7.51-7.54 (m, 2H) 4.95 (s, 2H), 4.60 (t, J = 5.4 Hz, 2H), 4.29 (d, J = 4.2 Hz, 2H), 3.98 (s, 3H), 3.91-3.99 (m, 4H), 3.55-3.68 (m, 12H), 3.53 (s, 4H), 3.42 (t, J = 5.4 Hz, 2H), 3.26 (q, J = 5.4 Hz, 2H), 3.08 (s, 2H), 2.91 (t, J = 7.2 Hz, 2H), 2.15 (s, 3H).

^{13}C NMR (150 MHz, CD_3OD) δ (ppm) 170.1, 168.0, 165.3, 162.7, 158.2, 146.4, 142.0, 141.8, 132.2, 130.0, 128.8, 125.1, 124.7, 123.5, 121.4, 120.7, 118.4, 117.5, 71.9, 71.6, 71.5, 71.5, 71.4, 71.0, 70.6, 70.2, 56.7, 51.7, 50.6, 50.5, 40.4, 36.2, 31.7, 31.6, 15.6.

HRMS (ESI-TOF) (*m/z*) calcd. for $C_{43}H_{53}ClN_{11}O_8S^+$ $[M+H]^+$ 918.3482, found 918.3422.

9-((2-(2-(2-(2-(6-(1-(2-(2-(2-(2-azidoethoxy)ethoxy)ethoxy)ethyl)-1H-1,2,3-triazol-4-yl)-2-(2-(dimethylamino)ethyl)-4-oxoquinazolin-1(4H)-yl)acetamido)ethoxy)ethoxy)ethyl)amino)-6-chloro-2-methoxyacridin-10-ium (**31**)

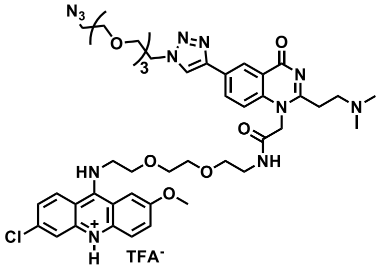

To a solution of compound (**30**) (210 nmol) in DMSO (15 μL), MMPP (126 μL, 630 μmol, 5 mM in water) and DMSO (70 μL) were added, the mixture was stirred at room temperature for 1 min. Then, carbonate buffer (pH = 10, 42 μL) and dimethylamine (21 μL, 11 μmol, 500 mM in H_2O) were added, and the mixture was incubated at 37 °C for 30 min. The solution was purified by HPLC to afford compound (**31**) (54 μmol, 26%).

HRMS (ESI-TOF) (*m/z*) calcd. for $C_{44}H_{56}ClN_{12}O_8^+$ $[M+H]^+$ 915.4027, found 915.4004.

Compound-**2**-^{1}H [Acetone-d_6]

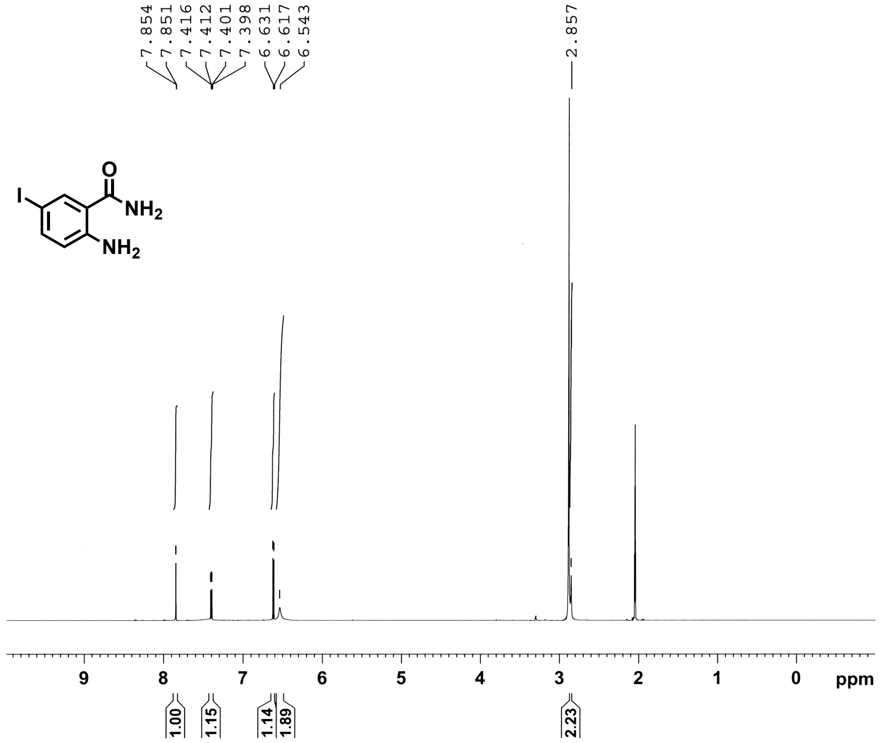

Compound-**2**-^{13}C [Acetone-d_6]

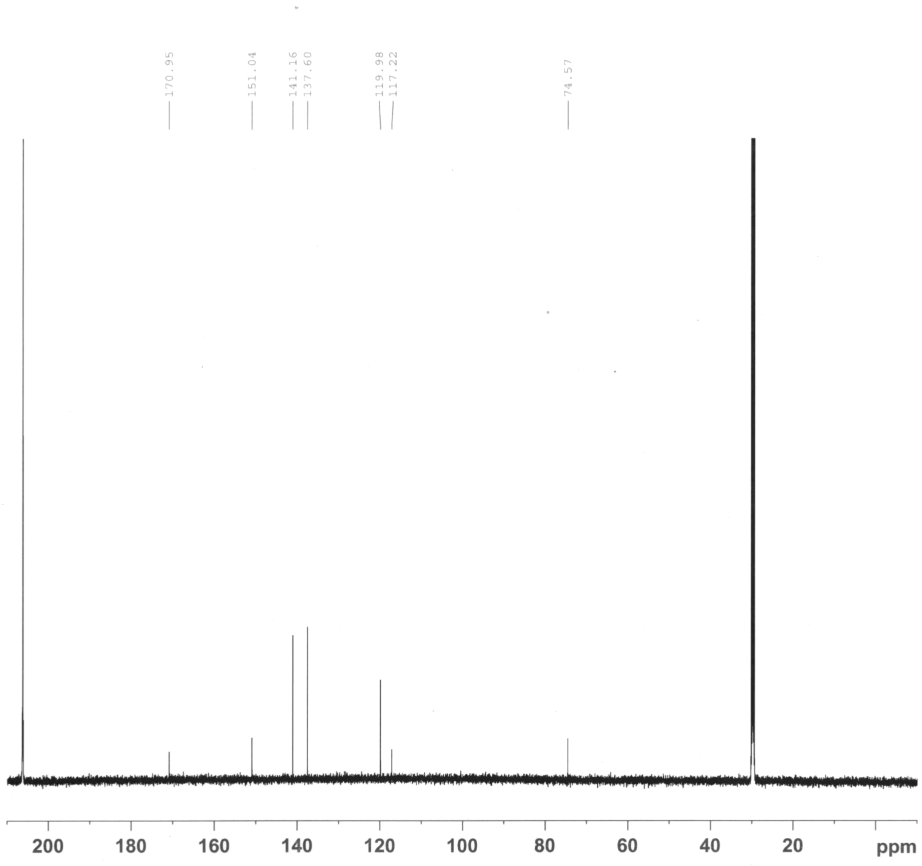

Compound-**3**-[1]H [$CDCl_3$]

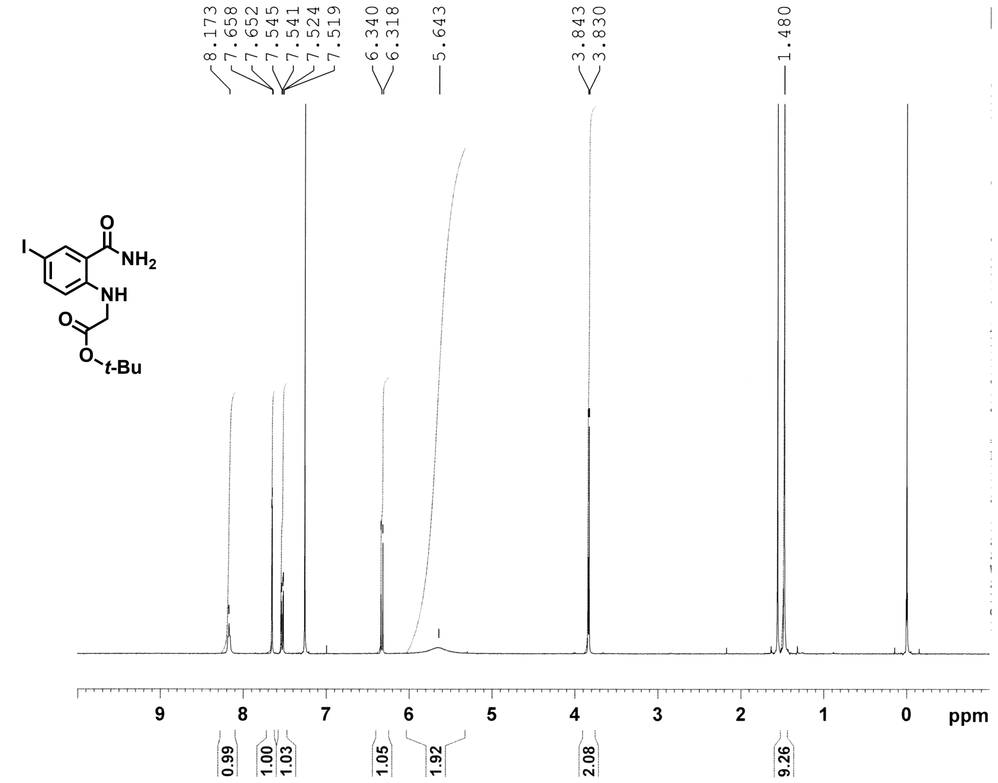

Compound-**3**-[13]C [DMSO-*d*6]

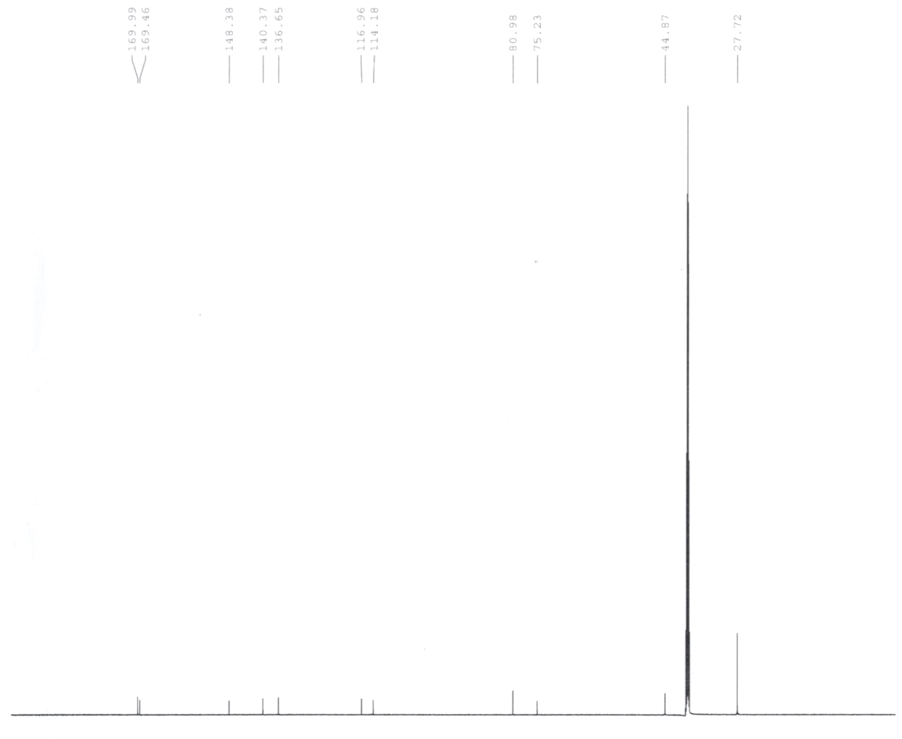

Compound-**4**-[1]H [$CDCl_3$]

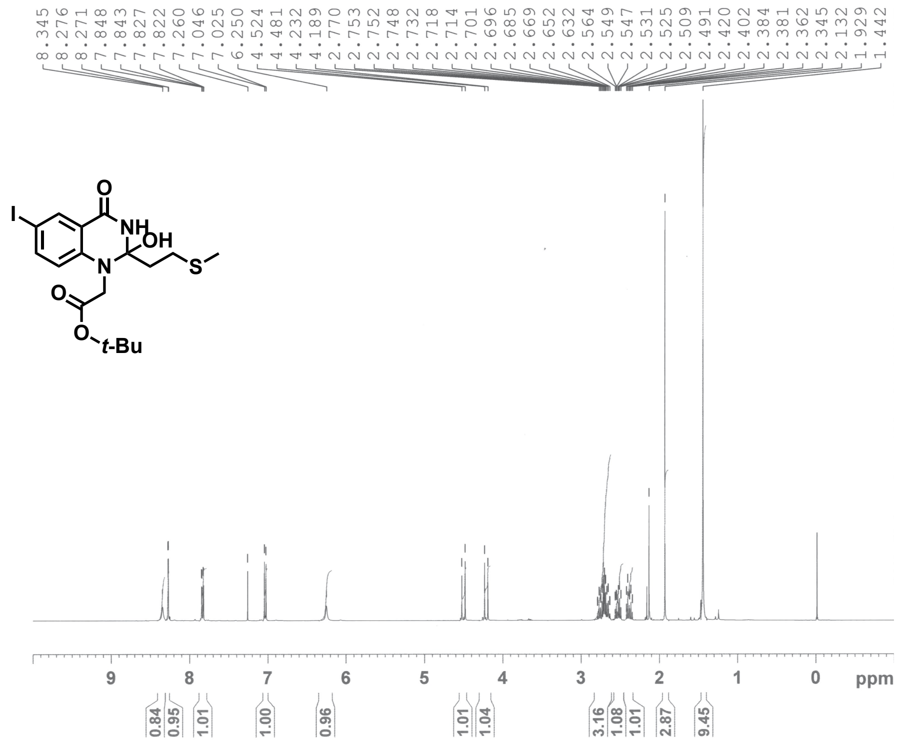

Compound-**4**-^{13}C [$CDCl_3$]

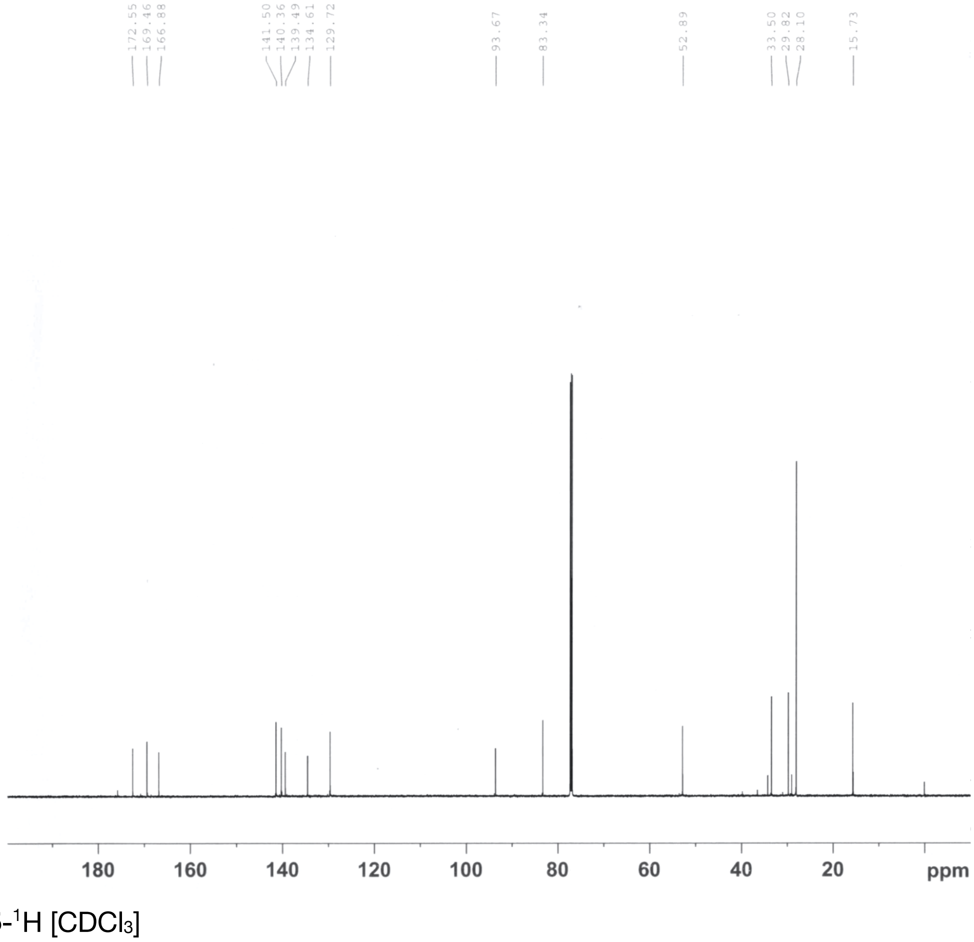

Compound-**5**-^{1}H [$CDCl_3$]

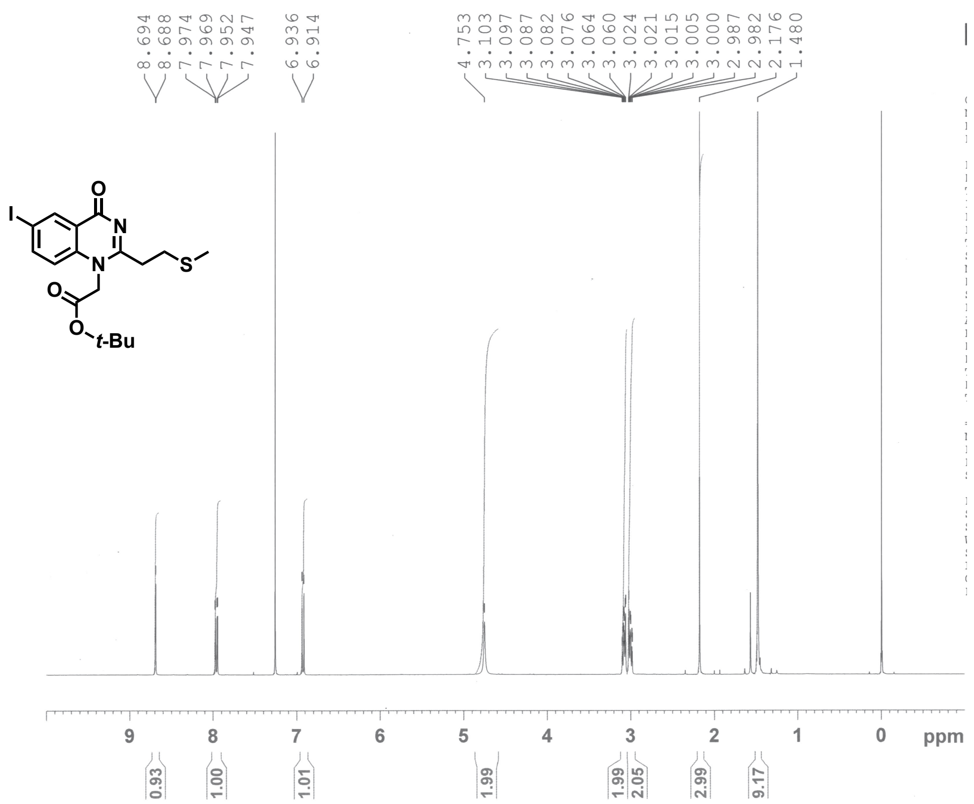

Compound-**5**-^{13}C [CDCl$_3$]

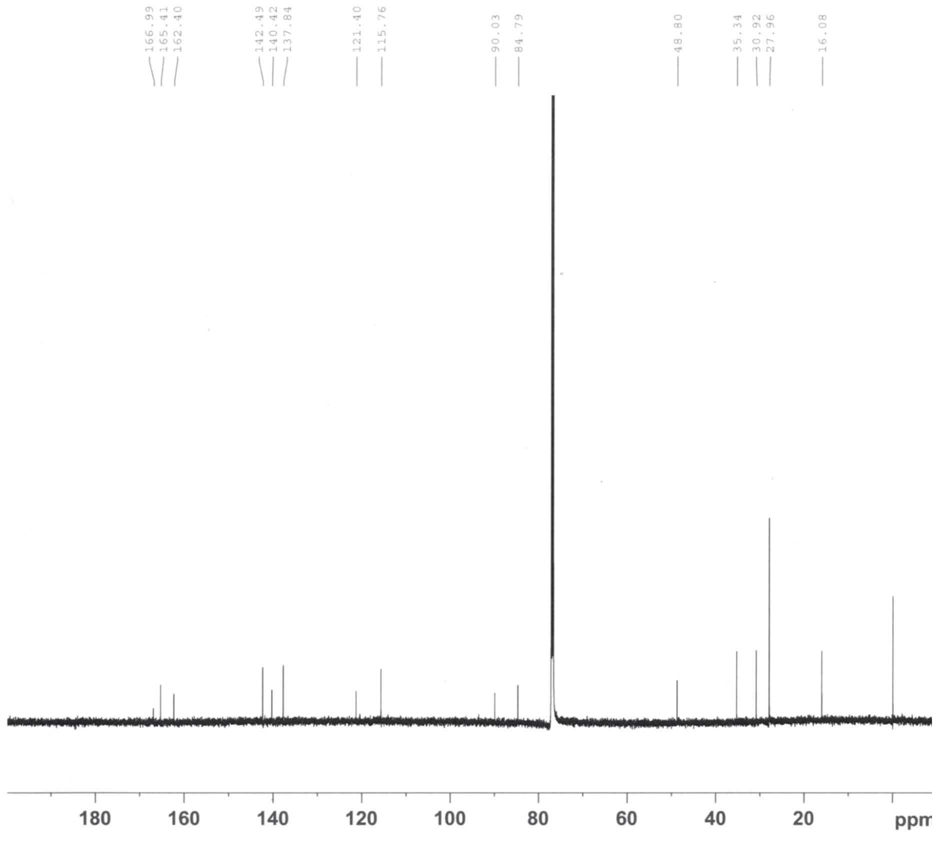

Compound-**6**-^{1}H [CDCl$_3$]

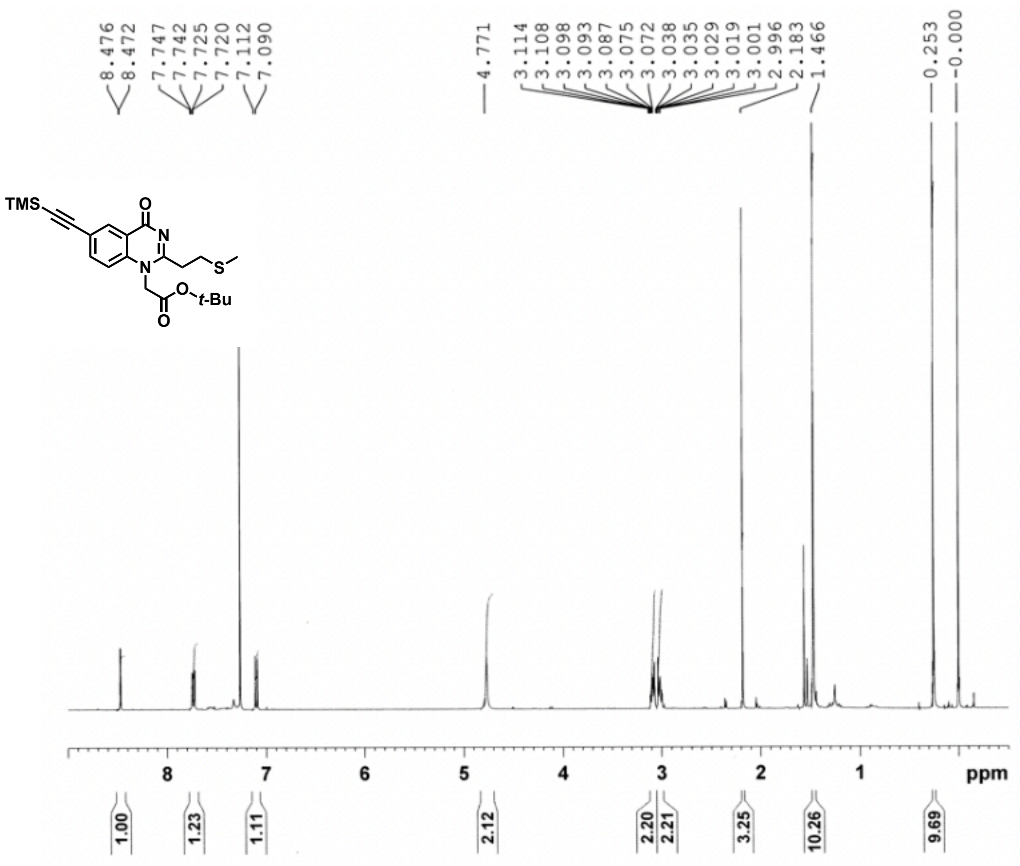

Compound-**6**-^{13}C [$CDCl_3$]

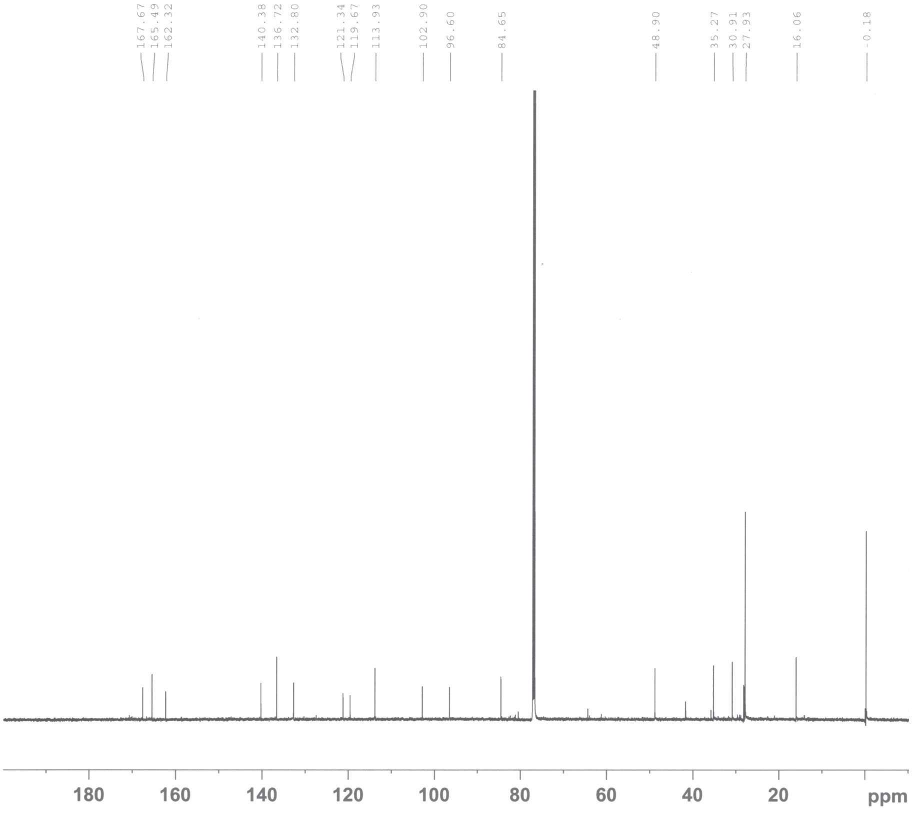

Compound-**7**-^{1}H [$CDCl_3$]

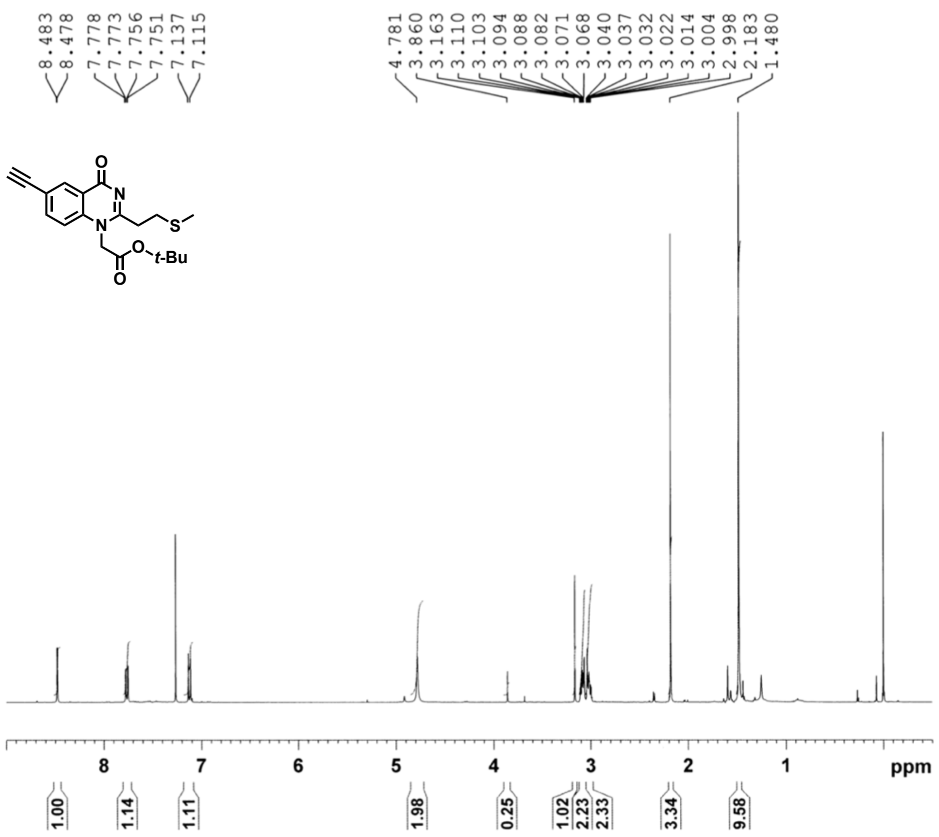

Compound-**7**-^{13}C [$CDCl_3$]

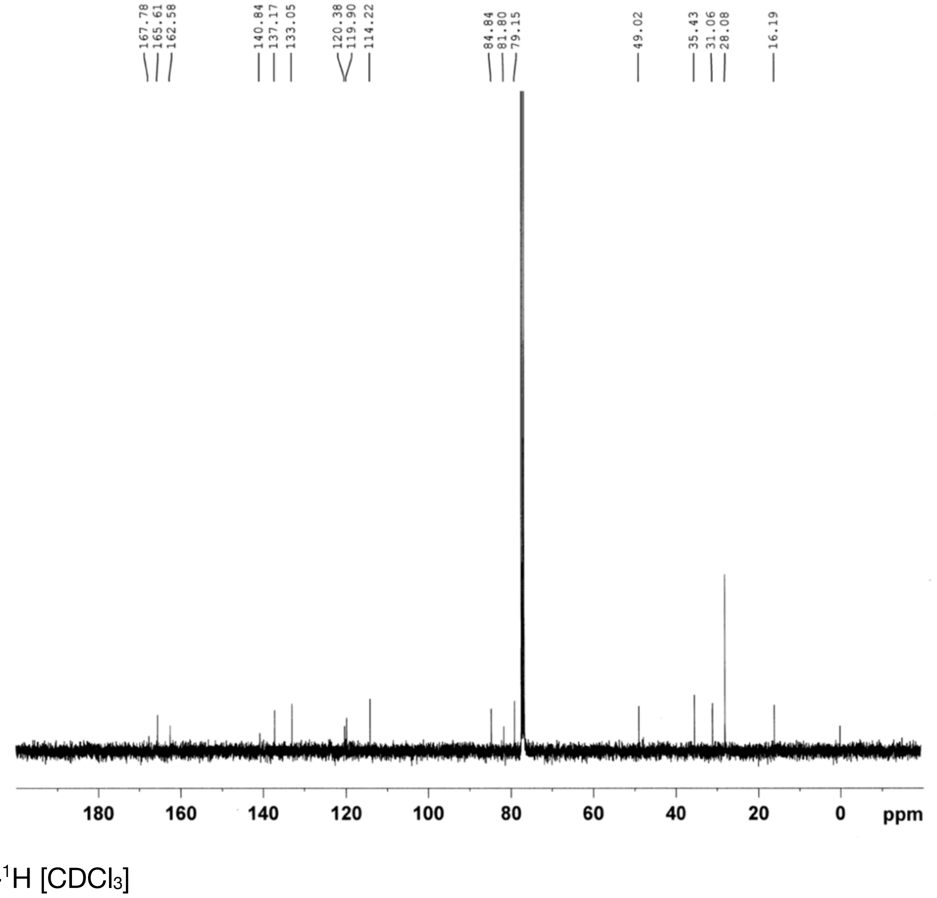

Compound-**8**-^{1}H [$CDCl_3$]

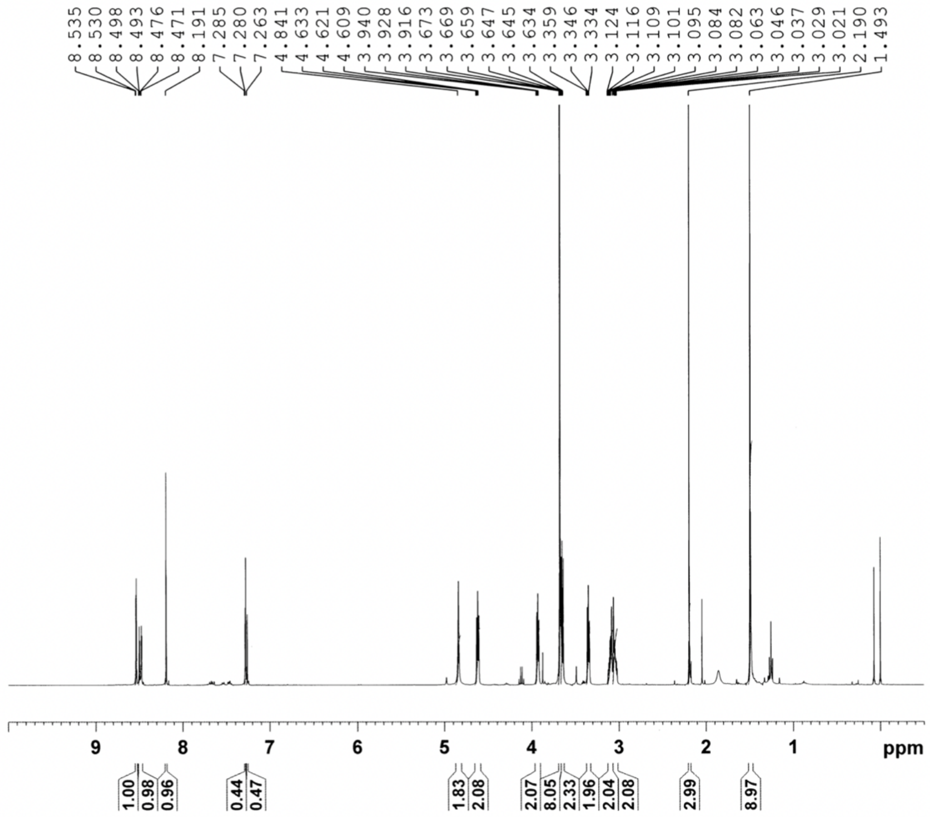

Compound-**8**-^{13}C [$CDCl_3$]

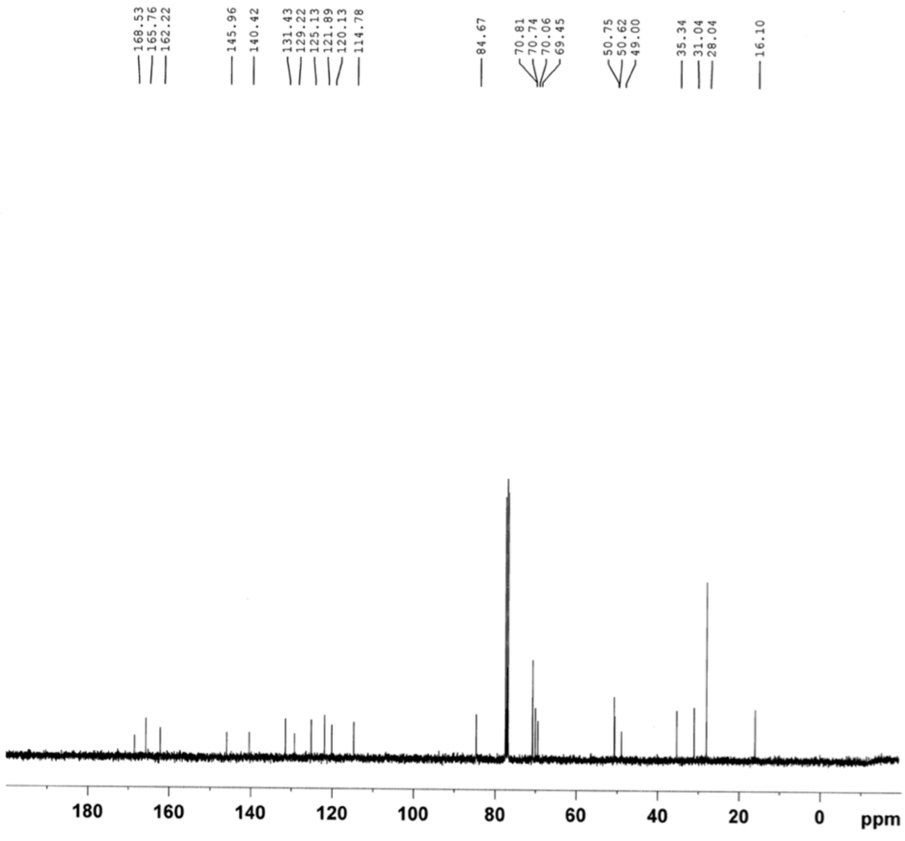

Compound-**15**-^{1}H [DMSO-d_6]

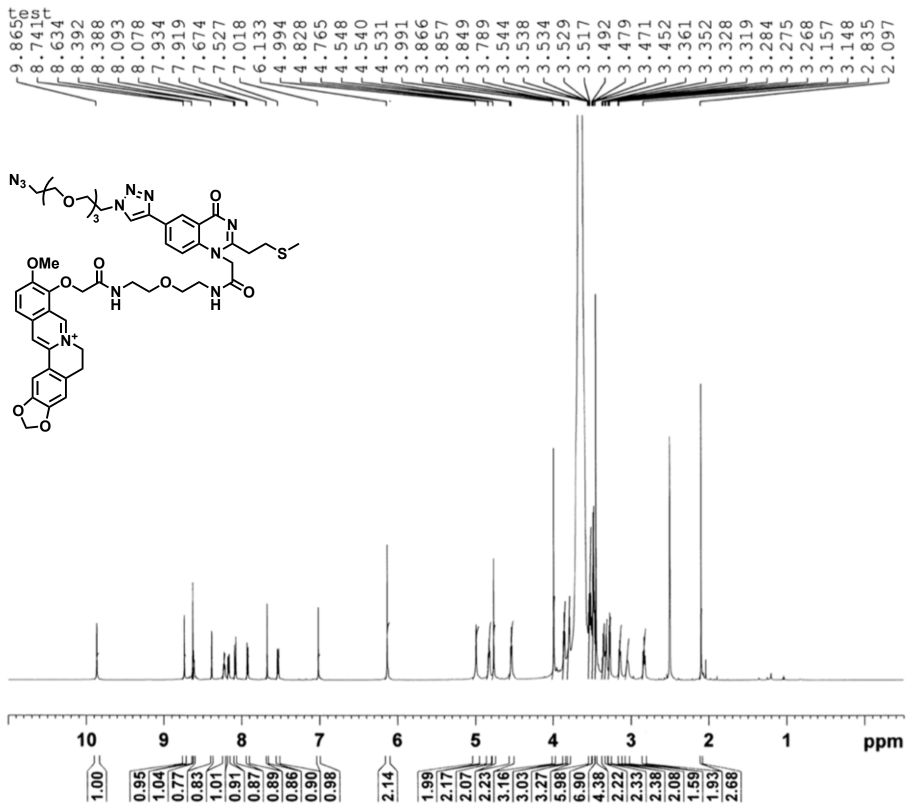

Compound-**15**-^{13}C [DMSO-d_6]

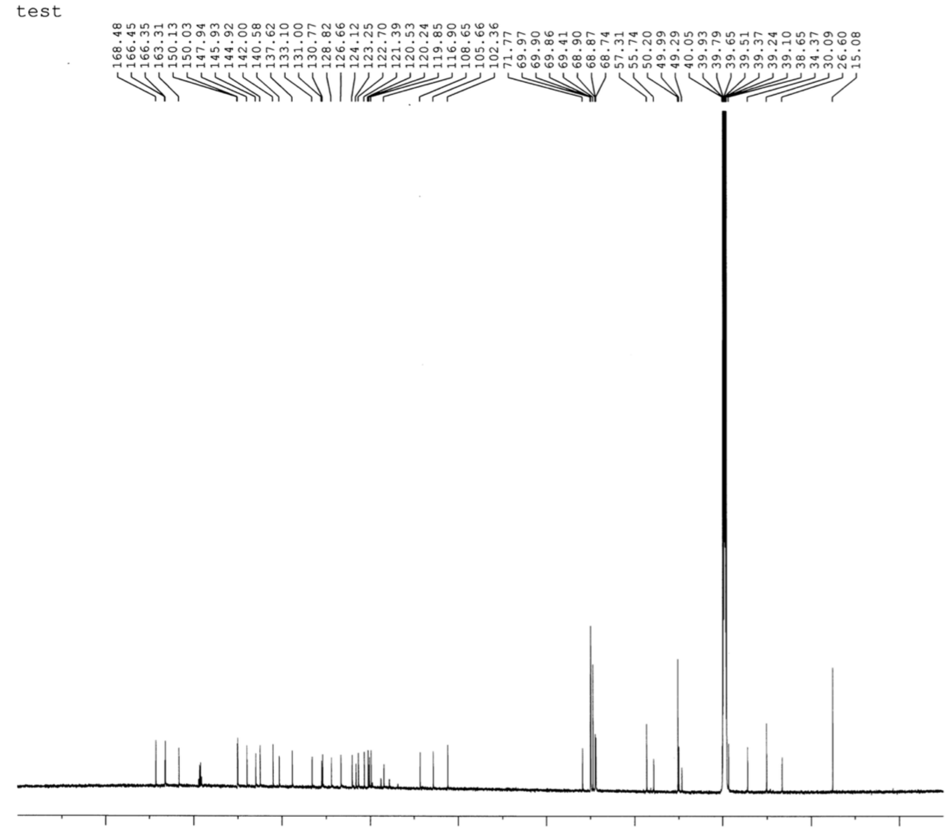

Compound-**16**-^{1}H [DMSO-d_6]

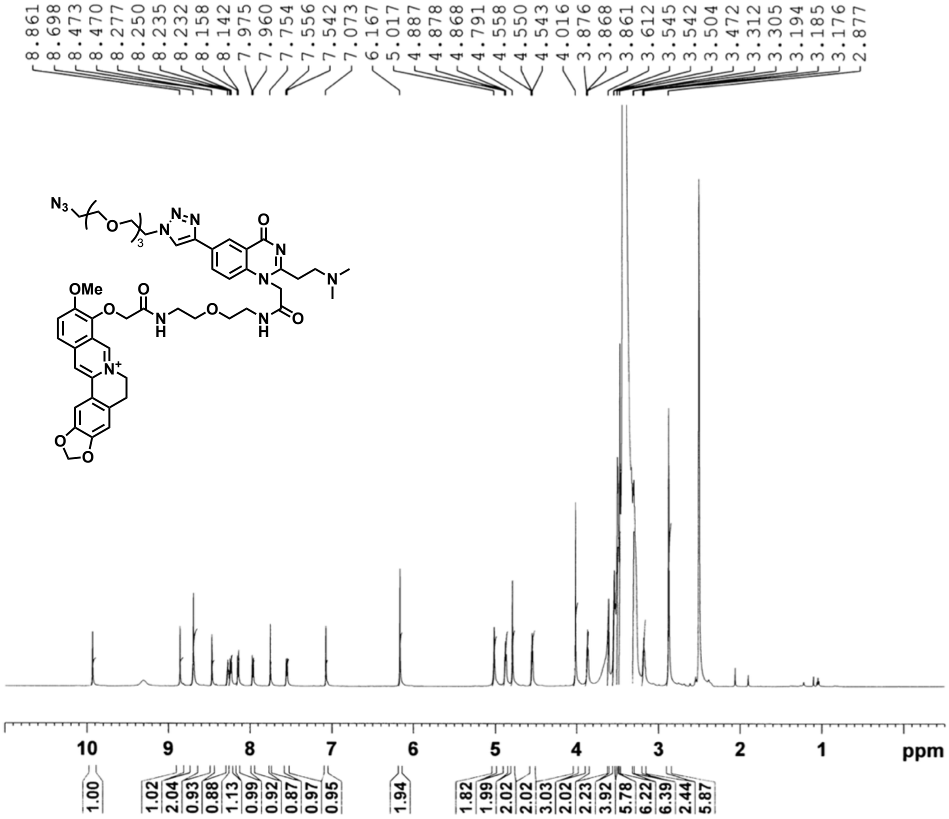

Compound-**16**-13C [DMSO-d_6]

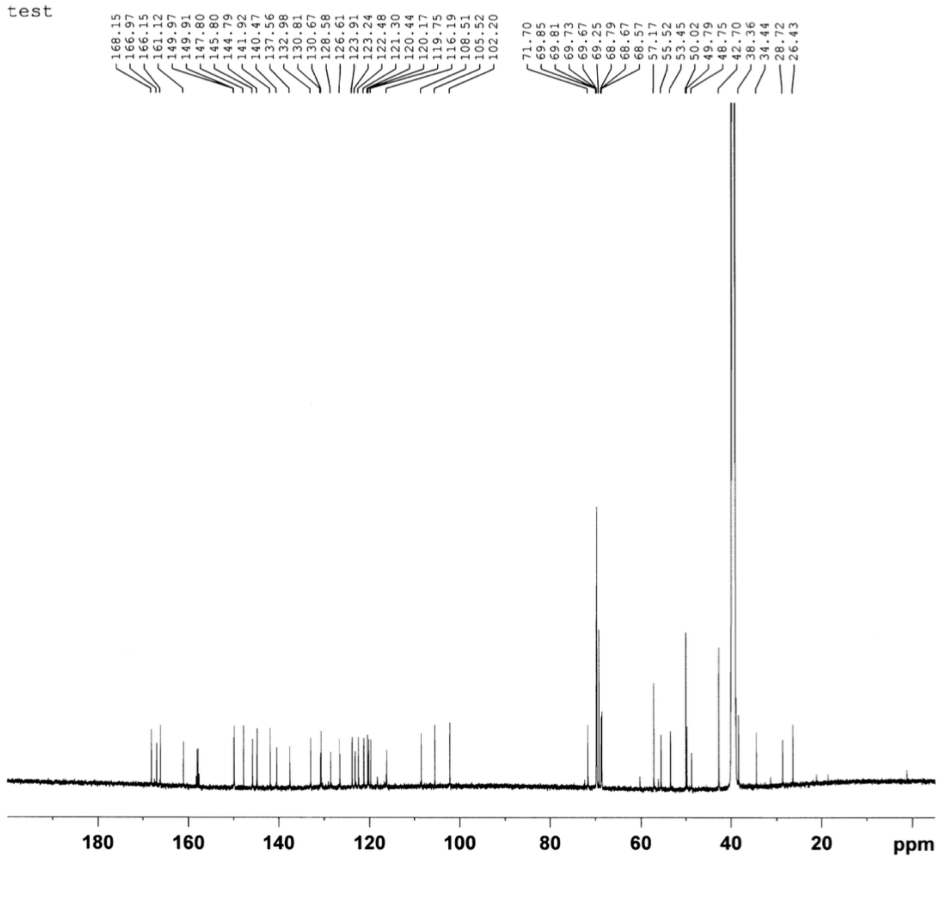

Compound-**19**-^{1}H [DMSO-d_6]

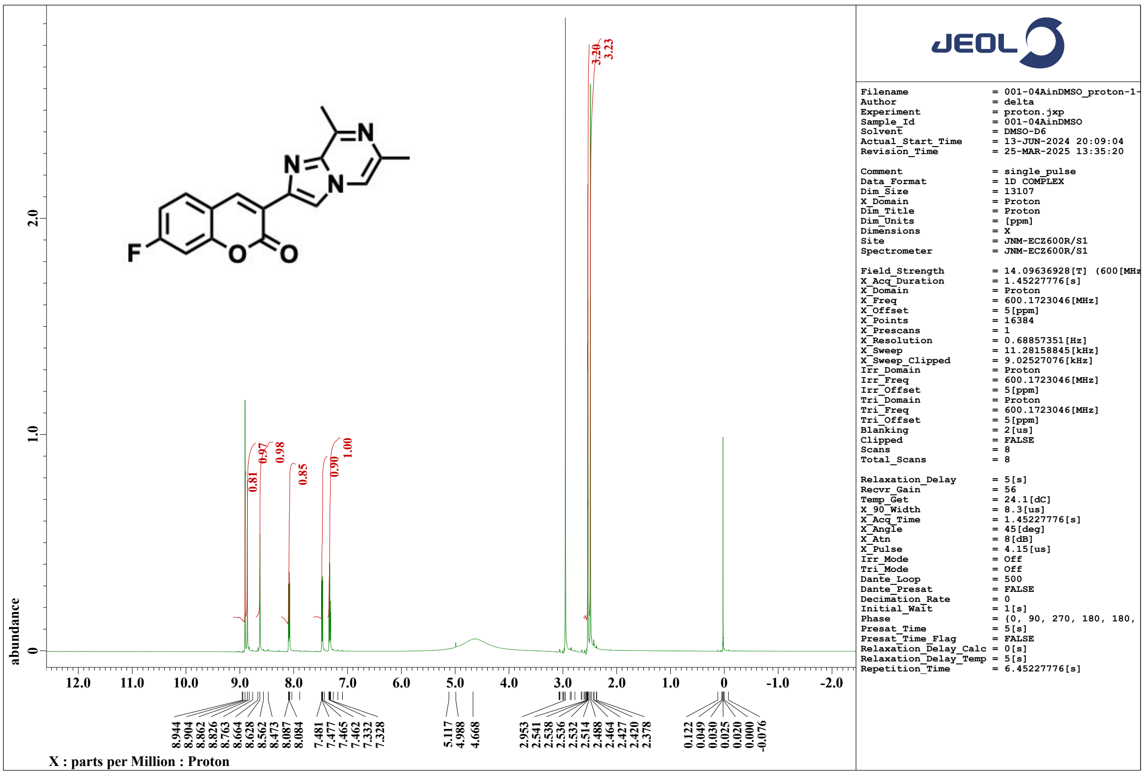

Compound-**19**-^{13}C [DMSO-d_6]

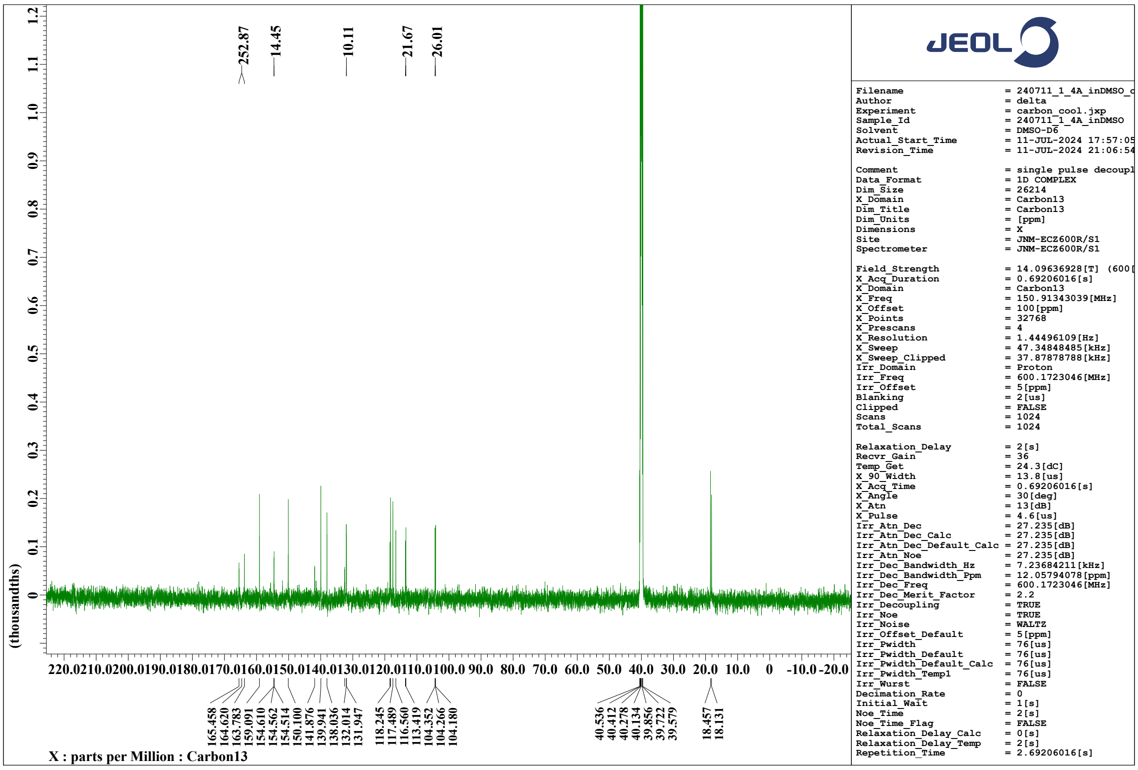

Compound-**20**-^{1}H [DMSO-d_6]

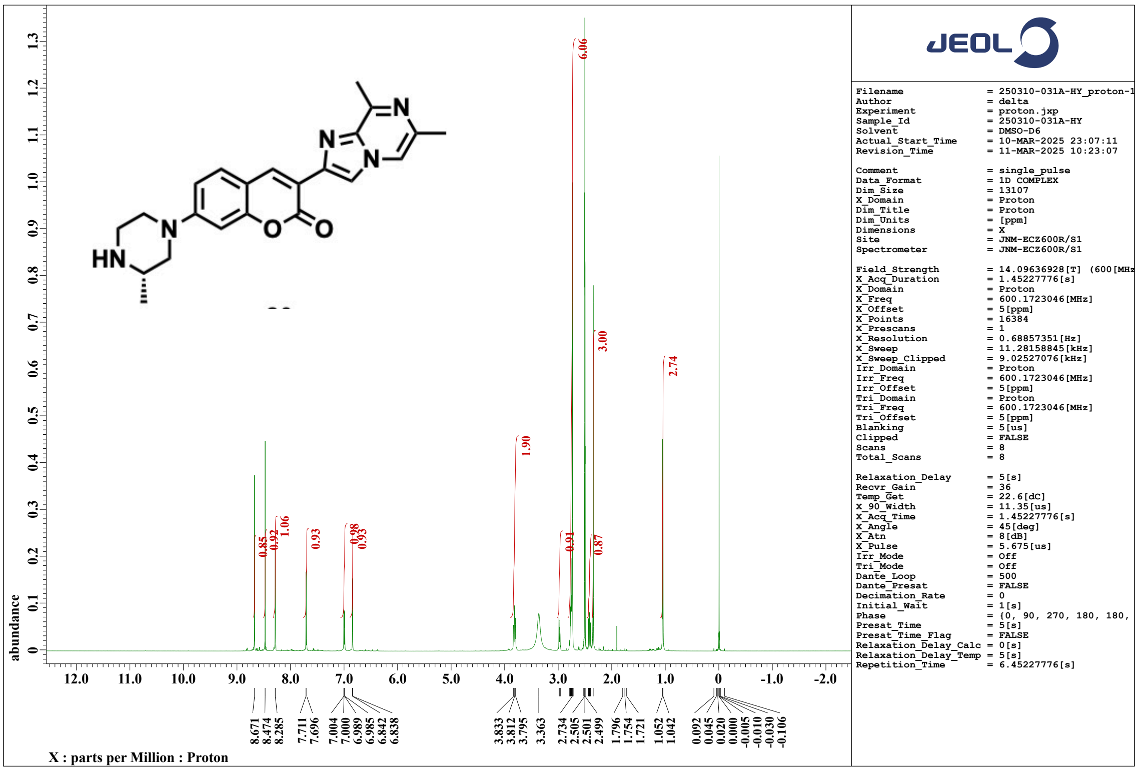

Compound-**20**-^{13}C [DMSO-d_6]

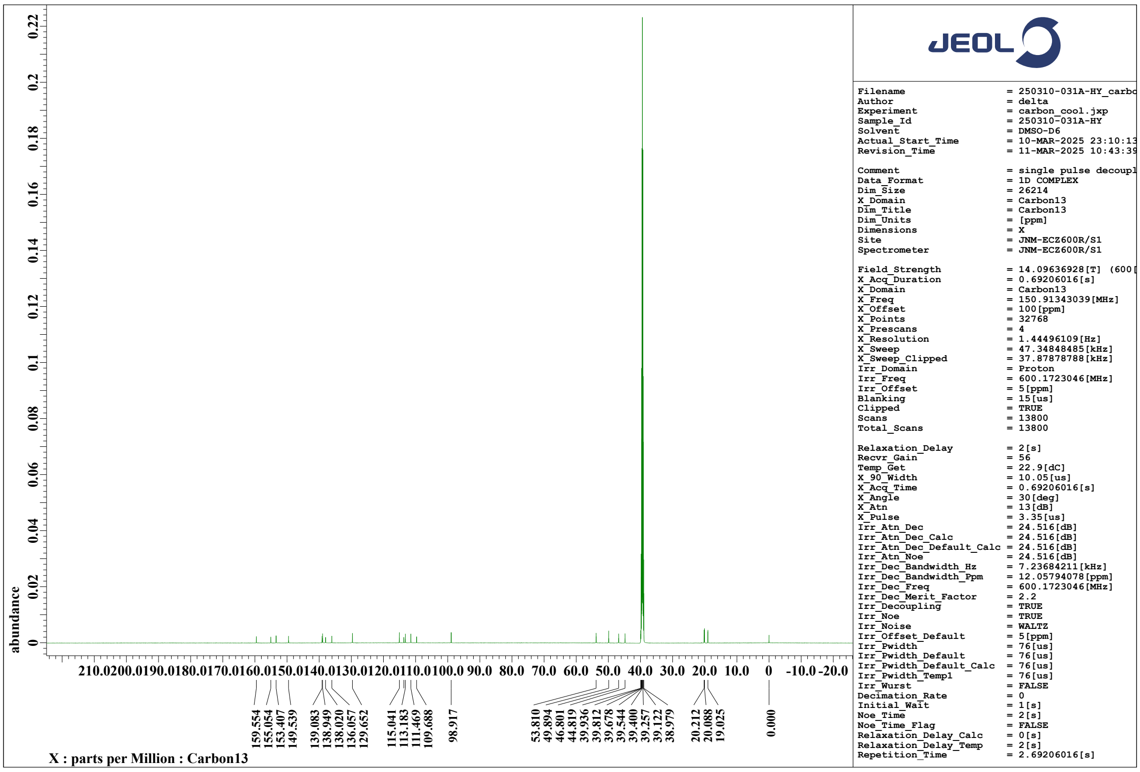

Compound-**21**-^{1}H [DMSO-d_6]

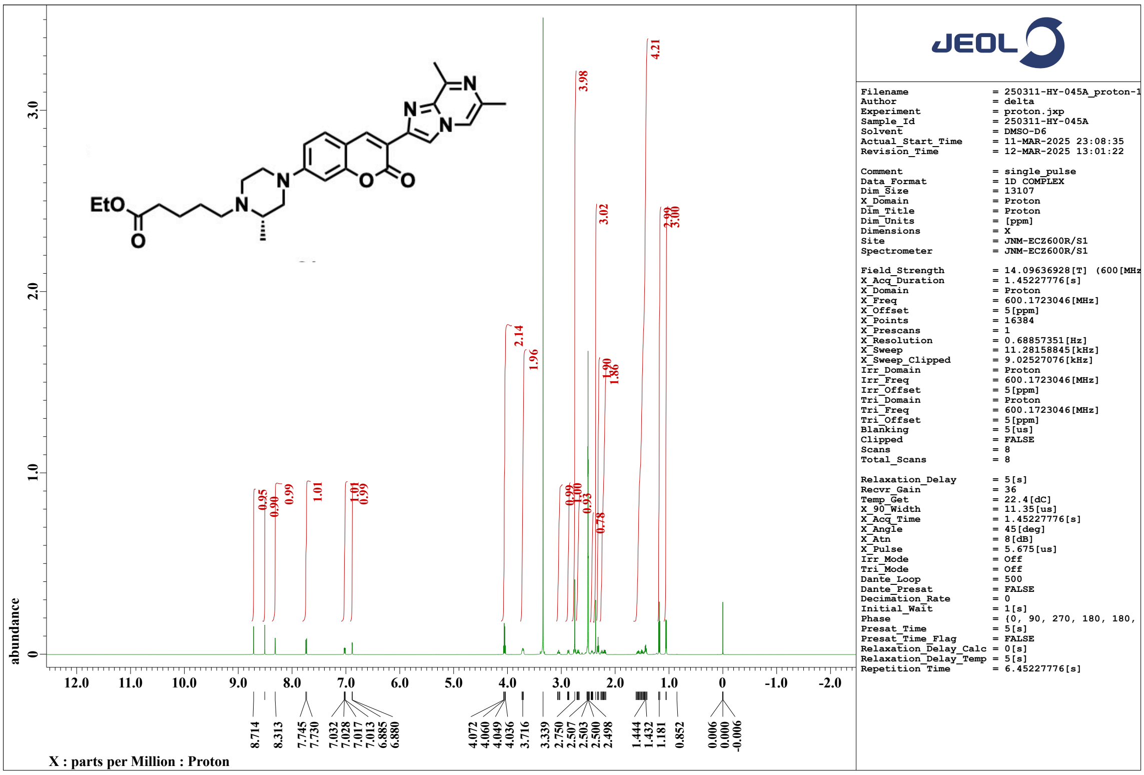

Compound-**21**-^{13}C [DMSO-d_6]

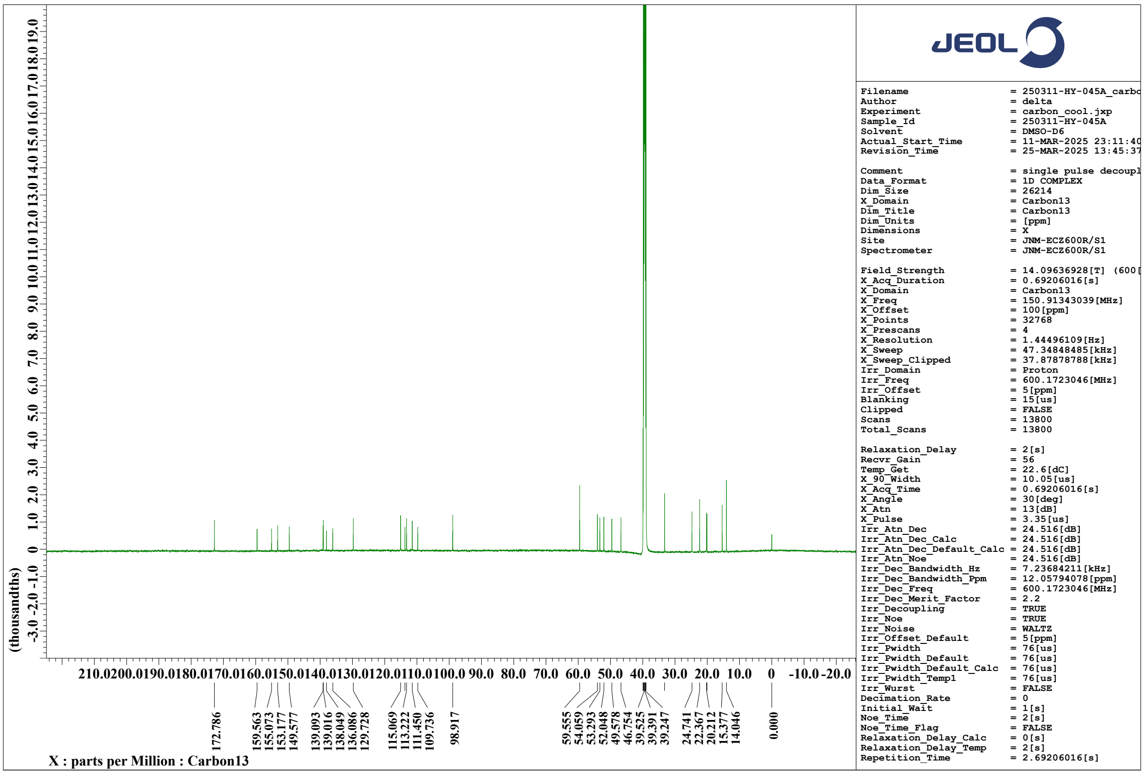

Compound-**22**-^{1}H [DMSO-d_6]

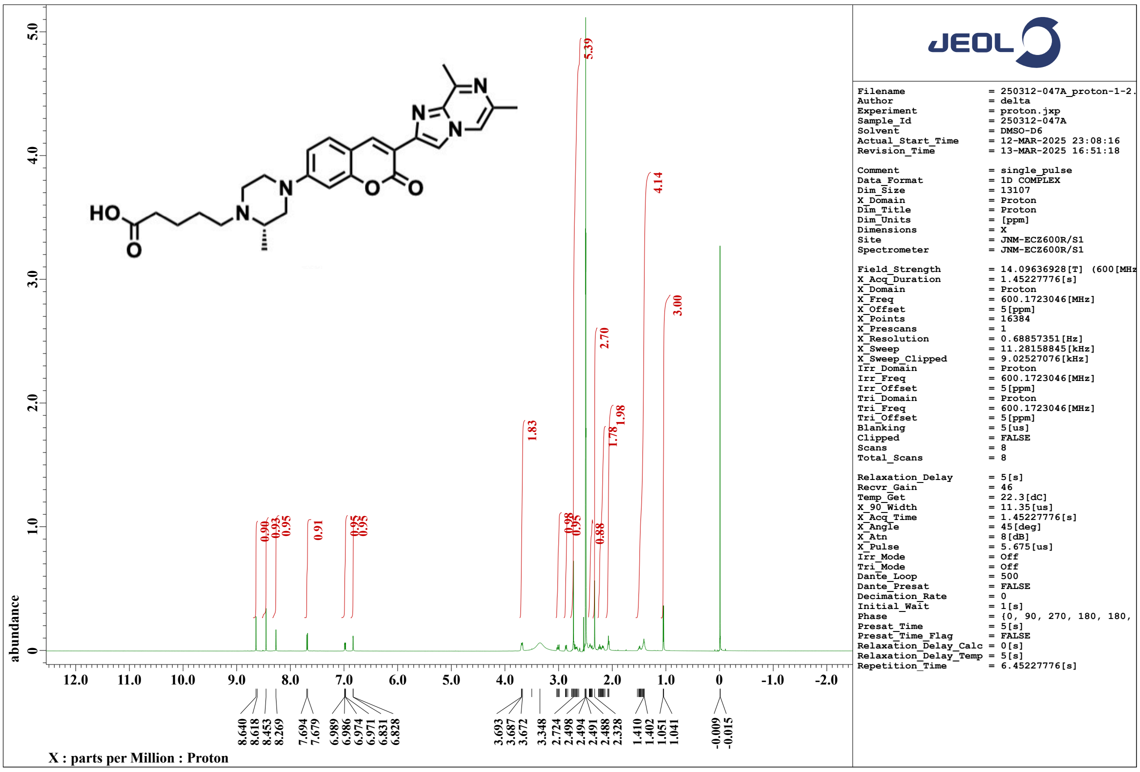

Compound-**22**-^{13}C [DMSO-d_6]

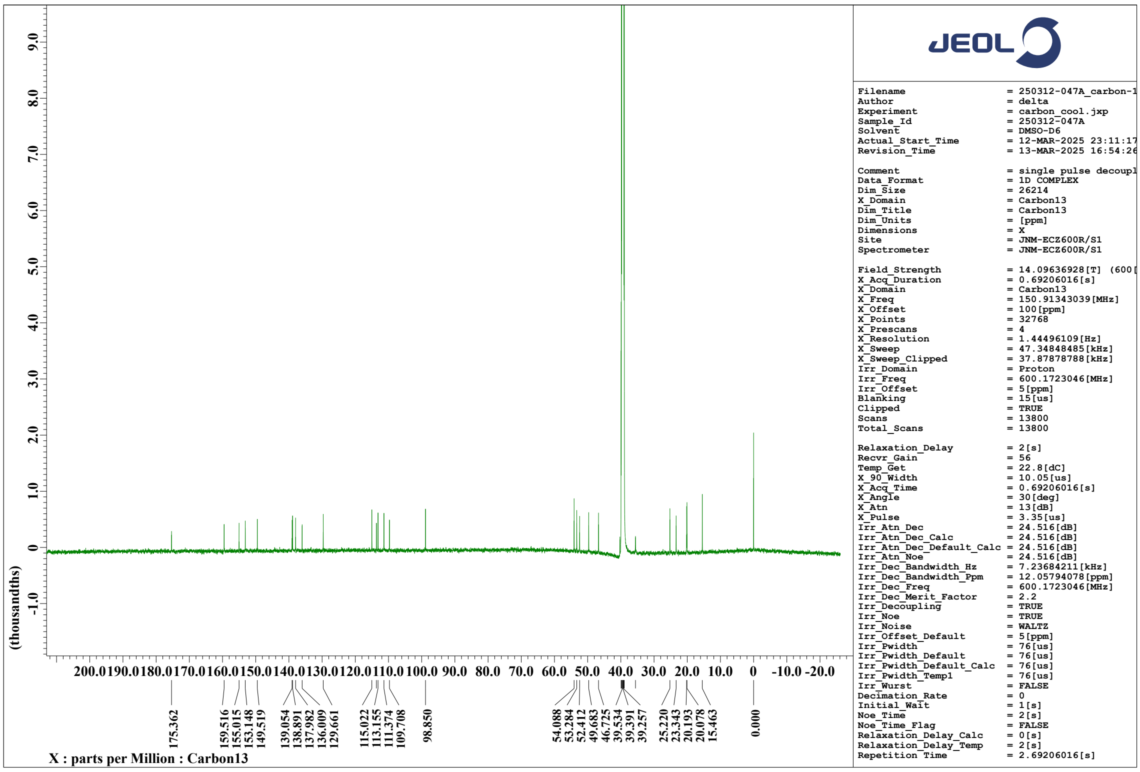

Compound-**23**-^{1}H [DMSO-*d*$_6$]

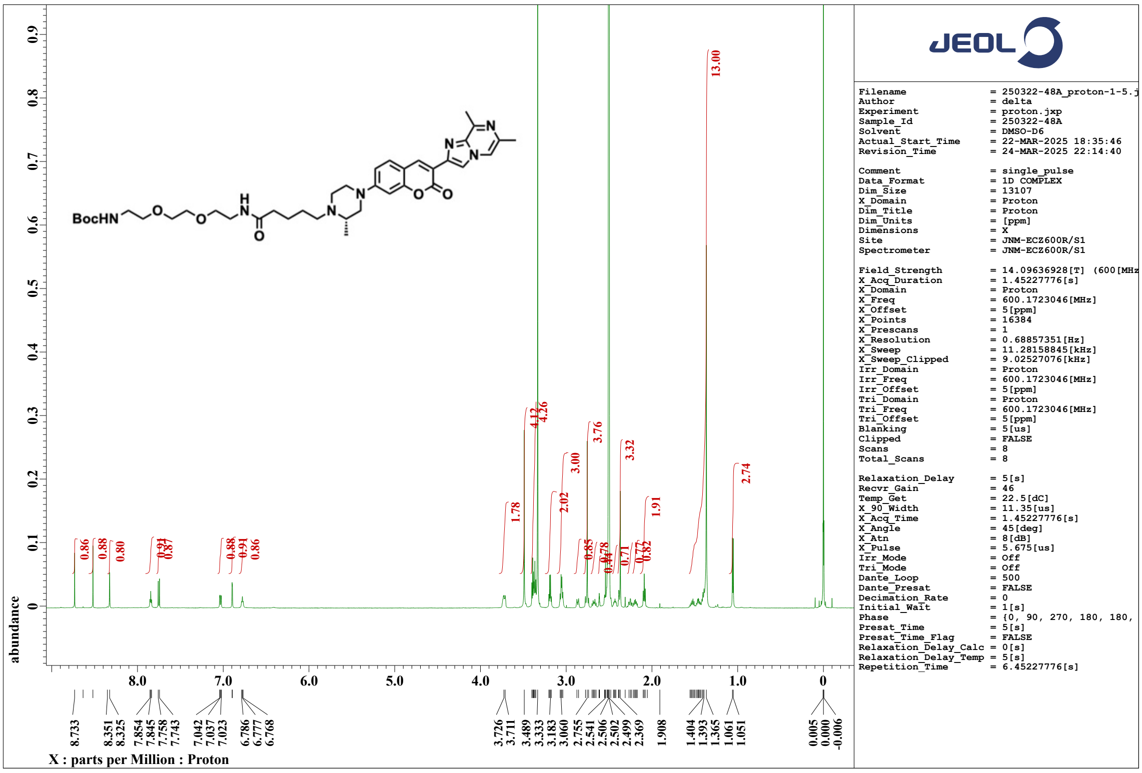

Compound-**23**-^{13}C [DMSO-*d*$_6$]

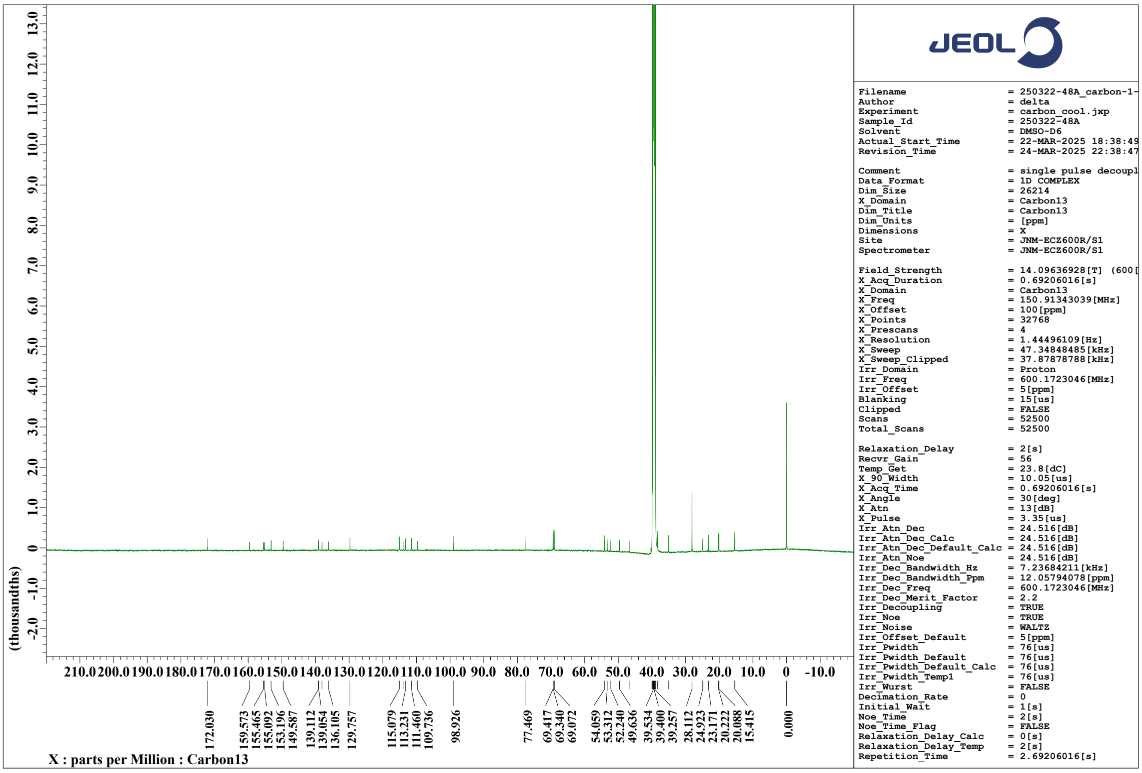

Compound-**24**-^{1}H [DMSO-d_6]

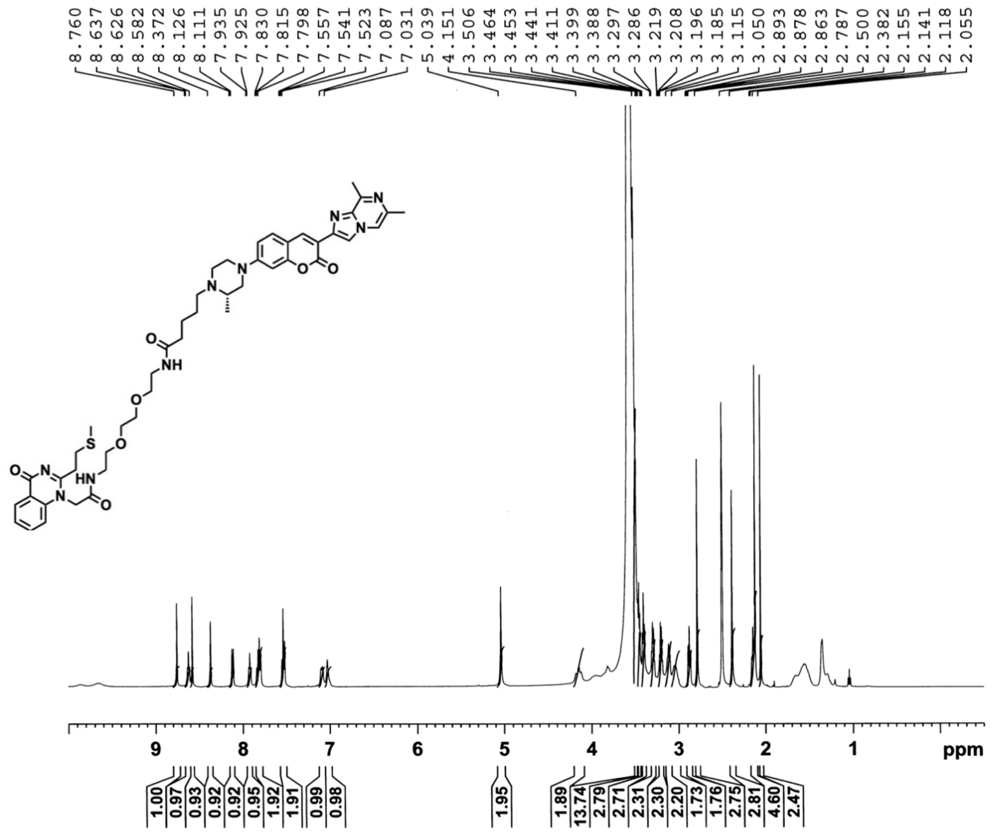

Compound-**24**-^{13}C [DMSO-d_6]

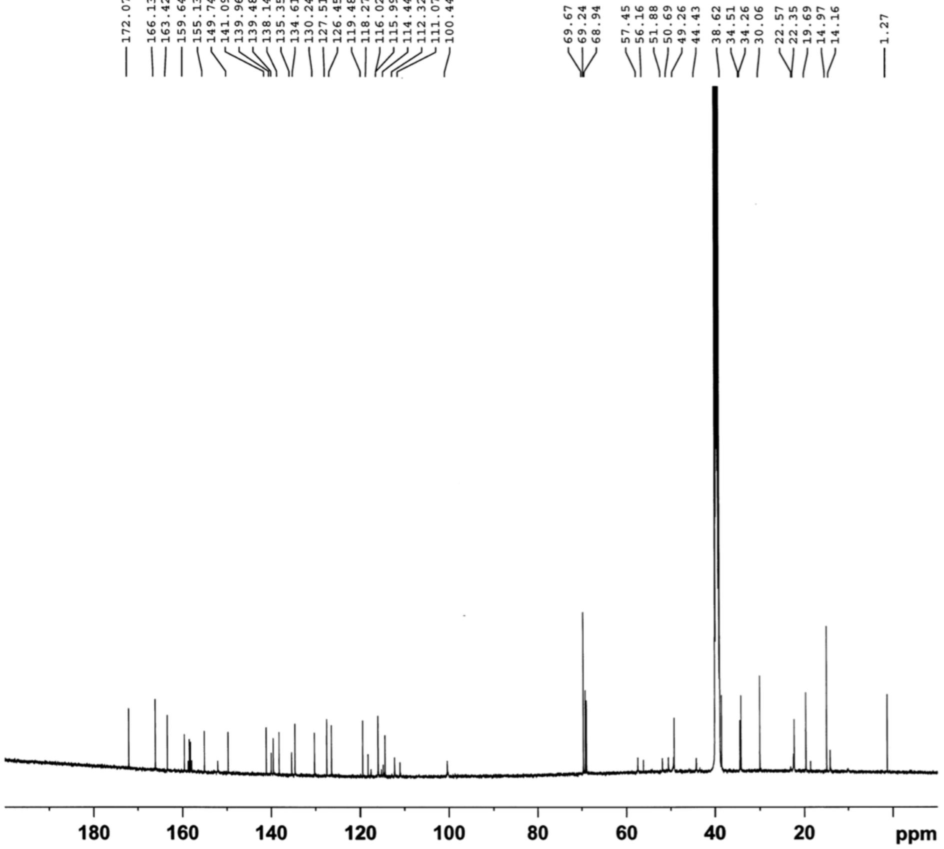

Compound-**25**-^{1}H [DMSO-d_6]

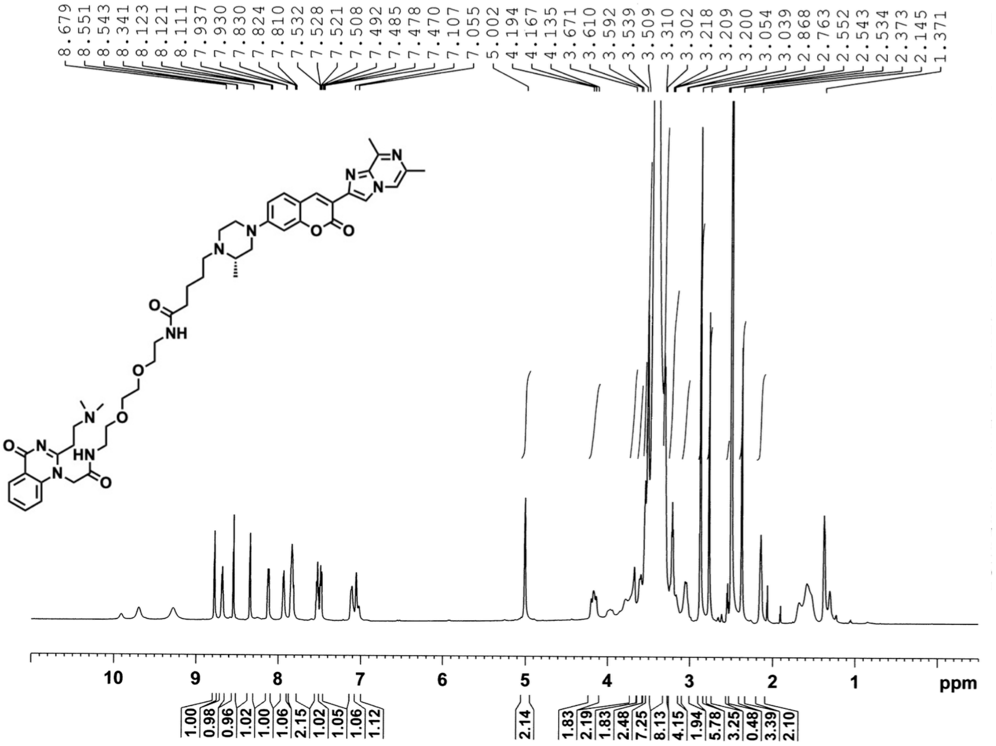

Compound-**25**-^{13}C [DMSO-d_6]

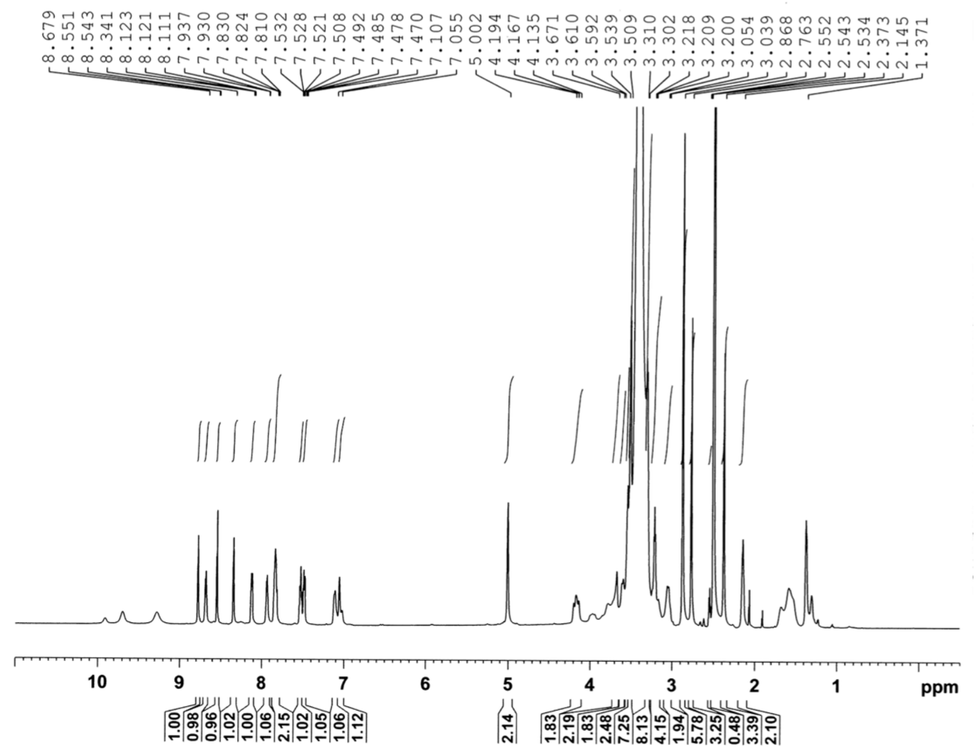

Compound-**27**-^{1}H [DMSO-d_6]

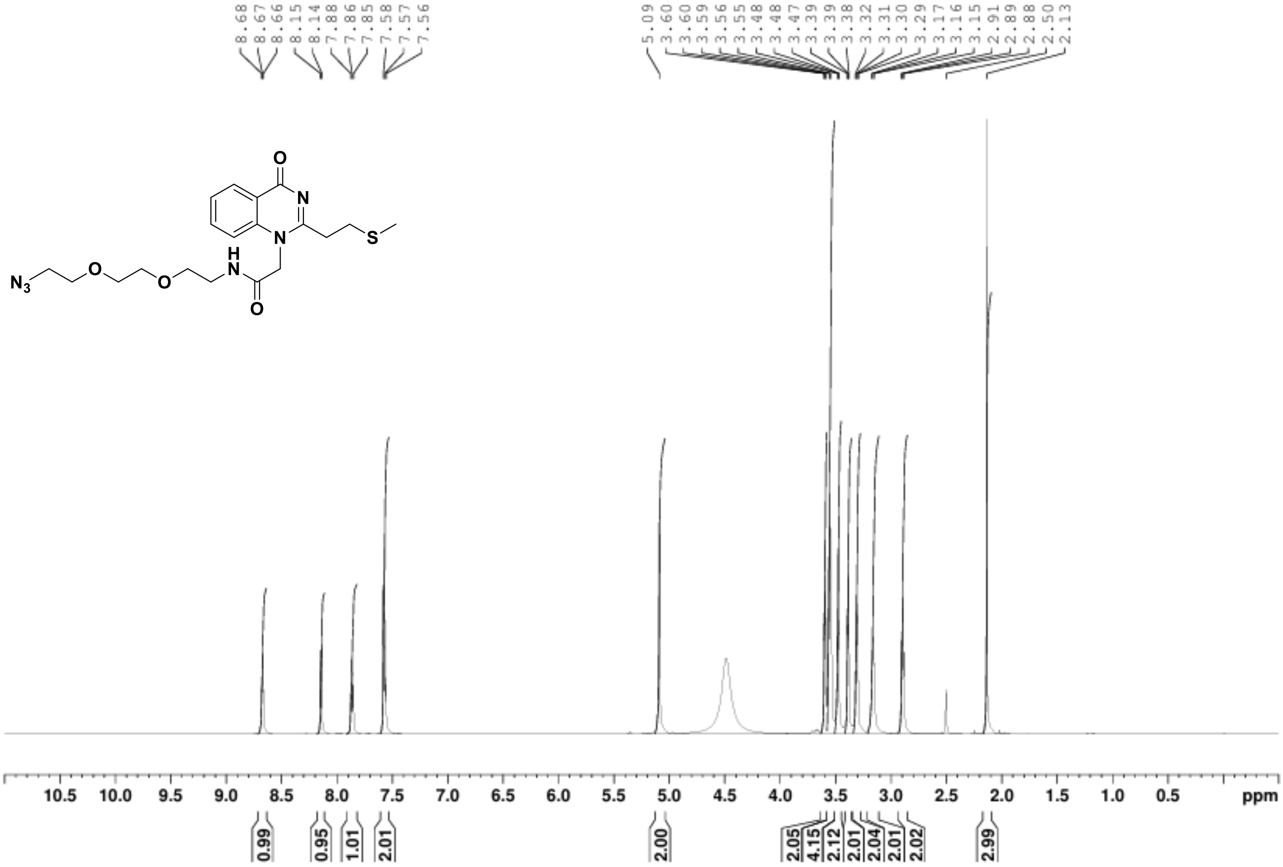

Compound-**27**-^{13}C [DMSO-d_6]

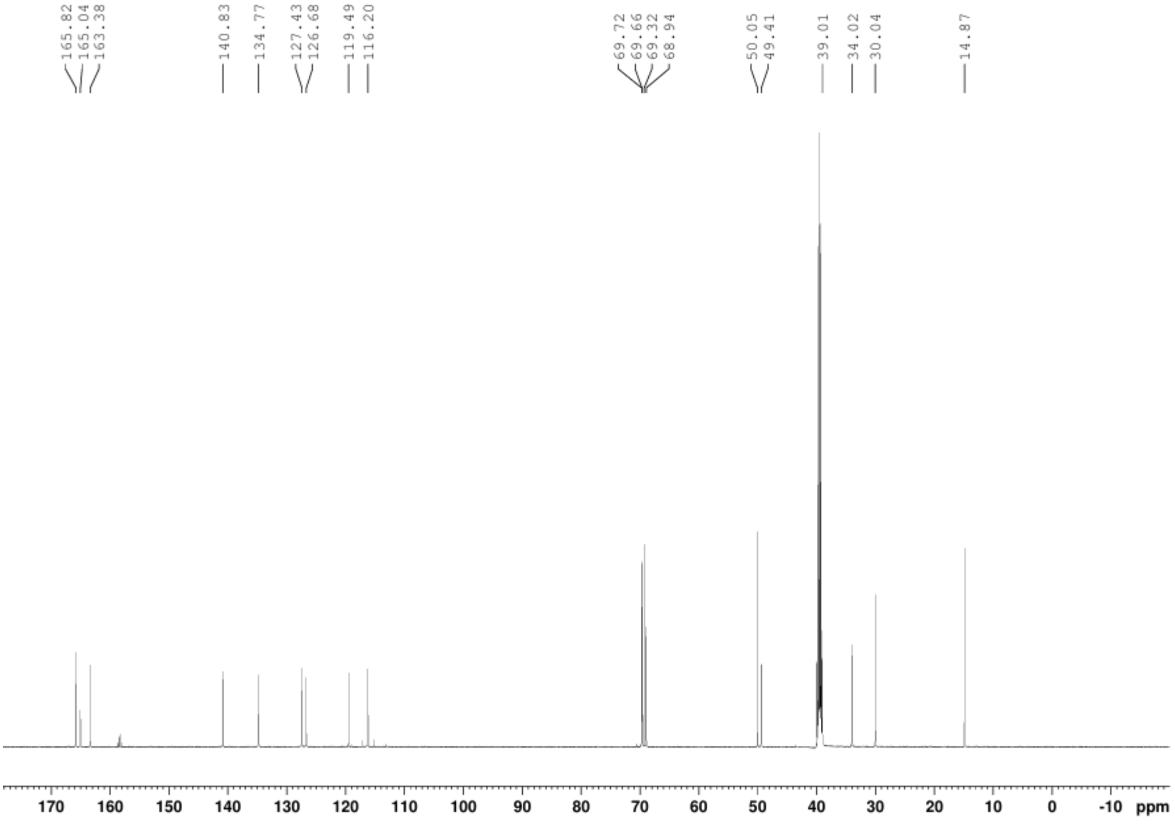

Compound-**28**-^{1}H [DMSO-d_6]

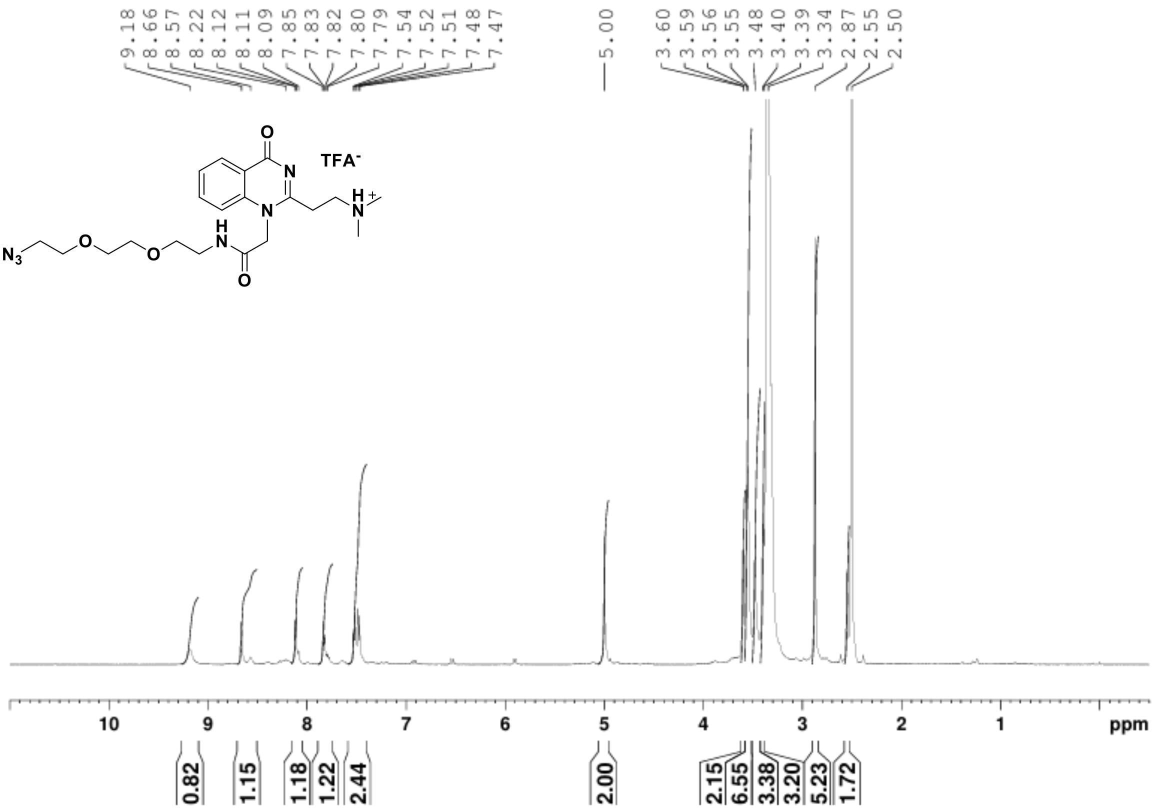

Compound-**28**-^{13}C [DMSO-d_6]

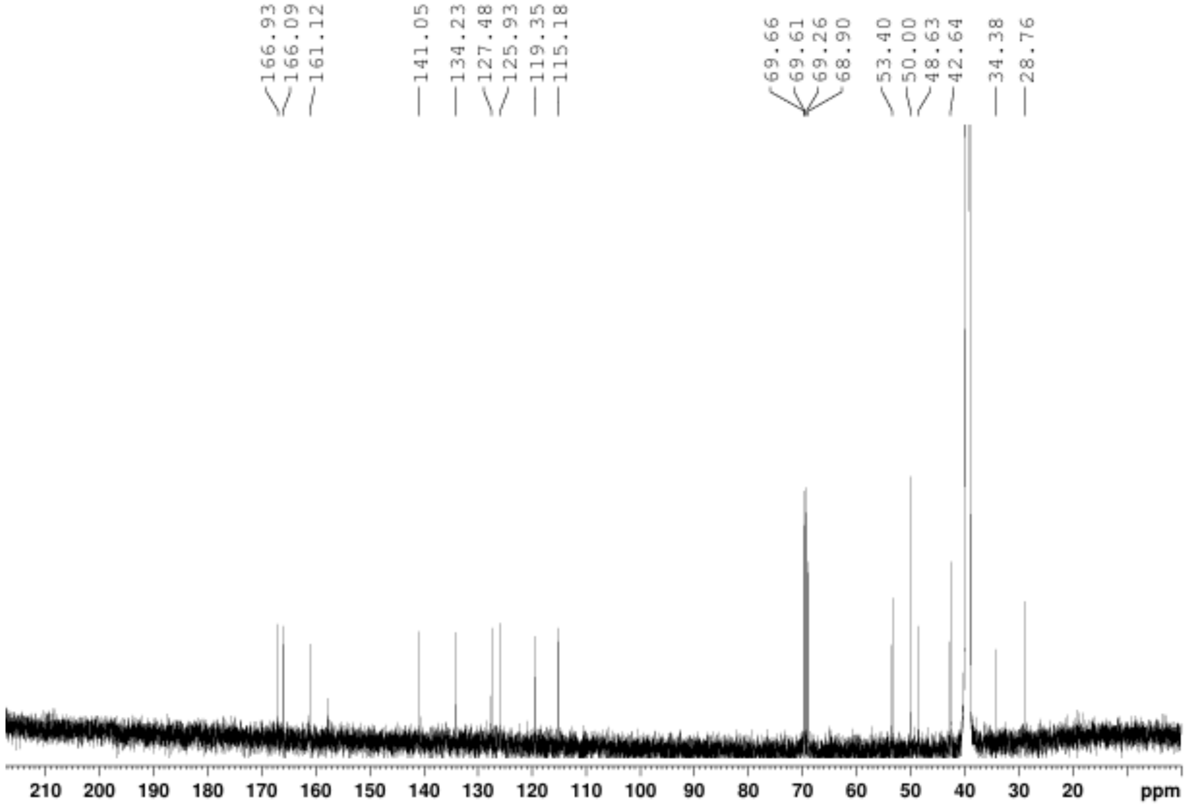

Compound-**30**-^{1}H [DMSO-*d*6]

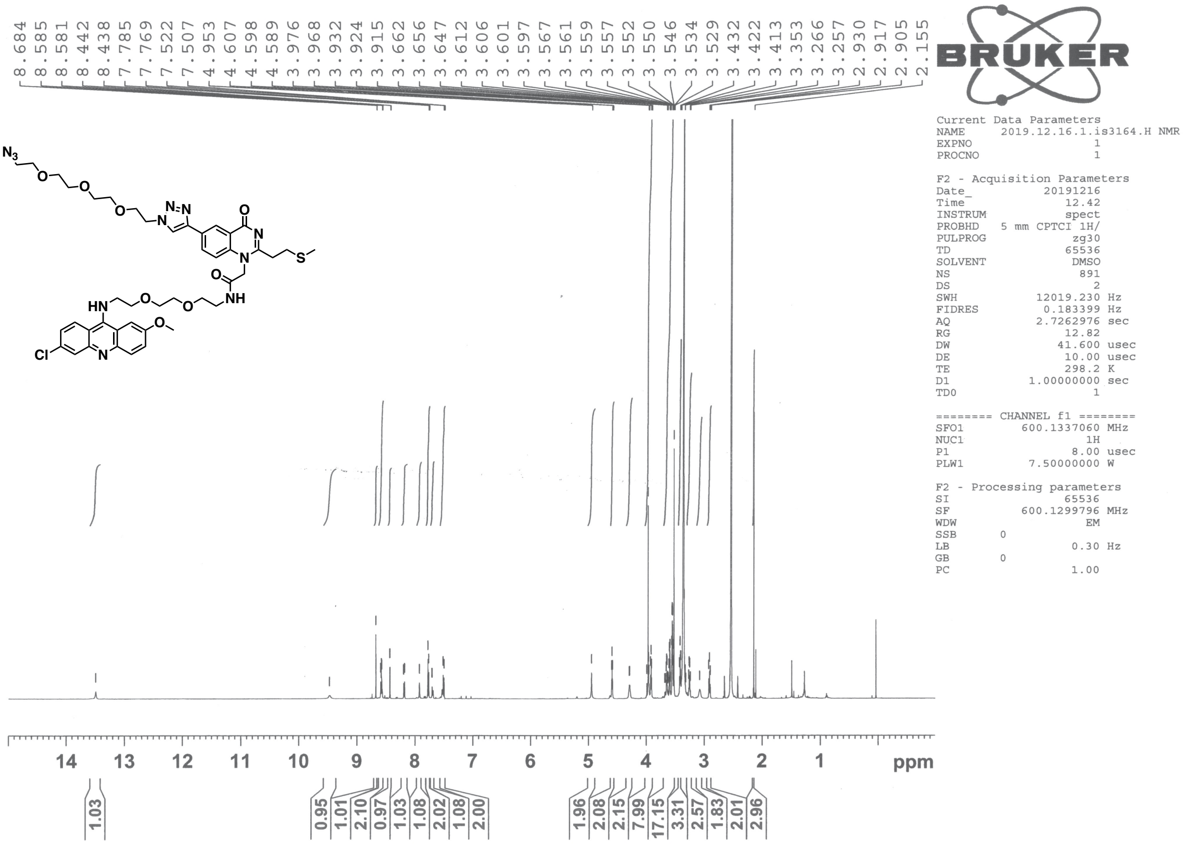

Compound-**30**-^{13}C [CD_3OD]

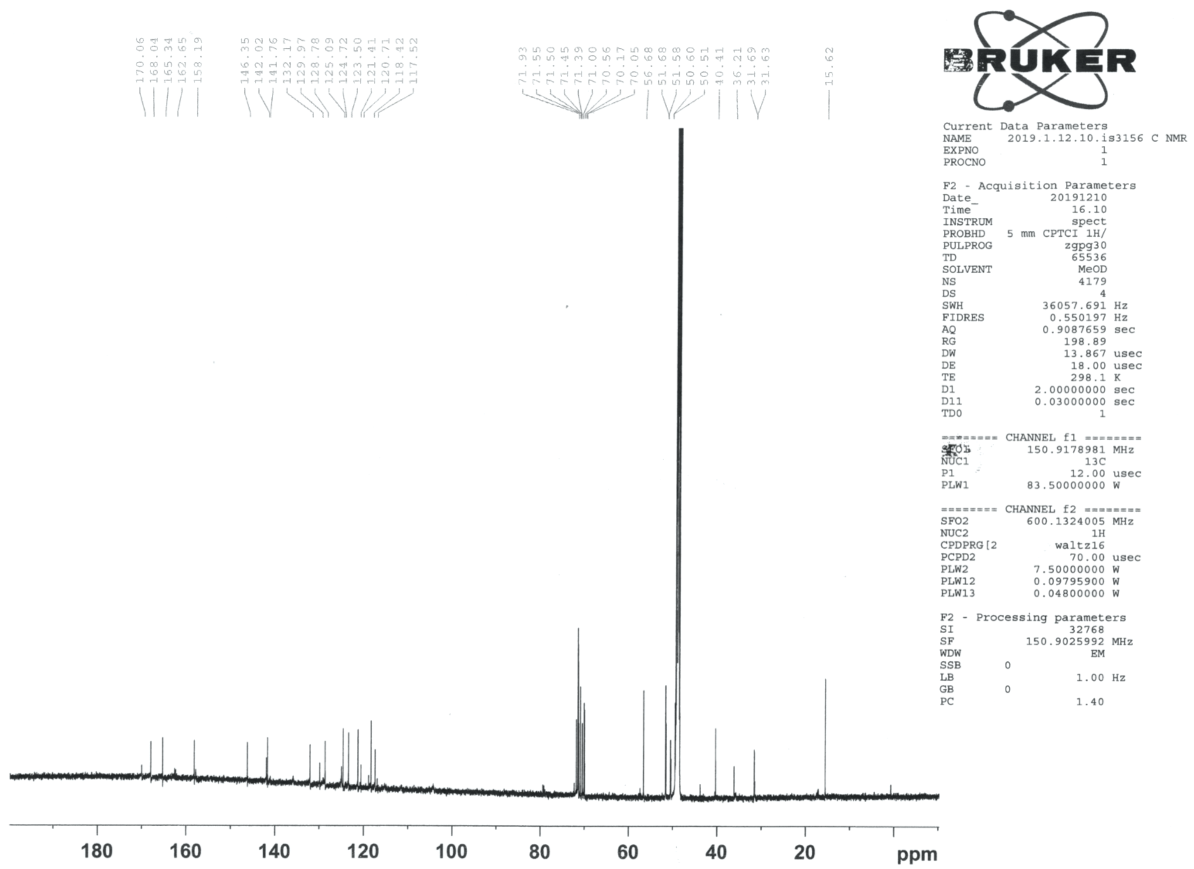

Supplementary References

1. Stamos, J. L., Lentzsch, A. M. & Lambowitz, A. M. Structure of a thermostable group II intron reverse transcriptase with template-primer and its functional and evolutionary implications. *Mol. Cell* **68**, 926-939.e4 (2017).
2. Siegfried, N. A., Busan, S., Rice, G. M., Nelson, J. A. E. & Weeks, K. M. RNA motif discovery by SHAPE and mutational profiling (SHAPE-MaP). *Nat. Methods* **11**, 959–965 (2014).
3. Zubradt, M. *et al.* DMS-MaPseq for genome-wide or targeted RNA structure probing in vivo. *Nat. Methods* **14**, 75–82 (2017).
