# Supplementary material for: Systematic identification of variant-specific RNA structure-small molecule interactions exemplified by RNA G-quadruplexes
Source: Nat Commun. 2026 Mar 19;17:2243. doi: 10.1038/s41467-026-70097-9 (PMC13002888; doi:10.1038/s41467-026-70097-9)
Supplement: Supplementary file 2 — Description of Additional Supplementary Files [file 41467_2026_70097_MOESM2_ESM.pdf]

Description of Additional Supplementary Files

Supplementary Data 1: 5′ UTR somatic mutation library

Supplementary Data 2: Single-RNA library for BIVIDMaP

Supplementary Data 3: G4 and nonG4 containing library for BIVID-MaP

Supplementary Data 4: RNA sequences for gel shift assay

Supplementary Data 5: RNA sequences for CD-spectrum measurement

Supplementary Data 6: RNA sequences for AS-MS

Supplementary Data 7: RNA sequences for G4 sensing assay
