# Supplementary material for: Systematic identification of variant-specific RNA structure-small molecule interactions exemplified by RNA G-quadruplexes
Source: Nat Commun. 2026 Mar 19;17:2243. doi: 10.1038/s41467-026-70097-9 (PMC13002888; doi:10.1038/s41467-026-70097-9)
Supplement: Supplementary file 10 — Reporting Summary [file 41467_2026_70097_MOESM10_ESM.pdf]

nature portfolio

Corresponding author(s): Kaoru R. Komatsu, Hirohide Saito, Kazumitsu Onizuka

Last updated by author(s): Jan 27, 2026

Reporting Summary

Nature Portfolio wishes to improve the reproducibility of the work that we publish. This form provides structure for consistency and transparency in reporting. For further information on Nature Portfolio policies, see our Editorial Policies and the Editorial Policy Checklist.

Statistics

For all statistical analyses, confirm that the following items are present in the figure legend, table legend, main text, or Methods section.

n/a	Confirmed	
☐	☒	The exact sample size (*n*) for each experimental group/condition, given as a discrete number and unit of measurement
☐	☒	A statement on whether measurements were taken from distinct samples or whether the same sample was measured repeatedly
☐	☒	The statistical test(s) used AND whether they are one- or two-sided<br>*Only common tests should be described solely by name; describe more complex techniques in the Methods section.*
☒	☐	A description of all covariates tested
☐	☒	A description of any assumptions or corrections, such as tests of normality and adjustment for multiple comparisons
☐	☒	A full description of the statistical parameters including central tendency (e.g. means) or other basic estimates (e.g. regression coefficient) AND variation (e.g. standard deviation) or associated estimates of uncertainty (e.g. confidence intervals)
☐	☒	For null hypothesis testing, the test statistic (e.g. *F*, *t*, *r*) with confidence intervals, effect sizes, degrees of freedom and *P* value noted<br>*Give P values as exact values whenever suitable.*
☒	☐	For Bayesian analysis, information on the choice of priors and Markov chain Monte Carlo settings
☒	☐	For hierarchical and complex designs, identification of the appropriate level for tests and full reporting of outcomes
☐	☒	Estimates of effect sizes (e.g. Cohen's *d*, Pearson's *r*), indicating how they were calculated

Our web collection on statistics for biologists contains articles on many of the points above.

Software and code

Policy information about availability of computer code

Data collection	ONGene database, TSGene 2.0, COSMIC v98, MANE v1.3, GENCODE v44, MANE (Matched Annotation from NCBI and EMBL-EBI) database (version 1.3)
Data analysis	G4 RNA Screener [http://scottgroup.med.usherbrooke.ca/G4RNA_screener/], UCSC Genome Browser (Table Browser)<br>Custom scripts for classifying sequencing reads and calculating RT deletions per variant are available on GitHub (https://github.com/BIVID-MaP/BIVID_MaP2025) and archived on Zenodo (https://doi.org/10.5281/zenodo.18374906).

For manuscripts utilizing custom algorithms or software that are central to the research but not yet described in published literature, software must be made available to editors and reviewers. We strongly encourage code deposition in a community repository (e.g. GitHub). See the Nature Portfolio guidelines for submitting code & software for further information.

Data

Policy information about availability of data

All manuscripts must include a data availability statement. This statement should provide the following information, where applicable:

- Accession codes, unique identifiers, or web links for publicly available datasets
- A description of any restrictions on data availability
- For clinical datasets or third party data, please ensure that the statement adheres to our policy

All data supporting the findings of this study are available within the Article, its Supplementary Information, and the accompanying Source Data files.

To design the 5′ UTR somatic-mutation library, we retrieved oncogene annotations from the ONGene database and tumor suppressor gene annotations from the TSGene 2.0 database. Somatic mutation data were obtained from the COSMIC database (v. 98). Corresponding transcripts were chosen from the MANE (Matched Annotation from NCBI and EMBL-EBI) database . Corresponding 5′ UTR sequences were extracted from GENCODE v44 annotations using the UCSC Genome Browser (Table Browser).

Research involving human participants, their data, or biological material

Policy information about studies with human participants or human data. See also policy information about sex, gender (identity/presentation), and sexual orientation and race, ethnicity and racism.

Reporting on sex and gender	Not applicable. No human participants were involved, and no sex- or gender-related data were collected.
Reporting on race, ethnicity, or other socially relevant groupings	Not applicable. This study did not involve human participants, and no data on race, ethnicity, or other socially relevant groupings were collected.
Population characteristics	Not applicable. No human population was studied.
Recruitment	Not applicable. No human participants were recruited.
Ethics oversight	Not applicable. Ethics approval and informed consent were not required because no human participants, identifiable human data, or human biological materials were used.

Note that full information on the approval of the study protocol must also be provided in the manuscript.

Field-specific reporting

Please select the one below that is the best fit for your research. If you are not sure, read the appropriate sections before making your selection.

☒ Life sciences ☐ Behavioural & social sciences ☐ Ecological, evolutionary & environmental sciences

For a reference copy of the document with all sections, see nature.com/documents/nr-reporting-summary-flat.pdf

Life sciences study design

All studies must disclose on these points even when the disclosure is negative.

Sample size	No statistical methods were used to predetermine sample size. Sample sizes were chosen based on standard practice in the field and previous experience with similar experiments. Sequencing-based experiments were performed in two independent replicates (n = 2). AS-MS measurements and NMM fluorescence assays were performed in three independent experiments (n = 3); for NMM fluorescence, each experiment included two technical replicates. Gel-shift assays regarding MVK-accelerating modifications were performed in three independent experiments (n = 3). Other gel-shift assays involving previously established labeling chemistries were performed with technical triplicates.
Data exclusions	In the large-scale analysis using the 5′ UTR somatic-mutation library, we excluded sequences with fewer than 1000 reads and genes in which more than 30% of all deletions occurred at the variant position.
Replication	Sequencing experiments were performed in two independent replicates with consistent trends. AS-MS measurements were performed in three independent experiments with consistent results. NMM fluorescence assays were performed in three independent experiments, each with two technical replicates, and showed consistent results. Gel-shift assays assessing MVK-accelerating modifications were performed in three independent experiments, with consistent results. Other gel-shift assays involving previously established labeling chemistries were performed with technical triplicates, yielding consistent results.
Randomization	Not applicable. All assays were conducted in vitro without random allocation to groups
Blinding	Blinding was not applicable, as all outcomes were evaluated quantitatively based on objective measurements.

Reporting for specific materials, systems and methods

We require information from authors about some types of materials, experimental systems and methods used in many studies. Here, indicate whether each material, system or method listed is relevant to your study. If you are not sure if a list item applies to your research, read the appropriate section before selecting a response.

Materials & experimental systems

n/a	Involved in the study
☒	☐ Antibodies
☒	☐ Eukaryotic cell lines
☒	☐ Palaeontology and archaeology
☒	☐ Animals and other organisms
☒	☐ Clinical data
☒	☐ Dual use research of concern
☒	☐ Plants

Methods

n/a	Involved in the study
☒	☐ ChIP-seq
☒	☐ Flow cytometry
☒	☐ MRI-based neuroimaging

Plants

Seed stocks

Report on the source of all seed stocks or other plant material used. If applicable, state the seed stock centre and catalogue number. If plant specimens were collected from the field, describe the collection location, date and sampling procedures.

Novel plant genotypes

Describe the methods by which all novel plant genotypes were produced. This includes those generated by transgenic approaches, gene editing, chemical/radiation-based mutagenesis and hybridization. For transgenic lines, describe the transformation method, the number of independent lines analyzed and the generation upon which experiments were performed. For gene-edited lines, describe the editor used, the endogenous sequence targeted for editing, the targeting guide RNA sequence (if applicable) and how the editor was applied.

Authentication

Describe any authentication procedures for each seed stock used or novel genotype generated. Describe any experiments used to assess the effect of a mutation and, where applicable, how potential secondary effects (e.g. second site T-DNA insertions, mosiacism, off-target gene editing) were examined.
